# Global, regional, and national trends in depressive disorder prevalence and DALYs among women of childbearing age from 1990 to 2021 and projections to 2040: a comprehensive analysis from 1990 to 2021

**DOI:** 10.3389/fgwh.2025.1629747

**Published:** 2025-08-18

**Authors:** Yuhang Yang, Yuyuan Hu, Yuan He, Wei Zhang, Jinghan Jia, Yibo Xu, Yan Li, Jinxi Wang

**Affiliations:** ^1^ Division of Colorectal Surgery, Third Hospital of Shanxi Medical University, Shanxi Bethune Hospital, Shanxi Academy of Medical Sciences Tongji Shanxi Hospital, Taiyuan, China; ^2^ Hepatobiliary Surgery, Baogang Hospital of InnerMongolia, Baotou, China; ^3^ Faculty of Clinical Medicine, Xi’an Medical University, Xi’an, China; ^4^ Neurology, Third Hospital of Shanxi Medical University, Shanxi Bethune Hospital, Shanxi Academy of Medical Sciences Tongji Shanxi Hospital, Taiyuan, China

**Keywords:** depression, women of childbearing age, burden of disease, joinpoint regression analysis, age-period-cohort analysis

## Abstract

**Purpose:**

This study aims to explore global, regional, and national trends in the prevalence of depression and disability-adjusted life years (DALYs) among women of childbearing age from 1990 to 2021, as well as to project future trends from 2022 to 2040.

**Methods:**

This research analyzes the prevalence of depression and Disability-Adjusted Life Years (DALYs) among Women of Childbearing Age (WCBA) using data from the Global Burden of Disease (GBD) database, covering the period from 1990 to 2021. We evaluate trends in the burden of depression in WCBA through estimated annual percentage change (EAPC) and percentage change, as well as annual percentage change (APC) and average annual percentage change (AAPC) derived from Joinpoint regression analyses. Additionally, we employ age-period-cohort modeling to provide a more comprehensive evaluation of the prevalence of WCBA and the burden of DALYs, including future projections.

**Results:**

In 2021, it was estimated that there were 121.24 million cases of depression among women of childbearing age worldwide, with disability-adjusted life years (DALYs) accounting for 21.04 million cases. When compared to figures from 1990, these numbers indicate percentage increases of 68% and 69%, respectively. Moreover, despite an overall increase in both global prevalence and DALY rates, this rise was not considered statistically significant, as reflected by an estimated annual percentage change (EAPC) of −0.02 (95% confidence interval: −0.17 to 0.13) for prevalence and −0.06 (95% CI: −0.24 to 0.12) for DALYs. Furthermore, the average annual percentage change (AAPC) was computed to be 0.4789 (95% CI: 0.3289–0.6295; *P* < 0.001) for prevalence and 0.524 (95% CI: 0.3756–0.6725; *P* < 0.001) for DALYs. Projections made using our Bayesian age–period–cohort (BAPC) model suggest that we can expect a considerable increase in the global prevalence of depression and DALY rates among women of childbearing age by the year 2040.

## Introduction

Depression is one of the common mental disorders. It is a mood disorder characterized by a significant and persistent feeling of sadness and despair lasting for two weeks or more, leading individuals to feel that life is no longer worth living or to experience a loss of interest/pleasure. It is also accompanied by significant changes in behavior, physical health, and social functioning. Although depression has been subdivided into major depressive disorder (MDD), dysthymia, and depressive subtypes within bipolar disorder, existing research still lacks a systematic assessment of the overall epidemiological burden of depression. Therefore, this study aims to fill this gap. According to estimates from the World Health Organization (WHO), 3.8% of the global population is affected by depression, with a prevalence approximately 50% higher in women compared to men (1, 2), placing a heavy burden on the global public health system. Women of childbearing age (WCBA, 15−49 years) exhibit a significantly higher prevalence of depression. This heightened risk can be linked to an interplay of biological, psychological, and social influences that are specific to this population. For instance, hormonal changes may greatly affect mood and emotional stability. Moreover, the pressures related to pregnancy can intensify feelings of anxiety and depression. As women also adapt to evolving social roles during this crucial time, the demands to fulfill various expectations can further undermine their mental health. Collectively, these factors underscore the intricate relationships that elevate the likelihood of depression in this demographic group (3). Worldwide, depression ranks among the primary contributors to disability-adjusted life years (DALYs) among women. However, there is a notable deficiency in comprehensive research that thoroughly investigates the impact and patterns of depression in women of reproductive age. The Global Burden of Disease (GBD) 2021 study emphasizes the significance of mental disorders, particularly depression, in the global Disability-Adjusted Life Years (DALY) rankings for the 15−49 age group. Alarmingly, studies show that more than 25% of WCBA are diagnosed with depression prior to pregnancy (4), and more than 10% of pregnant women and those who have recently given birth suffer from depression (5, 6), which can exacerbate the risk of adverse maternal and neonatal outcomes, including preterm birth, low birth weight, and impaired child development. Despite its far-reaching effects, depression is frequently underdiagnosed and undertreated among WCBA, particularly in resource-limited countries. This is largely due to the stigmatization of mental illness, fragmented healthcare systems, and the insufficient prioritization of mental health within obstetric care.

Multiple risk factors converge to contribute to the burden of depression among WCBA. The prevalence of depression in this demographic is influenced not only by age but also by the social, policy, and economic contexts of both the era and the birth cohort. Women during childhood may experience irreversible mental health effects stemming from unfavorable family and school environments. In contrast, multiple factors such as gender inequality, disparities in access to mental health resources, socio-cultural norms and gender stigma, employment and economic instability, lack of supportive policies for balancing work and family, as well as migration and forced displacement, may subject young and middle-aged women to potentially irreversible psychological trauma. With the development of society, social factors such as abortion bans targeting women and the wage gap and unequal distribution of economic resources between men and women have significantly increased the risk of depression among women (7). In addition, factors such as low socioeconomic status, lack of social support, insufficient empowerment, pressure from expectations regarding fetal gender, and racial factors are all closely associated with the occurrence of perinatal depression (8). When family support is weak, experiences of gender discrimination exacerbate depressive symptoms; and as women's motivation to conceal increases, coupled with further reduction in support from partners and family, the severity of depression also deepens (9). Unstable employment, domestic violence, and displacement caused by war and conflict make adolescent girls more susceptible to mental distress (10, 11). Work-family conflict has been proven to impair mothers' mental health. Therefore, workplaces need to implement family-friendly policies to help parents balance their careers and family responsibilities, thereby effectively alleviating role conflicts (12). Existing research indicates that risk factors for modifiable daily life experiences and behaviors also influence the prevalence of WCBA, with intimate partner violence (IPV), child sexual abuse and bullying (CSAB), child sexual abuse (CSA), and bullying victimization (BV) all significantly associated with WCBA with depression (13). Controlling for modifiable lifestyle behavioral risk factors in vulnerable populations may reduce the prevalence of women of childbearing age (WCBA). Therefore, assessing the burden of depression among WCBA is crucial for initiating targeted intervention activities on a global scale. Existing literature has primarily concentrated on depression in the general population or in specific subgroups, such as adolescents or postpartum women (14, 15). However, it lacks a systematic assessment of longitudinal trends, socio-demographic differences, and age-specific patterns in the burden of depression in the global WCBA. While high-income regions report a higher prevalence of depression attributed to enhanced diagnostic capabilities, low- and middle-income countries experience underreporting despite the rising psychosocial stressors linked to urbanization, economic inequality, and gender-based violence. Furthermore, the interactions between depression and reproductive health—such as hormonal contraceptive use, menstrual cycle, and fertility challenges—remain underexplored in large-scale epidemiological studies. This gap hinders the development of targeted interventions that address the unique needs of WCBA, particularly in regions with limited mental health infrastructure.

The United Nations Sustainable Development Goal 3 (SDG 3) emphasizes the necessity of reducing maternal mortality and achieving universal access to reproductive health care. However, the impact of depression on these objectives is frequently overlooked. Depressive symptoms among WCBA are linked to decreased adherence to antenatal care, an increased risk of obstetric complications, and intergenerational health disparities (16, 17). This highlights the urgent need to integrate mental health into the maternal health framework. Utilizing data from the Global Burden of Disease Study 2021, this study aims to provide the first comprehensive analysis of the prevalence of depression and DALYs among WCBA across 204 countries and territories from 1990 to 2021. By evaluating trends based on SDI, age strata, and risk factors, this research seeks to illuminate the evolving burden of depression in WCBA and identify key areas for policy intervention. Our findings will inform global strategies aimed at reducing the dual burden of mental and reproductive health challenges, advancing progress towards Sustainable Development Goal 3 (SDG 3), and ensuring equitable access to healthcare for women navigating critical life stages.

## Methods

### Data collection

The WCBA depression data analyzed in this study were sourced from the Global Burden of Disease (GBD) 2021 database, which offers extensive information on the global and regional burdens of 371 diseases, injuries, and 88 risk factors across 21 GBD regions and 204 countries and territories from 1990 to 2021. All of this data are freely accessible through the Global Health Data Exchange (https://ghdx.healthdata.org/gbd-2021/sources) (18). GBD 2021 estimated the point prevalence (hereinafter referred to as prevalence), incidence, mortality, years of life lost (YLLs), years lived with disability (YLDs), and disability-adjusted life years (DALYs) for 371 diseases and injuries across 204 countries and territories. All raw data were obtained from sources including population censuses, household sample surveys, disease-specific surveillance, medical service records, verbal autopsies, disease and civil registration, and other sources. The GBD study team systematically adjusted epidemiological data to eliminate biases arising from different sources, definitions, and measurement methods. These adjustments were made using the Bayesian meta-regression modeling tool DisMod-MR 2.1, which integrates and corrects data from diverse global sources, varying in quality and definitions, ultimately producing unified estimates of incidence, prevalence, remission rates, case fatality rates, excess mortality, and DALYs. This approach minimizes the impact of heterogeneity on the conclusions (19, 20). According to the International Classification of Diseases, 10th Revision (ICD), depression is classified under codes F32.0-9 and F33.0-9. In the DSM-IV-TR classification, it is coded as 296.21-24 and 296.31-34, respectively (13).

### Socio-demographic index (SDI)

The Socio-Demographic Index (SDI) is a comprehensive measure of a country or region's socio-economic development level, developed in 2015 by the Global Burden of Disease (GBD) team. It was created to address the limitations of traditional economic metrics, such as GDP, in health research and to quantify the impacts of social, economic, and demographic factors on health. The SDI serves to assess the state of development that is strongly correlated with health outcomes, thereby providing a scientific basis for public health research. The Socio-Demographic Index (SDI) ranges from 0 to 1, indicating varying levels of development, with 0 representing the lowest and 1 the highest. This index is constructed based on three key indicators: the total fertility rate (TFR) for individuals under 25 years of age (TFU25), the average educational attainment of individuals aged 15 and older, and the lagged distribution of per capita income (LDI). The original covariates—TFR for ages 15–49, average educational attainment for individuals aged 15, and the lagged distribution of income per capita—are scaled using the minimum and maximum values observed during the estimation period. In the Global Burden of Disease (GBD) 2021 study, the 204 countries and territories are classified into five SDI regions: low SDI (SDI <0.46), low-middle SDI (0.46 ≤SDI <0.61), middle SDI (0.61 ≤SDI <0.70), high-middle SDI (0.70 ≤SDI ≤0.81), and high SDI (SDI >0.8) (18).

### Disability adjusted life years (DALYs)

Disability-Adjusted Life Years (DALYs) is a composite indicator that quantifies the total healthy life years lost due to morbidity and mortality. It encompasses both Years of Life Lost due to Disability (YLLs) and Years Lost due to Disability (YLDs). The following formula is designed to quantify the impact of disease on population health (18).

### Estimated annual percentage change and percentage change

Estimated Annual Percentage Change (EAPC) is an important indicator for assessing trends in disease burden prevalence and DALYs (21). This study aims to estimate the dynamic trends in the prevalence of depression and Disability-Adjusted Life Years (DALYs) from 1990 to 2021. The Estimated Annual Percentage Change (EAPC) was calculated using a mathematical model, such as log-linear regression or Poisson regression. This involved fitting the natural logarithm of each observation to a linear regression model, thereby generating a straight line with the year series as the independent variable. The slope of this line represents the year-on-year rate of change, and subsequent calculations are based on this slope.


y=α+βx+ε

EAPC=100×(exp(β)−1)

*x* years, *y*—natural logarithm of the rate (e.g., prevalence), *α*—intercept, *β*—slope, *ε* random error. The EAPC and its 95% Confidence Interval (CI) were derived using the aforementioned equation. A lower limit of the 95% CI of the EAPC that exceeds 0 indicates an increasing trend within the studied interval. Conversely, an upper limit of the 95% CI of the EAPC that falls below 0 suggests a decreasing trend during the same period. If the 95% CI of the EAPC encompasses 0, it signifies that the change in trend is not statistically significant (22). In this study, we utilized the percentage change from 1990 to 2021 to evaluate the variation in the number of prevalent cases and the cases of Disability-Adjusted Life Years (DALYs).


Percentagechange=(2021cases−1990cases)/1990cases

### Risk factor analysis

The GBD 2021 database was analyzed to identify risk factors associated with the burden of depression among women of childbearing age (WCBA). Disability-adjusted life years (DALYs) attributable to each risk factor were synthesized. To visualize the contribution of each risk factor to the burden of depression, comparison bar charts were created using the tidyverse and ggplot2 packages in R version 4.4.2 (23–25).

### Statistical analysis

Joinpoint regression analysis: Significant changes in the time trends of depression prevalence and DALYs from 1990 to 2021 were assessed using the Joinpoint regression analysis model, Monte Carlo permutation test regression analysis, and the calculation of Annual Percentage Change (APC) and Mean Annual Percentage Change (AAPC) along with their 95% CI. An APC or AAPC estimate, along with the lower limit of its 95% CI greater than 0 and a *p*-value less than 0.05, indicates an increasing trend within a specified interval. Conversely, an APC or AAPC estimate combined with an upper limit of its 95% CI less than 0 and a *p*-value less than 0.05 indicates a decreasing trend within a specified interval. When the 95% CI for APC or AAPC includes 0 and the *p*-value is greater than or equal to 0.05, the trend is considered stable (26–28).

Age-period-cohort analysis: This study aims to assess changes in the prevalence and DALYs rates associated with depression in the global population of WCBA, aged 15–49 years, from 1990 to 2021. Additionally, it examines the effects of age, period, and cohort on the prevalence of depression and its associated DALYs rates. To project the burden of disease from 2022 to 2040, Bayesian age-period-cohort (BAPC) modeling was utilized.

All statistical processes were conducted using R Studio version 4.4.2 for data cleaning, calculations, organization, and analysis. The results are presented as means with 95% uncertainty intervals (UI), and all rates are expressed per 100,000 population. The dplyr and ggmap packages were utilized to create a world map for data visualization.

## Results

### Global level

**Figure 1:**
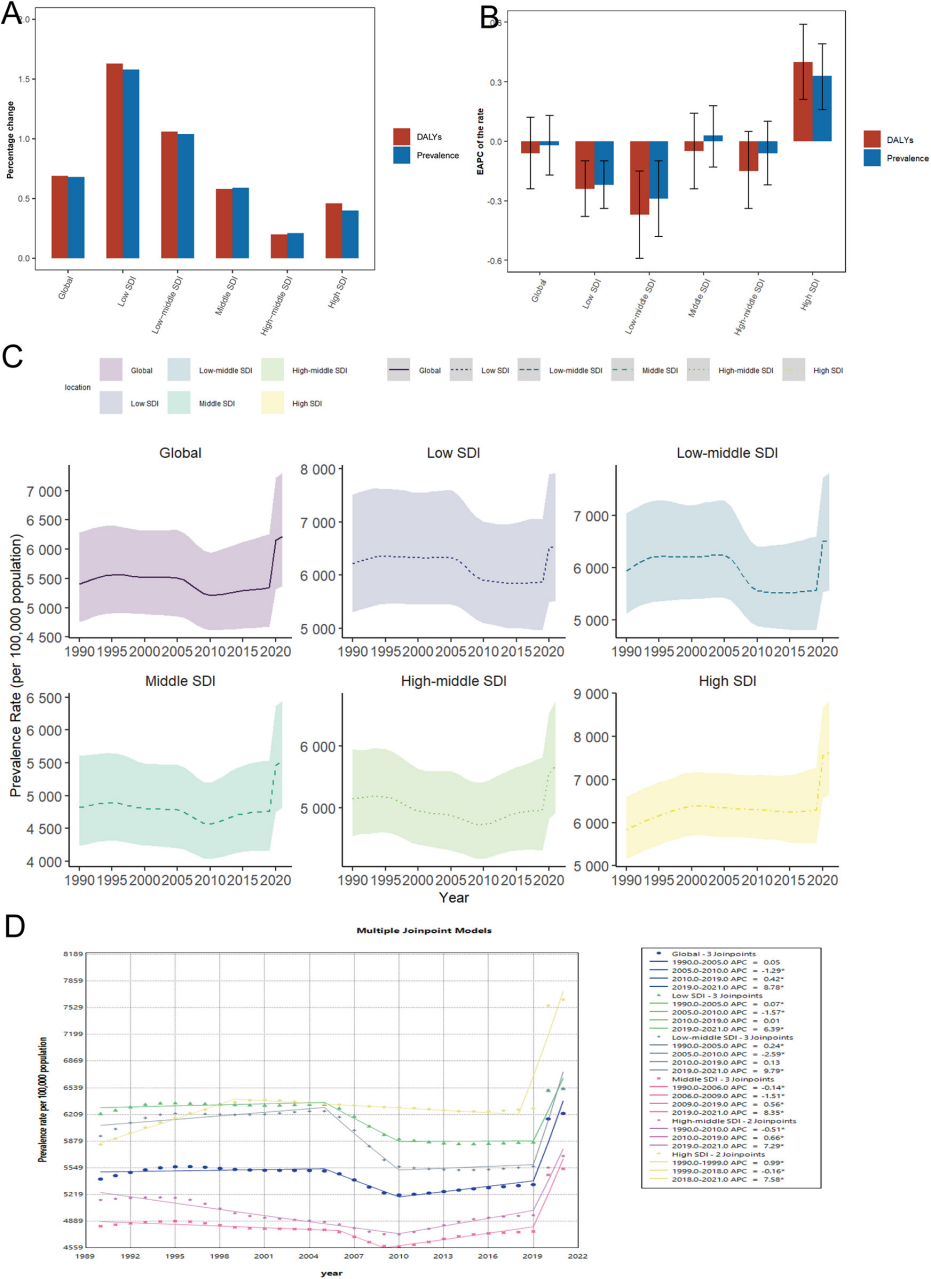
Temporal trends in the burden of depression in WCBA globally and in 5 regions. **(A)** Percentage change in prevalence and DALY cases between 1990 and 2021. **(B)** The EAPC of prevalence and DALY rates from 1990 to 2021. **(C)** The prevalence rates from 1990 to 2021. **(D)** Joinpoint regression analysis of time trends in prevalence rates from 1990 to 2021. WCBA, women of childbearing age; EAPC, estimated annual percentage change; DALYs, disability-adjusted life years; AAPC, average annual percentage change; APC, annual percentage change.

The number of prevalent cases of depression and the number of DALYs in the global WCBA increased significantly from 1990 to 2021. For example, the number of prevalent cases grew from 72.35 million cases (95% UI: 63.63–84.07 million) in 1990 to 121.24 million cases (95% UI: 104.69–142.43 million) in 2021, with a percentage change of 68% in the number of prevalent cases, and the number of DALYs grew from 12.45 million cases (95% UI: 8.43–17.07 million) in 1990 to 21.04 million cases (95% UI: 14.07–29.06 million) in 2021, with a percentage change in the number of DALYs of 69% (Table 1; Supplementary Table S1; Figure 1A). Although ostensibly the 1990 prevalence rate grew from 5,409.59 cases per 100,000 population (95% UI: 4,757.83–6,285.95) to 6,220.98 cases per 100,000 population in 2021 (95% UI: 5,371.96–7,308.26) and the 1990 DALYs rate grew from 930.59 cases per 100,000 population (95% UI: 630.46–1,276.48) to 1,079.73 cases per 100,000 population (95% UI: 722.08–1,491.21) in 2021 but with an EAPC of −0.02 (95% CI: −0.17 to 0.13) and −0.06 (95% CI: −0.24 to 0.12), respectively (Table 1; Supplementary Table S1; Figures 1B,C; Supplementary Figure S1), the global burden of absolute prevalence and DALYs rates is increasing but the upward trend is not significant. Joinpoint regression analysis showed an overall slight increase in global WCBA depression prevalence from 1990 to 2021 AAPC = 0.4789 (95% CI: 0.3289–0.6295; *P* < 0.001), with a significant increasing trend from 2019 to 2021 APC = 8.7824 (95% CI: 6.7558–10.8476; *P* < 0.001) (Supplementary Table S2; Figure 1D). Similarly, from 1990 2021, DALYs followed the same trend AAPC = 0.524 (95% CI: 0.3756–0.6725; *P* < 0.001), and also showed a significant increasing trend from 2019 to 2021 APC = 10.308 (95% CI: 8.3798 −12.2706; *P* < 0.001) (Supplementary Table S3, Supplementary Figure S2).

**Table 1:** The prevalence of depression cases and rates among WCBA (15–49 years) in 1990 and 2021,and the trends from 1990 to 2021.

Location	Prevalent cases			Prevalent rates		
	1990_millions (95% UI)	2021_millions (95% UI)	Percentage change (100%)	1990_per100 000 (95% UI)	2021_per100 000 (95% UI)	EAPC(95% CI)
Global	72.35 (63.63–84.07)	121.24 (104.69–142.43)	0.68	5,409.59 (4,757.83–6,285.95)	6,220.98 (5,371.96–7,308.26)	−0.02 (−0.17−0.13)
Low SDI	6.95 (5.92−8.39)	17.91 (15.15–21.73)	1.58	6,220.59 (5,303.39–7,508.24)	6,529.33 (5,523.71–7,920.21)	-0.22 (−0.34–0.1)
Low-middle SDI	16.22 (14.01–19.22)	33.06 (28.24–39.58)	1.04	5,942.14 (5,133.65–7,041.56)	6,530.56 (5,578.11–7,818)	-0.29 (−0.48–0.1)
Middle SDI	21.58 (18.93–25.06)	34.25 (29.81–39.83)	0.59	4,826.4 (4,235.27–5,606.44)	5,537.56 (4,819.56–6,439.61)	0.03 (−0.13–0.18)
High-middle SDI	14.31 (12.64–16.52)	17.38 (15.04–20.51)	0.21	5,151.68 (4,550.17–5,948.93)	5,694.63 (4,928.95–6,722.86)	-0.06 (−0.22–0.1)
High SDI	13.23 (11.7–14.99)	18.55 (16.17–21.44)	0.4	5,837.1 (5,158.74–6,612.15)	7,629.55 (6,650.03–8,819.17)	0.33 (0.16–0.49)
Andean Latin America	0.39 (0.33–0.48)	0.94 (0.76–1.17)	1.41	4,163.65 (3,453.48–5,104.23)	5,381.39 (4,343.22–6,721.89)	0.18 (−0.11–0.47)
Australasia	0.41 (0.36–0.47)	0.6 (0.49–0.75)	0.46	7,598.14 (6,621.33–8,749.73)	8,370.39 (6,744.14–10,337.36)	0.17 (0.03–0.3)
Caribbean	0.58 (0.48–0.7)	0.79 (0.64–1)	0.36	6,185.61 (5,170.89–7,460.83)	6,541.12 (5,292.75–8,343.63)	−0.33 (−0.54–0.13)
Central Asia	0.75 (0.62–0.9)	1.28 (1.05–1.57)	0.71	4,443.18 (3,709.54–5,363.97)	5,290.44 (4,324.48–6,473.68)	0.23 (0.09–0.36)
Central Europe	1.32 (1.13–1.56)	1.27 (1.07–1.51)	−0.04	4,297.78 (3,665.45–5,065.5)	4,918.48 (4,152.19–5,860.12)	−0.15 (−0.35–0.06)
Central Latin America	1.78 (1.51–2.13)	4.31 (3.65–5.17)	1.42	4,235.61 (3,596.9–5,086.17)	6,326.59 (5,352.16–7,575.82)	0.97 (0.8–1.15)
Central Sub-Saharan Africa	1.06 (0.87–1.33)	2.95 (2.36–3.78)	1.78	8,592.02 (7,039.79–10,799.24)	9,031.63 (7,215.44–11,573.59)	−0.01 (−0.1–0.08)
East Asia	15.02 (13.19–17.28)	13.47 (11.73–15.7)	−0.1	4,505.86 (3,955.42–5,183.76)	4,070.97 (3,545.24–4,745.6)	−0.51 (−0.67–0.35)
Eastern Europe	2.86 (2.45–3.37)	3.12 (2.64–3.65)	0.09	5,166.42 (4,430.05–6,101.49)	6,464.51 (5,471.4–7,561.7)	0.07 (−0.14–0.27)
Eastern Sub-Saharan Africa	2.86 (2.44–3.43)	7.58 (6.38–9.14)	1.65	6,631.92 (5,650.92–7,951.63)	7,079.99 (5,959.38–8,533.45)	−0.17 (−0.28–0.06)
High-income Asia Pacific	1.6 (1.41–1.81)	1.61 (1.39–1.87)	0.01	3,492.53 (3,084.54–3,950.92)	4,220.09 (3,652.65–4,924.04)	0.22 (0.05–0.4)
High-income North America	5.24 (4.58–5.96)	8.7 (7.62–9.9)	0.66	7,044.62 (6,159.75–8,014.74)	10,350.16 (9,070.74–11,785.55)	0.45 (0.22–0.67)
North Africa and Middle East	5.56 (4.69–6.78)	13.3 (11–16.31)	1.39	7,121.58 (6,005–8,674.7)	8,344.27 (6,906.15–10,236.44)	0.31 (0.18–0.45)
Oceania	0.07 (0.05–0.08)	0.15 (0.12–0.19)	1.14	4,236.98 (3,517.26–5,231.57)	4,438.45 (3,576.58–5,542.11)	−0.01 (−0.05–0.03)
South Asia	15.17 (13.13–17.75)	31.2 (26.58–36.77)	1.06	5,950.73 (5,151.47–6,964.1)	6,313.76 (5,378.48–7,440.85)	−0.57 (−0.8–0.34)
Southeast Asia	4.44 (3.79–5.16)	7.96 (6.82–9.29)	0.79	3,691.84 (3,153.91–4,296.29)	4,344.39 (3,724.45–5,073.59)	0.2 (0.09–0.3)
Southern Latin America	0.71 (0.61–0.85)	1.13 (0.92–1.39)	0.59	5,738.11 (4,936.45–6,855.52)	6,473.18 (5,282.94–7,974.35)	−0.23 (−0.46–0.01)
Southern Sub-Saharan Africa	0.8 (0.69–0.93)	1.66 (1.43–1.95)	1.07	6,003.2 (5,210.95–7,002.01)	7,656.95 (6,568.26–8,965.9)	0.44 (0.23–0.64)
Tropical Latin America	2.59 (2.24–3.04)	4.73 (4.03–5.53)	0.83	6,491.11 (5,604.95–7,616.04)	7,798.27 (6,655.7–9,122.37)	−0.22 (−0.54–0.1)
Western Europe	6.68 (5.95–7.61)	7.76 (6.61–9.33)	0.16	6,993.98 (6,225.99–7,960)	8,325.44 (7,095.16–10,015.26)	0.19 (0.03–0.35)
Western Sub-Saharan Africa	2.47 (2.1–3.01)	6.74 (5.71–8.16)	1.73	5,667.68 (4,815.87–6,907.31)	5,619.07 (4,766.78–6,809)	−0.16 (−0.23–0.08)

WCBA, women of childbearing age; EAPC, estimated annual percentage change; CI, confidence intervals; UI, uncertainty intervals; SDI, socio-demographic index.

### SDI regional level

**Figure 2:**
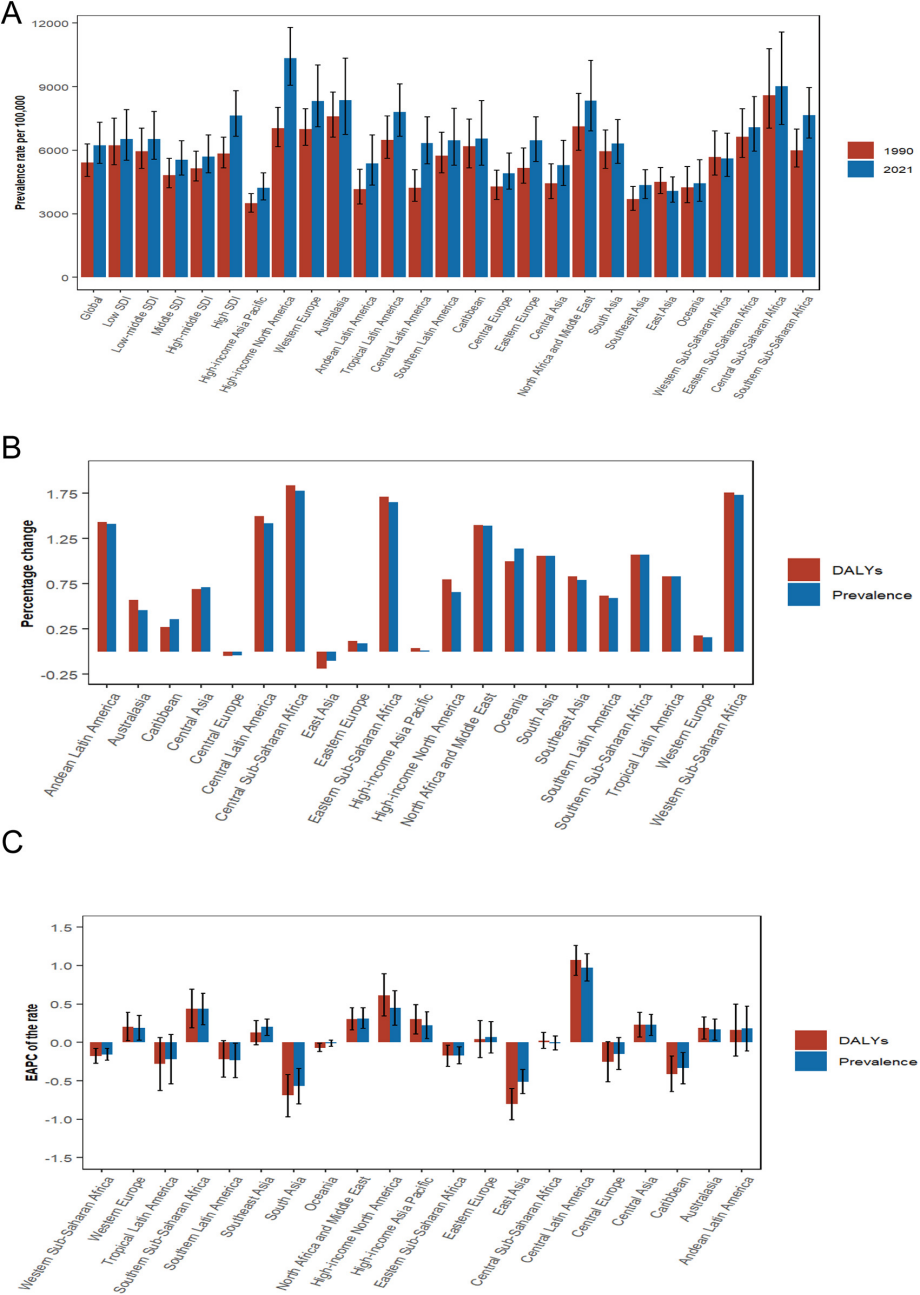
Temporal trends of depression burden in WCBA in regions. **(A)** Prevalence rate per 100,000 population in 1990 and 2021. **(B)** Percentage change in cases of prevalent and DALYs in 1990 and 2021. **(C)** EAPC of rates of prevalence and DALYs from 1990 to 2021.WCBA, women of childbearing age; EAPC, estimated annual percentage change; DALYs, disability-adjusted life years.

In 2021, the highest number of WCBA depression prevalence cases and the highest number of DALYs were in the middle SDI region, with 34.25 million (95% UI: 29.81–39.83 million) and 5.82 million (95% UI: 3.88–7.95 million) cases, respectively, which accounted for approximately one-quarter of the global total. The number of diseased cases and the number of DALYs increased progressively with decreasing SDI, with the largest percentage changes of 158% and 163%, respectively, in low SDI region and the smallest percentage changes of 21% and 20% in high-middle SDI region (Table 1; Supplementary Table S1; Figure 1A). In 2021, prevalence and DALYs rates were highest in high SDI region (Table 1; Supplementary Table S1; Figures 1C–2A; Supplementary Figures S1–S3). Notably, prevalence and DALYs rates increased at the highest rate from 1990 to 2021 in high SDI region compared to other SDI region, with EAPC values of 0.33 (95% UI: 0.16–0.49) and 0.4 (95% UI: 0.21–0.59), respectively, and AAPC values of 0.8997 (95% CI: 0.6688–1.1312; *P* < 0.001) and 1.1262 (95% CI: 1.0556–1.1969; *P* < 0.001), respectively (Table 1; Supplementary Tables S1–S3; Figure 1B). In addition, the prevalence and DALYs rates are relatively high in the middle SDI region in 2021. Thus, the number of depression prevalence and DALYs is highest in the middle SDI region WCBA, and these rates are increasing relatively quickly.

### GBD regional level

A comparative analysis of regions indicates that the prevalence of depression and the associated DALYs among WCBA have generally increased over the years across most regions, with only a few exceptions, specifically East Asia and Central Europe, which are classified as high-middle SDI region. Over the past 32 years, both prevalence and DALYs rates have consistently risen in select regions, notably Central Latin America and high-income North America. The EAPC for prevalence in these regions was 0.97 (95% CI: 0.8–1.15) and 0.45 (95% CI: 0.22–0.67), respectively, while the AAPC was 1.5241 (95% CI: 1.312–1.7366; *P* < 0.001) and 1.3382 (95% CI: 1.1976–1.4791; *P* < 0.001). For DALYs, the EAPC values were 1.07 (95% CI: 0.87–1.26) and 0.61 (95% CI: 0.34–0.89), with AAPC of 1.6654 (95% CI: 1.4376–1.8936; *P* < 0.001) and 1.5849 (95% CI: 1.3028–1.8678; *P* < 0.001), respectively. In contrast, the most significant decreases in prevalence and DALY rates were recorded in East and South Asia, showing EAPC values of −0.51 (95% CI: −0.67 to −0.35) and −0.57 (95% CI: −0.80 to −0.34) for prevalence, and AAPC values of −0.3489 (95% CI: −0.5315 to −0.1678; *P* < 0.001) and 0.2789 (95% CI: 0.1524–0.4055; *P* < 0.001), respectively. The EAPC for DALYs was −0.8 (95% CI: −1.01 to −0.60) and −0.69 (95% CI: −0.97 to −0.42), with AAPC values of −0.6107 (95% CI: −0.8531 to −0.3678; *P* < 0.001) and 0.1782 (95% CI: −0.218 to 0.576; *P* = 0.3785), respectively (Table 1; Supplementary Tables S1-S3).

### National level

**Figure 3:**
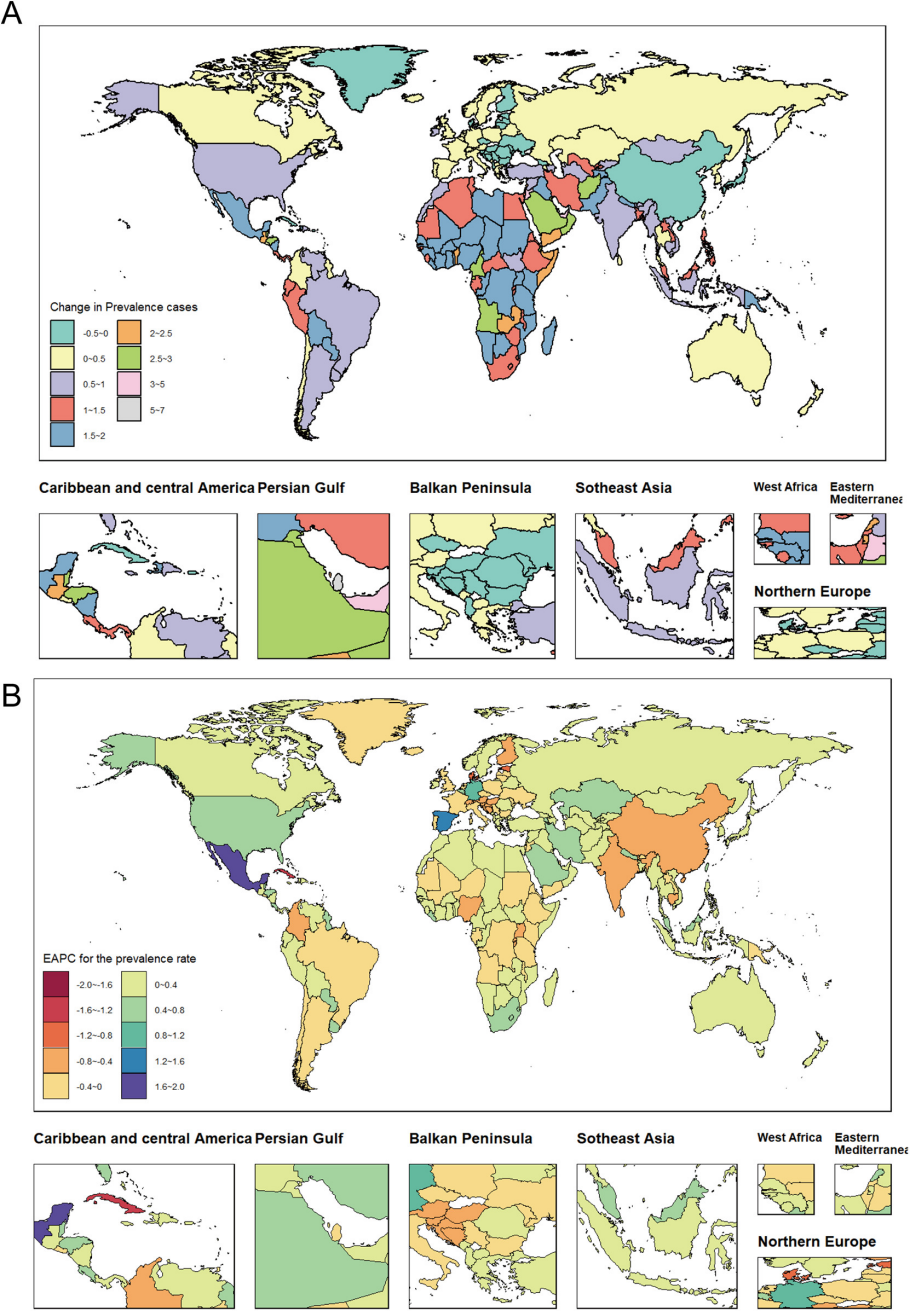
Temporal trends in the global WCBA burden of depression. **(A)** Percentage change in prevalent cases across 204 countries in 1990 and 2021. **(B)** EAPC in prevalent rates across 204 countries from 1990 to 2021. WCBA, women of childbearing age; EAPC, estimated annual percentage change.

From 1990 to 2021, approximately 87% of countries with WCBA experienced an increase in the prevalence of depression and DALYs. Qatar exhibited the highest percentage change, approximately 634%, while the United Arab Emirates followed closely with a change of about 487%. Both countries are categorized within the high SDI region. Notably, some national WCBA within high SDI region, such as Estonia and Finland, demonstrated a decreasing trend in the prevalence of depression and DALYs, with percentage changes of approximately −28% and −7%, respectively. This indicates a polarization in the increase of depression cases among national WCBA in high SDI regions. Over the past 32 years, most countries have shown an increasing trend in prevalence and DALYs rates, with Mexico (middle SDI region) reporting the highest increase, with EAPC of 1.73 (95% CI: 1.49–1.97) and 1.91 (95% CI: 1.64–2.17), respectively. Conversely, a small number of countries exhibited decreasing trends in prevalence and DALYs rates, with Singapore (high SDI region) recording the most significant decrease, with EAPC of −1.99 (95% CI: −2.29 to −1.69) and −2.23 (95% CI: −2.56 to −1.91), respectively. Cuba (high-middle SDI region) showed the second-largest decline, with EAPC of −1.36 (95% CI: −1.64 to −1.09) and −1.57 (95% CI: −1.89 to −1.25), respectively (Supplementary Tables S4–S5; Figures 3A,B; Supplementary Figures S4A–S4B).

### Analysis of age patterns

**Figure 4:**
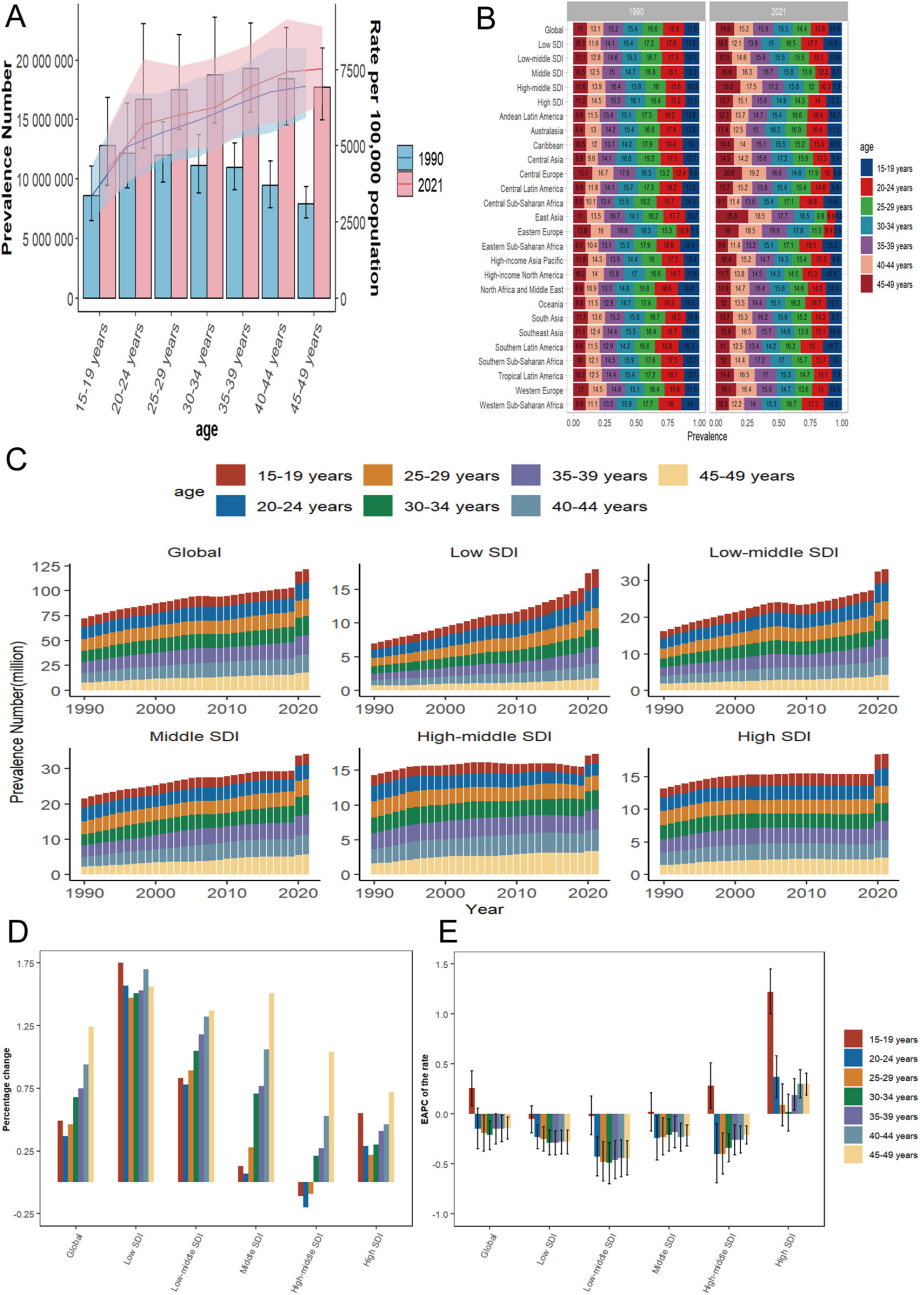
Temporal trends in WCBA depression burden across different age patterns in different regions. **(A)** Global number and rate of prevalent cases in 1990 and 2021. **(B)** Percentage distribution of prevalent cases in 7 age groups in 5 regions and 21 GBD regions globally in 1990 and 2021. **(C)** Prevalent cases of 7 age group (15–49 years, 5-year intervals) from 1990 to 2021 globally and in 5 regions (low to high SDI). **(D)** Percentage change in prevalent cases of 7 age groups globally and in 5 regions in 1990 and 2021. **(E)** EAPC of prevalent rates of 7 age groups globally and in 5 regions from 1990 to 2021. WCBA, women of childbearing age; EAPC, estimated annual percentage change; SDI, socio-demographic index; GBD, global burden of disease.

The number of prevalent cases and prevalence rates, along with the comparison of cases of DALYs and their rates for the seven age groups of WCBA at the global level in 1990 and 2021, are illustrated in (Figure 4A; Supplementary Figure S5). The data shows that the global prevalence of WCBA in 1990 initially increased and then decreased with age, with the peak prevalence observed in the 20–24 age group at 12,186.73 thousand cases (95% UI: 9,264.41–16,382.32 thousand cases), while the lowest prevalence was in the 45–49 age group at 7,932.18 thousand cases (95% UI: 6,739.18–9,375.16 thousand cases). In 2021, the number of prevalent cases of WCBA globally showed a consistent increase with age, with the highest prevalence reaching 19,275.7 thousand cases (95% UI: 15,606.83–23,134.15 thousand) in the 35–39 age group. The trends in DALYs for the global WCBA in 1990 mirrored those of prevalence: the number of DALY cases followed a similar pattern to the prevalence cases, and the DALY rate reflected the prevalence rate trends of that year. Similarly, the trends in DALYs for the global WCBA in 2021 aligned with the trends in prevalent cases and prevalence rates for that year. The detailed trends in the prevalence of depression and the cases of DALYs among WCBA across seven age groups globally, as well as within five SDI regions over the past 32 years, are illustrated in (Figure 4C; Supplementary Figure S7). The percentage change in the number of prevalent cases of depression and DALYs among WCBA globally exhibited a decreasing trend followed by an increasing trend with age, with the smallest percentage changes of 37% and 38% observed in the 20–24 years age group. Conversely, the largest percentage changes of 124% and 126% were recorded in the 45–49 years age group, which is approximately 3.3 times higher than the increase seen in the 20–24 years age group. Furthermore, the trends in the percentage change of depression cases among WCBA varied by age across the five SDI regions. Notably, the percentage change for all age groups in the low SDI region was the highest among the five SDI regions, averaging around 158%. The trends in the prevalence of cases and the percentage change in the number of DALYs across low-middle to high SDI regions in various age groups reflect global trends. However, no significant changes were observed in the low SDI region. Notably, the prevalence and rate of DALYs in the 15–19 age group within the high SDI region increased at a rate surpassing the global average, with an EAPC of 1.22 (95% CI: 1–1.45) and 1.35 (95% CI: 1.1–1.59), respectively (Table 2; Supplementary Table S6; Figure 4E; Supplementary Figure S9). Among the 21 regions analyzed, East Asia exhibited the most significant increase in the proportion of depression cases among WCBA within the 45–49 age group. The prevalence of depression among WCBA rose from 11% in 1990 to 25.8% in 2021, while the proportion of DALYs attributed to depression increased from 9.7% to 25%. Conversely, the most substantial decrease in the proportion of cases in this region was noted in the 15–19 age group. This indicates a notable trend of shifting depression prevalence among WCBA from younger to older age groups (Figure 4B; Supplementary Figure S6).

**Table 2:** The prevalence of depression cases and rates among WCBA in 1990 and 2021, and the trends in age patterns from 1990 to 2021.

Location	Age (years)	Prevalence cases			Prevalence rates		
		1990_thousands (95% UI)	2021_thousands (95% UI)	Percentagechange (100%)	1990_per 100 000 (95% UI)	2021_per 100 000 (95% UI)	EAPC(95% CI)
Global	15–19	8,599.33 (6,529.06–11,098.07)	12,840.7 (9,489.52–16,836.97)	0.49	3,365.18 (2,555.02–4,343.01)	4,228.78 (3,125.15–5,544.86)	0.26 (0.08–0.43)
	20–24	12,186.73 (9,264.41–16,382.32)	16,718.17 (12,607.12–23,077.96)	0.37	4,991.8 (3,794.79–6,710.35)	5,691.25 (4,291.75–7,856.27)	−0.15 (−0.35–0.06)
	25–29	12,010.97 (9,765.94–14,816.69)	17,500.01 (14,180.52–22,147.97)	0.46	5,457.06 (4,437.05–6,731.81)	6,014.01 (4,873.24–7,611.32)	−0.19 (−0.37–0.01)
	30–34	11,130.37 (8,817.95–13,741.86)	18,739.69 (14,686–23,575.26)	0.68	5,854.73 (4,638.37–7,228.41)	6,268.9 (4,912.84–7,886.52)	−0.21 (−0.36 to 0.07)
	35–39	10,993.55 (9,079.52–13,008.17)	19,275.72 (15,606.83–23,134.15)	0.75	6,338.04 (5,234.56–7,499.52)	6,938.64 (5,617.95–8,327.55)	−0.15 (−0.3 to 0)
	40–44	9,492.7 (7,595.9–11,510.35)	18,420.97 (14,521.67–22,726.53)	0.94	6,769.6 (5,416.92–8,208.46)	7,425.12 (5,853.39–9,160.6)	−0.15 (−0.28–0.01)
	45–49	7,932.18 (6,739.18–9,375.16)	17,742.68 (14,956.26–21,023.65)	1.24	6,970.24 (5,921.91–8,238.23)	7,529.49 (6,347.01–8,921.84)	−0.14 (−0.25–0.03)
	15–49	72,345.83 (63,629.46–84,065.84)	121,237.94 (104,691.69–142,427.51)	0.68	5,409.59 (4,757.83–6,285.95)	6,220.98 (5,371.96–7,308.26)	−0.02 (−0.1 7–0.13)
Low SDI	15–19	941.33 (687.82–1,253.55)	2,589.29 (1,847.2–3,448.88)	1.75	3,746.02 (2,737.17–4,988.48)	4,199.42 (2,995.87–5,593.55)	−0.05 (−0.19 to 0.08)
	20–24	1,237.3 (922.49–1,706.86)	3,177.92 (2,344.12–4,386.63)	1.57	5,696.37 (4,247.02–7,858.17)	6,026.51 (4,445.33–8,318.68)	−0.23 (−0.35 to 0.11)
	25–29	1,198.15 (949.72–1,537.1)	2,963.54 (2,287.67–3,797.94)	1.47	6,468.31 (5,127.15–8,298.14)	6,727.83 (5,193.45–8,622.08)	−0.25 (−0.37 to 0.13)
	30–34	1,071.47 (819.43–1,358.77)	2,691.88 (2,042.92–3,438.49)	1.51	7,044.09 (5,387.09–8,932.85)	7,252.14 (5,503.79–9,263.56)	−0.29 (−0.41 to 0.16)
	35–39	980.99 (783.55–1,206.89)	2,485.85 (1,950.55–3,049.18)	1.53	7,613.67 (6,081.3–9,366.87)	7,801 (6,121.15–9,568.84)	−0.29 (−0.41 to 0.17)
	40–44	804.77 (612.03–1,017.72)	2,175.72 (1,652.08–2,740.33)	1.70	8,116.51 (6,172.66–10,264.3)	8,329.74 (6,324.98–10,491.32)	−0.28 (−0.4 to 0.16)
	45–49	713.49 (581.37–859.96)	1,826.48 (1,487.34–2,205.84)	1.56	8,594.49 (7,003–10,358.88)	8,794.94 (7,161.91–10,621.61)	−0.28 (−0.4–0.16)
	15–49	6,947.49 (5,923.11–8,385.6)	17,910.68 (15,152.15–21,726)	1.58	6,220.59 (5,303.39–7,508.24)	6,529.33 (5,523.71–7,920.21)	−0.22 (−0.34 to 0.1)
Low-middle SDI	15–19	1,963.11 (1,444.95–2,589.89)	3,586.93 (2,621.82–4,722.8)	0.83	3,341.36 (2,459.41–4,408.19)	3,967.8 (2,900.21–5,224.27)	−0.02 (−0.21 to 0.18)
	20–24	2,801.46 (2,089.27–3,794.46)	4,980.15 (3,739–6,957.18)	0.78	5,371.47 (4,005.93–7,275.43)	5,723.72 (4,297.26–7,995.93)	−0.43 (−0.62 to 0.23)
	25–29	2,712.37 (2,149.85–3,419.08)	5,126.95 (4,071.1–6,649.94)	0.89	6,043.22 (4,789.93–7,617.81)	6,310.33 (5,010.78–8,184.84)	−0.48 (−0.67 to 0.28)
	30–34	2,489.31 (1,934.68–3,114.15)	5,111.06 (3,974.4–6,453.25)	1.05	6,648.13 (5,166.89–8,316.89)	6,915.44 (5,377.5–8,731.47)	−0.49 (−0.7 to 0.29)
	35–39	2,369.02 (1,904.55–2,873.98)	5,158.97 (4,137.02–6,311.42)	1.18	7,395.31 (5,945.38–8,971.62)	7,747.74 (6,212.97–9,478.47)	−0.46 (−0.65 to 0.27)
	40–44	2,074.42 (1,603.55–2,590.02)	4,821.87 (3,735–6,077.13)	1.32	7,995.6 (6,180.68–9,982.93)	8,359.44 (6,475.18–10,535.62)	−0.44 (−0.63 to 0.26)
	45–49	1,807.39 (1,500.69–2,140.83)	4,276.36 (3,534.58–5,147.11)	1.37	8,327.03 (6,913.97–9,863.24)	8,650.11 (7,149.66–10,411.44)	−0.44 (−0.61 to 0.27)
	15–49	16,217.07 (14,010.59–19,217.57)	33,062.3 (28,240.33–39,580.27)	1.04	5,942.14 (5,133.65–7,041.56)	6,530.56 (5,578.11–7,818)	−0.29 (−0.48 to 0.1)
Middle SDI	15–19	2,661.2 (2,016.73–3,429.71)	2,996.6 (2,228.33–3,958.64)	0.13	2,886.2 (2,187.24–3,719.69)	3,411.5 (2,536.86–4,506.75)	0.02 (−0.17 to 0.21)
	20–24	3,897.76 (2,939.78–5,208.52)	4,167.32 (3,133.95–5,719.25)	0.07	4,412.62 (3,328.1–5,896.52)	4,808.39 (3,616.06–6,599.07	−0.24 (−0.46 to 0.02)
	25–29	3,632.91 (2,937.55–4,460.99)	4,667.26 (3,730.72–5,825.03)	0.28	4,849.84 (3,921.55–5,955.3)	5,151.58 (4,117.86–6,429.5)	−0.23 (−0.41 to 0.04)
	30–34	3,171.73 (2,519.31–3,881.75)	5,417.3 (4,329.19–6,714.34)	0.71	5,282.78 (4,196.12–6,465.39)	5,485.7 (4,383.84–6,799.12)	−0.21 (−0.37 to 0.05)
	35–39	3,243.87 (2,678.1–3,829.66)	5,735.88 (4,680.76–6,820.36)	0.77	5,852.03 (4,831.37–6,908.81)	6,258.62 (5,107.34–7,441.94)	−0.18 (−0.34 to 0.02)
	40–44	2,705.74 (2,163.6–3,290.22)	5,570.73 (4,394.06–6,843.39)	1.06	6,403.2 (5,120.2–7,786.38)	6,805.96 (5,368.38–8,360.81)	−0.23 (−0.37 to 0.09)
	45–49	2,263.9 (1,925.92–2,682.12)	5,692.92 (4,797.07–6,698.95)	1.51	6,679.54 (5,682.35–7,913.5)	7,018.66 (5,914.2–8,258.99)	−0.22 (−0.32 to 0.11)
	15–49	21,577.11 (18,934.36–25,064.37)	34,248 (29,807.36–39,826.86)	0.59	4,826.4 (4,235.27–5,606.44)	5,537.56 (4,819.56–6,439.61)	0.03 (−0.13 to 0.18)
High-middle SDI	15–19	1,552.69 (1,199.65–1,968.7)	1,374.36 (986.24–1,824.56)	−0.11	3,278.45 (2,533.02–4,156.86)	3,991.28 (2,864.15–5,298.69)	0.28 (0.06–0.51)
	20–24	2,227.99 (1,703.45–2,940.56)	1,783.56 (1,310.16–2,451.01)	−0.20	4,628.06 (3,538.46–6,108.23)	5,010.6 (3,680.68–6,885.69)	−0.4 (−0.69–0.1)
	25–29	2,291.49 (1,871.37–2,790.73)	2,078.08 (1,660.47–2,638.85)	−0.09	5,009.19 (4,090.8–6,100.53)	5,155.36 (4,119.34–6,546.54)	−0.4 (−0.6 to 0.19)
	30–34	2,254.01 (1,799.32–2,742.21)	2,738.53 (2,183.99–3,414.17)	0.21	5,399.04 (4,309.92–6,568.42)	5,318.49 (4,241.51–6,630.65)	−0.34 (−0.48 to 0.2)
	35–39	2,341.31 (1,951.25–2,749.68)	2,981.75 (2,407.15–3,571.05)	0.27	5,927.84 (4,940.27–6,961.76)	6,017.54 (4,857.94–7,206.84)	−0.26 (−0.41 to 0.11)
	40–44	1,984.34 (1,587.49–2,400.57)	3,036.72 (2,406.84–3,773.87)	0.53	6,454.34 (5,163.55–7,808.2)	6,671.28 (5,287.51–8,290.7)	−0.26 (−0.39 to 0.12)
	45–49	1,657.16 (1,414.14–1,948.15)	3,382.91 (2,846.11–4,005.19)	1.04	6,759.12 (5,767.89–7,946)	7,014.42 (5,901.36–8,304.7)	−0.21 (−0.3 to 0.12)
	15–49	14,308.99 (12,638.28–16,523.37)	17,375.91 (15,039.61–20,513.34)	0.21	5,151.68 (4,550.17–5,948.93)	5,694.63 (4,928.95–6,722.86)	−0.06 (−0.22 to 0.1)
High SDI	15–19	1,473.69 (1,141.94–1,852.61)	2,284.17 (1,760.64–2,863.46)	0.55	4,624.21 (3,583.23–5,813.2)	7,851.58 (6,052–9,842.84)	1.22 (1–1.45)
	20–24	2,012.09 (1,584.83–2,619.94)	2,596.8 (1,957.64–3,583.08)	0.29	5,992.24 (4,719.82–7,802.5)	8,237.62 (6,210.07–11,366.33)	0.37 (0.16–0.58)
	25–29	2,166.17 (1,791.75–2,555.06)	2,651.35 (2,147.43–3,283.98)	0.22	6,042.99 (4,998.45–7,127.88)	7,671.92 (6,213.78–9,502.5)	0.09 (−0.12 to 0.3)
	30–34	2,134.41 (1,757.03–2,542.83)	2,767.46 (2,162.5–3,447.64)	0.30	6,015.04 (4,951.52–7,166.02)	7,393.05 (5,776.95–9,210.1)	0.02 (−0.17 to 0.2)
	35–39	2,049.1 (1,757.11–2,368.09)	2,899.41 (2,433.26–3,430.75)	0.41	6,127.83 (5,254.65–7,081.8)	7,643.65 (6,414.73–9,044.41)	0.19 (0.04–0.35)
	40–44	1,914.92 (1,609.65–2,282.25)	2,802.15 (2,261.19–3,383.33)	0.46	6,133.02 (5,155.32–7,309.49)	7,632.89 (6,159.34–9,215.97)	0.3 (0.16–0.44)
	45–49	1,482.73 (1,274.55–1,729.31)	2,550.51 (2,161.27–2,985.68)	0.72	5,868.32 (5,044.41–6,844.24)	7,103.29 (6,019.24–8,315.25)	0.3 (0.19–0.41)
	15–49	13,233.1 (11,695.2–14,990.19)	18,551.85 (16,170.07–21,444.5)	0.40	5,837.1 (5,158.74–6,612.15)	7,629.55 (6,650.03–8,819.17)	0.33 (0.16–0.49)

WCBA, women of childbearing age; EAPC, estimated annual percentage change; CI, confidence intervals; UI, uncertainty intervals; SDI, socio-demographic index.

### Proportion of DALYs attributable to risk factors (regional age-differentiated analysis)

**Figure 5:**
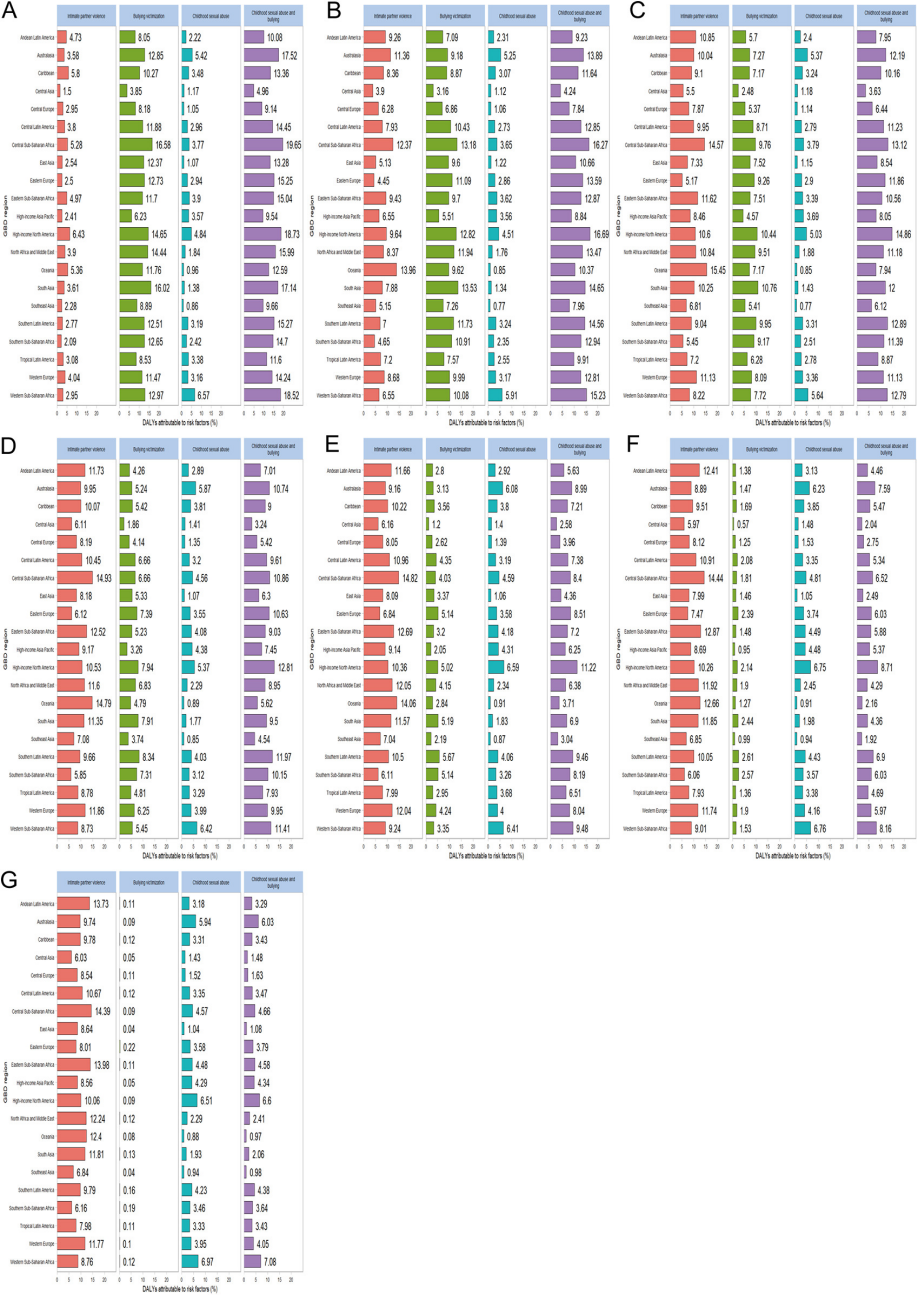
Regional differences in the distribution of major risk factors for WCBA depression DALYs across different age groups **(A)** represents 15–19 years old, **(B)** represents 20–24 years old, **(C)** represents 25–29 years old, **(D)** represents 30–34 years old, **(E)** represents 35–39 years old, **(F)** represents 40–44 years old, **(G)** represents 45–49 years old. WCBA, women of childbearing age.

A comprehensive analysis conducted in 2021 examined four significant risk factors associated with depression among WCBA across various age groups. These factors included intimate partner violence, experiences of bullying victimization, instances of childhood sexual abuse, and the combined impact of childhood sexual abuse and bullying. Across the 21 regions studied, childhood sexual abuse and bullying were identified as having the most detrimental effects on young women aged 15–19 years, while intimate partner violence was associated with the least harm within this age group. In contrast, for middle-aged women aged 45–49 years, intimate partner violence resulted in the most significant harm, whereas childhood sexual abuse and bullying were linked to the least harm. This trend starkly contrasts with that observed in adolescent females. It is noteworthy that WCBA experience intimate partner violence persistently over time, and the cumulative effects of physical violence can ultimately lead to depression. Specifically, WCBA aged 15−19 years accounted for 19.65% of DALYs due to childhood sexual abuse and bullying in Central Saharan Africa, while those aged 45–49 years accounted for 13.73% of DALYs due to intimate partner violence in the Andean region of Latin America. In the high-income Asia-Pacific and Central Asia regions, the impact of these risk factors was relatively low; however, they were more pronounced in the central and eastern regions of South Asia and Saharan Africa (Figure 5).

### Age, period and birth cohort

**Figure 6:**
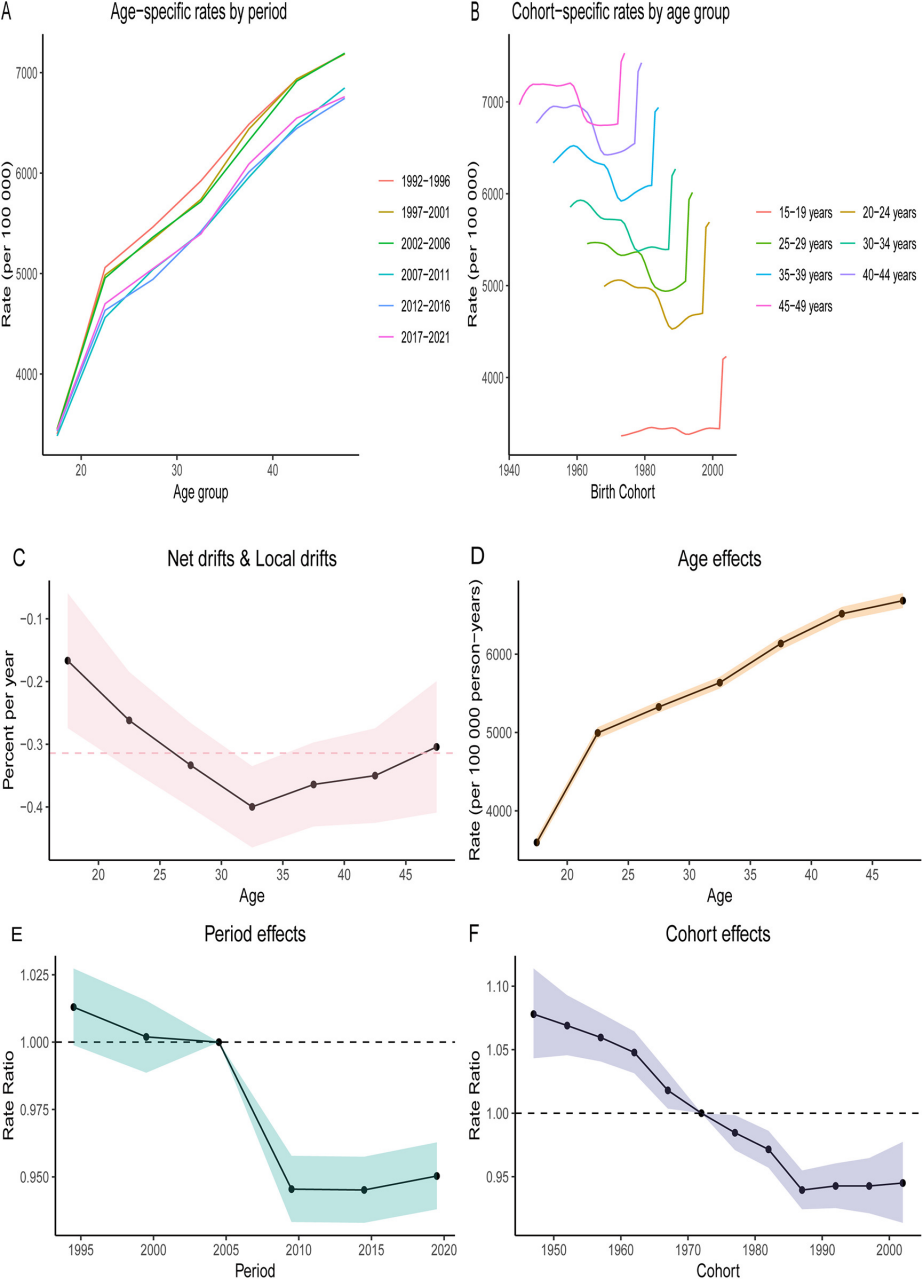
Age-period-cohort (APC) analysis on the prevalence of depression among different age groups (15–19, 20–24, 25–29, 30–34, 35–39, 40–44, 45–49 years old) across global quintiles. The age distribution of prevalence illustrates the temporal changes in the relative proportions of prevalence among different age groups from 1990 to 2021. **(A)** Age-specific rates by period. **(B)** Cohort-specific rates by age group. **(C)** Local drift indicates the annual percentage change (percentage per year) in specific prevalence for each age group from 1990 to 2021. **(D)** The age effect is represented by the fitted longitudinal age-specific prevalence after adjusting for period bias for a specific number of birth cohorts. **(E)** The period effect is illustrated by the period relative risk of prevalence (prevalence ratio), calculated as the ratio of age specific prevalence from the period 1992–1996 to that of 2017–2021. **(F)** The birth cohort effect is indicated by the cohort relative risk of prevalence (prevalence ratio), calculated as the ratio of age-specific prevalence from the 1947 cohort to that of the 2002 cohort. Points and shaded areas represent the prevalence or ratios and their corresponding 95% confidence intervals.

Figure 6; Supplementary Figure S10 illustrate the dynamics of age, period, and cohort concerning the prevalence and DALYs rates for depression among WCBA across different age groups globally. Overall, both the prevalence and DALYs rates of depression increased with age, with higher rates observed in older age groups. However, the Age-Standardized Rate (ASR) exhibited a more gradual increase with advancing age.

Globally, the prevalence of WCBA depression and DALYs rates across various age groups demonstrated a decreasing trend from 2005 to 2010, followed by a stabilization in recent years. Regarding birth cohorts, the prevalence and DALYs rates were generally higher among younger generations, indicating the influence of age on depression. Notably, however, there has been a rapid increase in prevalence and DALYs rates across all age groups since 1990, particularly among the younger cohort aged 15–29 years.

### Global prevalence and DALYs rates of WCBA depression in different age groups projected to 2040

**Figure 7:**
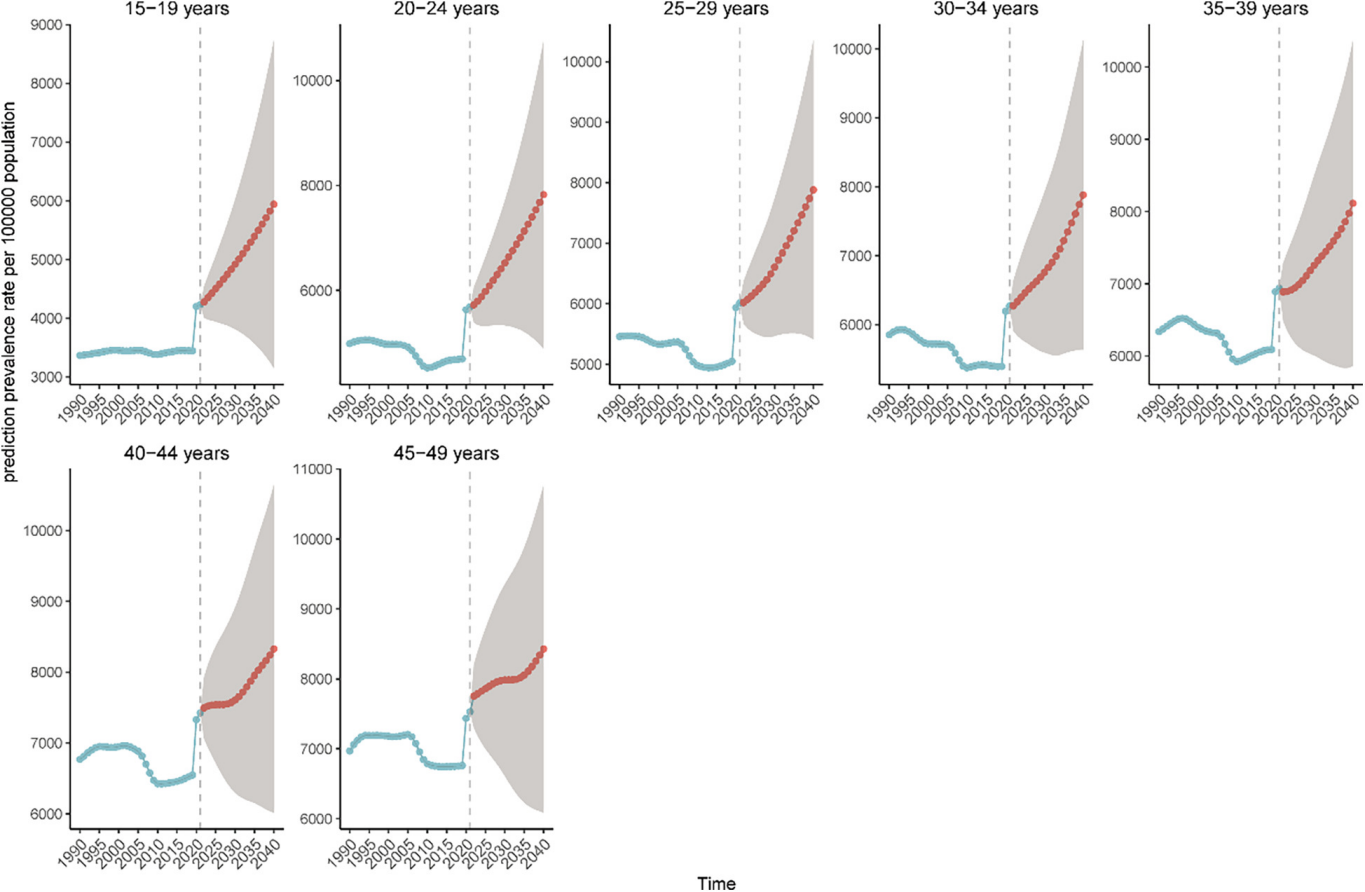
Projected trends in the prevalence of depression in the WCBA from 2021 to 2040. The blue line indicates the true trend in prevalence, while the red line indicates the predicted trend.

Globally, the prevalence of WCBA and DALYs across various age groups is projected to increase between 2022 and 2040. The most significant rise in prevalence and DALYs rates is observed among women aged 15–29 years, which may be attributed to the evolving social roles of young women. Conversely, the growth of prevalence and DALYs rates among middle-aged women aged 40–49 years exhibited a notable slowdown from 2022 to 2030, followed by a rapid increase from 2030 to 2040. This trend may be linked to middle-aged women transitioning into older age, often accompanied by adverse life events, such as widowhood, which can lead to increased social isolation (Figure 7; Supplementary Figure S11, Supplementary Tables S7–S8).

## Discussion

In 2015, the United Nations introduced Sustainable Development Goal 3 (SDG 3), which aims to “promote well-being and ensure healthy lifestyles for individuals of all ages.” This goal emphasizes the necessity of reducing the global burden of non-communicable diseases (29). Depression is one of the leading contributors to disability among WCBA and poses a significant barrier to achieving this objective. Our analysis, informed by data from the Global Burden of Disease Study 2021, reveals critical trends in the prevalence of depression and DALYs for females aged 15–49 from 1990 to 2021, highlighting the urgent need for targeted interventions.

Worldwide, the impact of depression on women of reproductive age has significantly increased over the past 32 years. The prevalence of cases in 2021 was 68% higher compared to 1990. Similarly, there has been a 69% increase in DALYs. Although there has been a slight reduction in the EAPC, the rising rates of prevalence and DALYs suggest a prolonged duration of illness, inadequate access to effective treatments, and the expansion of the population base, collectively driving the increase in absolute case numbers, while the EAPC remains stable (30–32). Through Joinpoint regression analysis, we found that the AAPC for global WCBA depression prevalence was 0.4789 (95% CI: 0.3289–0.6295; *P* < 0.001). Additionally, the APC for prevalence during the period from 2019 to 2021 was 8.7824 (95% CI: 6.7558–10.8476; *P* < 0.001), which may be attributed to the increased depression levels among WCBA resulting from the COVID−19 pandemic (33). A comprehensive mental health study conducted among pregnant and postpartum women across 64 different countries during the COVID−19 pandemic revealed alarming statistics: 43% of participants exhibited elevated levels of post-traumatic stress disorder (PTSD) symptoms related to COVID-19, while 31% surpassed thresholds indicative of depression and anxiety. These figures highlight the profound psychological toll that the pandemic has had on this vulnerable population. Moreover, subsequent research has indicated a troubling correlation between the frequency of information-seeking behaviors and mental health outcomes. Specifically, women who sought information related to the pandemic more than five times a day through any medium or type of media experienced over a twofold increase in the risk of developing heightened symptoms of PTSD, depression, and anxiety. This suggests that excessive exposure to information, particularly in a crisis, may exacerbate mental health challenges rather than alleviate them, underscoring the need for balanced information consumption during periods of uncertainty. The excessive pursuit of information regarding the COVID-19 outbreak has the potential to exacerbate negative emotions among women, ultimately contributing to mental health issues. The constant influx of distressing news can create a cycle of anxiety and worry, further impacting emotional well-being (34–36). Additionally, research conducted by Stevens and Hamann (50) through a meta-analysis of imaging studies indicates that women exhibit greater activation in the left amygdala and other related brain areas when confronted with negative emotions compared to men. This finding suggests that women may experience a more pronounced response to negative emotional stimuli, which correlates with a higher prevalence of depression among them (37).

The impact of depression is notably more significant in the five regions classified by the SDI. Notably, the percentage change for all age groups in the low SDI region was the highest among the five SDI regions, averaging around 158%. This region experienced a rapid increase in the number of cases despite a negative EAPC, likely attributable to the significant burden of early childbearing prevalent in low SDI regions (38). Among these regions, those with higher SDI scores exhibit the highest prevalence rates of depression as well as the greatest number of DALYs lost due to the condition. This trend is particularly striking given that these high SDI regions typically possess advanced healthcare systems. A possible explanation for this phenomenon lies in the fact that developed countries often prioritize early screening and interventions, leading to increased diagnosis rates. Consequently, while the healthcare infrastructure may be robust, it also results in a greater identification of depressive disorders, reflecting an alarming prevalence that demands attention (39). The significant rise in depression rates among WCBA in high SDI region from 2018 to 2021 indicates a decline in their mental health during the COVID-19 pandemic. This deterioration can be attributed to several factors, particularly prolonged periods of home isolation, reduced opportunities for face-to-face social interactions, and the accompanying economic crisis. These abrupt environmental shifts have critically impacted the mental well-being of WCBA, underscoring the urgent need for targeted mental health interventions during such challenging times (40). These factors suggest that the challenges faced by low SDI region may hinder not only access to medical care but also the recognition and identification of health issues within these communities (41). Significant geographic disparities are apparent, particularly noted by the marked rise in the prevalence of WCBA depression and DALYs rates in affluent regions of North America. This trend may be attributed to the impact of socioeconomic factors and ethnic backgrounds on women's mental health (42). Countries that are rich in energy resources, such as Qatar and the United Arab Emirates, have witnessed a staggering rise in the prevalence of health issues, alongside an equally significant increase in DALYs, ranging from about 480% to 630% since 1990. This alarming trend highlights the challenges faced by immigrant women in these nations, who often experience inadequate social support systems. The stressful conditions of their daily lives, combined with limited access to health care services, contribute significantly to their deteriorating health outcomes. These factors underline the need for comprehensive strategies that address the unique challenges faced by immigrant populations in energy-rich countries (43). In a notable departure from the trend observed in other regions, Georgia and Latvia demonstrated a significant decrease in mental health issues, approximating a 30% reduction. This substantial decline serves as a testament to the effective integration of mental health care services within the realm of primary health care. This integration indicates that both countries have made strides in addressing mental health needs by incorporating them into more accessible health care frameworks, thereby enhancing the overall well-being of their populations. The successful collaboration between mental health initiatives and primary health care systems reflects a progressive approach to health care that prioritizes comprehensive mental health strategies.

The prevalence of depression among WCBA demonstrates a notable correlation with age, revealing that as women grow older, their susceptibility to this mental health condition significantly rises. Specifically, data indicates a staggering 124% increase in cases of depression within the 45–49 years age group worldwide from 1990 to 2021. This rise can be attributed to several interrelated factors, including menopausal symptoms that commonly emerge during this stage of life (44), as well as sleep disorders that often accompany these changes. Moreover, biopsychosocial factors—encompassing physical, emotional, and social influences—along with dynamics related to partners further contribute to the heightened risk of developing depression among middle-aged women. These findings underscore the importance of addressing the unique challenges faced by this demographic in order to mitigate the burden of depression and promote better mental health outcomes (45). In high SDI region, there has been a notable increase in prevalence rates among individuals aged 15–19, marking the most rapid growth in this particular age group. Furthermore, the group aged 20–24 exhibits the highest prevalence overall. This trend may be attributed to a combination of physiological and psychological transformations that take place during adolescence, particularly among females. Additionally, adolescent females may experience increased vulnerability to psychological stress, which could further contribute to the rising prevalence in these age brackets (46).

Research has highlighted the significant impact of childhood trauma on the development of psychiatric disorders later in life. This association is particularly strong in cases where individuals have experienced bullying, emotional abuse, and childhood sexual abuse. Adolescents who endure multiple types of maltreatment are especially vulnerable; the data indicates that they are more than three times more likely to face psychiatric disorders compared to those who have not experienced such traumas. This underscores the critical need for early intervention and support for young individuals who have faced various forms of abuse and maltreatment, as early experiences can have profound implications for their mental health in adulthood (47). Nurses in economically underdeveloped countries mostly lack pre-service training in IPV, CSA, and BV, making it difficult for them to effectively alleviate the conditions of such patients (48). We strongly advocate for the implementation of social protection measures aimed at enhancing the empowerment of women in several critical areas. This includes fostering their involvement in decision-making processes, as well as ensuring their autonomy in education and employment. Additionally, it is crucial to raise community awareness regarding the serious consequences of depression among WCBA. By addressing these factors comprehensively, we can promote mental well-being and support the overall empowerment of women within the community (49).

In conclusion, this study highlights significant shifts in the prevalence of depression among WCBA, as well as the associated DALYs on a global, regional, and national scale over the past 32 years. Importantly, it offers projections regarding the global burden of WCBA depression across various age groups, extending to the year 2040. These findings underscore the necessity for countries to recognize and address the increasing challenges posed by mental illness, which are expected to intensify in the coming years. Moreover, this research establishes a robust scientific foundation that can inform the development of effective strategies for the prevention of depression. It also offers valuable recommendations for the treatment of mental health issues, thereby equipping policymakers and healthcare providers with the insights needed to improve outcomes for those affected. Between 1991 and 2021, three versions of DSM (IV, IV-TR, 5) and two versions of ICD (10, 11) were successively introduced as diagnostic tools for depression. These versions refined subtypes of depression (such as seasonal depression), expanded the scope of diagnosis, and improved the annotation system. The imperative of addressing these mental health concerns is clearer than ever, and proactive measures are essential to mitigate the anticipated future burden of mental illness.

This study has several limitations. The estimation of disease burden in GBD 2021 heavily relies on the sources and quality of data. Despite the use of the advanced DisMod-MR 2.1 model to integrate data heterogeneity, significant disparities persist across countries in terms of coverage, reporting systems, diagnostic criteria, and data collection cycles, presenting ongoing challenges. Firstly, Cultural differences lead to variations in the clinical manifestations of depression. In low- and middle-income countries, patients often present with physical discomfort or culturally specific forms of distress (such as persistent fatigue, insomnia, and pain), which do not align with the typical psychological symptoms seen in Western contexts, such as self-blame and feelings of worthlessness. If diagnostic criteria do not match, cases are easily missed, leading to underestimation. Conversely, if diagnostic tools are widely available and medicalization is high, there may be over-identification and overestimation of cases. These differences introduce systematic biases in comprehensive analyses, thereby affecting the overall estimation of global or regional disease burden. Secondly, although GBD data integrates multiple sources of information such as health records, population surveys, scientific literature, and hospital registries, its quality varies depending on the level of investment in different countries and regions. In low SDI countries, systematic mental health registries are often absent, and research has to rely more on statistical imputation models, which directly undermines the accuracy and reliability of the estimates. Meanwhile, this study did not adjust for key variables such as education, income, employment, marital status, immigration, structural violence, and urbanization, which precisely determine the accessibility of mental health services and profoundly influence the prevalence and diagnostic risk of diseases. The absence of these factors may introduce uncontrollable biases in regional comparisons. Finally, while BAPC can provide short- to medium-term trend predictions in the GBD, its accuracy is constrained by data gaps, model assumptions, and insufficient coverage of external factors, particularly requiring cautious interpretation in low- and middle-income countries.

## Conclusion

Between 1990 and 2021, there has been a notable global rise in both the prevalence and DALY rates associated with depression, along with an increase in the total cases among WCBA. This trend underlines the pressing necessity to prioritize the mental health of this demographic. Conversely, East Asia demonstrates a declining trend in the prevalence, DALY rates, and the overall number of depression cases, indicating that this region is placing greater emphasis on the psychosocial well-being of WCBA and the improvement of women's societal status. Furthermore, it is anticipated that by the year 2040, there will be a notable rise in the global prevalence of WCBA, alongside an increase in the rate of DALYs across various age groups. This projection suggests that as the population of WCBA grows, we may also see a corresponding rise in health-related burdens, indicating the need for heightened awareness and proactive measures in public health planning and resource allocation. We anticipate that the research findings presented in this article will serve as a valuable reference for national policymakers as they seek to design and implement targeted prevention and control strategies for the disease. All measures must be tailored to local conditions, especially in low SDI countries, where efforts should be focused on enhancing women's status, alleviating family economic pressures, and improving postpartum care systems. By taking into account the unique challenges and needs of these countries, policymakers can enhance the effectiveness of their public health initiatives and ensure a more successful response to the disease.

## Data Availability

The original contributions presented in the study are included in the article/Supplementary Material, further inquiries can be directed to the corresponding author.
