# Supplementary material for: Global, regional, and national trends in depressive disorder prevalence and DALYs among women of childbearing age from 1990 to 2021 and projections to 2040: a comprehensive analysis from 1990 to 2021
Source: Front Glob Womens Health. 2025 Aug 18;6:1629747. doi: 10.3389/fgwh.2025.1629747 (PMC12399658; doi:10.3389/fgwh.2025.1629747)
Supplement: Supplementary file 1 [file Datasheet1.docx]

**Global, regional, and national trends in depressive disorder prevalence and DALYs among women of childbearing age from 1990 to 2021 and projections to 2040: a comprehensive analysis from 1990 to 2021**

Yuhang Yang^1*^, Yuyuan Hu^1*^, Yuan He^2*^, Wei Zhang^1^, Jinghan Jia^1^, Yibo Xu^3^, Yan Li^4^, Jinxi Wang^1+^

**Affiliations:**

1 Division of Colorectal Surgery, Third Hospital of Shanxi Medical University, Shanxi Bethune Hospital, Shanxi Academy of Medical Sciences Tongji Shanxi Hospital, Taiyuan, 030032, China.

2 Hepatobiliary surgery, Baogang Hospital of InnerMongolia, Baotou, 014000, China.

3 Xi’an Medical University, Xi’an,710021,China

4 Neurology, Third Hospital of Shanxi Medical University, Shanxi Bethune Hospital, Shanxi Academy of Medical Sciences Tongji Shanxi Hospital, Taiyuan, 030032, China.

+Correspondence: Jinxi Wang, E-mail: tywjx163@sxmu.edu.cn.

*These authors contributed equally to this work

**Contents**

[**Global, regional, and national trends in depressive disorder prevalence and DALYs among women of childbearing age from 1990 to 2021 and projections to 2040: a comprehensive analysis from 1990 to 2021** 1](#_Toc204161232)

[**Table S1 The DALYs of depression cases and rates among WCBA (15-49 years) in 1990 and 2021, and the trends from 1990 to 2021.** 2](#_Toc204161233)

[**Table S2. Temporal Joinpoint analysis of depression prevalence rates in WCBA, 1990-2021** 5](#_Toc204161234)

[**Table S3. Temporal Joinpoint Analysis of Depression DALYs Rates in WCBA, 1990-2021** 10](#_Toc204161235)

[**Table S4. The prevalence of depression cases and rates among WCBA in 1990 and 2021 across 204 countries, and the trends from 1990 to 2021.** 15](#_Toc204161236)

[**Table S5. The DALYs of depression cases and rates among WCBA in 1990 and 2021 across 204 countries, and the trends from 1990 to 2021.** 32](#_Toc204161237)

[**Table S6. The DALYs of depression cases and rates among WCBA in 1990 and 2021, and the trends in age patterns from 1990 to 2021.** 48](#_Toc204161238)

[**Table S7. Projection of depression prevalence rates among seven age groups of WCBA(15-49 Years) from 2022 to 2040.** 52](#_Toc204161239)

[**Table S8. Projection of depression DALYs rates among seven age groups of WCBA (15-49 Years) from 2022 to 2040.** 57](#_Toc204161240)

[**FigureS1-S3.** 64](#_Toc204161241)

[**FigureS4.** 65](#_Toc204161242)

[**FigureS5-S9.** 67](#_Toc204161243)

[**FigureS10.** 69](#_Toc204161244)

[**FigureS11.** 71](#_Toc204161245)

# **Table S1 The DALYs of depression cases and rates among WCBA (15-49 years) in 1990 and 2021, and the trends from 1990 to 2021.**

| location | DALY cases | | | | | | | DALY rates | | | | |
| --- | --- | --- | --- | --- | --- | --- | --- | --- | --- | --- | --- | --- |
|  | 1990_millions(95% UI) | | 2021_millions(95% UI) | | percentage change(100%) | | | 1990_per 100 000(95% UI) | | 2021_per 100 000(95% UI) | | EAPC(95% CI) |
| Global | 12.45 (8.43-17.07) | | 21.04 (14.07-29.06) | | 0.69 | | | 930.59 (630.46-1276.48) | | 1079.73 (722.08-1491.21) | | -0.06 (-0.24-0.12) |
| Low SDI | 1.18 (0.79-1.66) | | 3.1 (2.05-4.32) | | 1.63 | | | 1059.98 (708.71-1483.53) | | 1130.08 (748.96-1576.53) | | -0.24 (-0.38--0.1) |
| Low-middle SDI | 2.83 (1.88-3.93) | | 5.82 (3.88-8.06) | | 1.06 | | | 1037.85 (687.9-1441.62) | | 1149.02 (766.03-1592.11) | | -0.37 (-0.59--0.15) |
| Middle SDI | 3.68 (2.48-5.05) | | 5.82 (3.88-7.95) | | 0.58 | | | 822.53 (554.25-1130.53) | | 941.03 (626.82-1284.75) | | -0.05 (-0.24-0.14) |
| High-middle SDI | 2.45 (1.66-3.36) | | 2.94 (1.97-4.08) | | 0.2 | | | 881.83 (597.9-1211.3) | | 963.27 (646.17-1335.82) | | -0.15 (-0.34-0.05) |
| High SDI | 2.29 (1.59-3.12) | | 3.35 (2.33-4.61) | | 0.46 | | | 1010.91 (702.93-1375.53) | | 1377.95 (956.5-1895.89) | | 0.4 (0.21-0.59) |
| Andean Latin America | 0.07 (0.05-0.1) | | 0.17 (0.11-0.24) | | 1.43 | | | 728.84 (482.31-1023.34) | | 970.01 (610.5-1379.98) | | 0.16 (-0.18-0.5) |
| Australasia | 0.07 (0.05-0.1) | | 0.11 (0.07-0.16) | | 0.57 | | | 1377.61 (946.98-1870.68) | | 1536.23 (1018.03-2201.02) | | 0.19 (0.04-0.33) |
| Caribbean | 0.11 (0.07-0.15) | | 0.14 (0.09-0.21) | | 0.27 | | | 1142.4 (751.92-1605.92) | | 1201.97 (776.64-1713.29) | | -0.41 (-0.64--0.18) |
| Central Asia | 0.13 (0.08-0.18) | | 0.22 (0.14-0.32) | | 0.69 | | | 749.27 (498.06-1045.6) | | 910.07 (596.5-1303.44) | | 0.23 (0.07-0.39) |
| Central Europe | 0.22 (0.15-0.3) | | 0.21 (0.14-0.29) | | -0.05 | | | 703 (477.98-969.12) | | 819.58 (540.88-1138.53) | | -0.25 (-0.51-0.01) |
| Central Latin America | 0.32 (0.21-0.44) | | 0.8 (0.52-1.11) | | 1.5 | | | 754.83 (503.23-1059.91) | | 1173.59 (764.69-1628.9) | | 1.07 (0.87-1.26) |
| Central Sub-Saharan Africa | 0.19 (0.12-0.27) | | 0.54 (0.34-0.76) | | 1.84 | | | 1538.76 (1006.73-2214.57) | | 1640.54 (1056.02-2327.58) | | 0.02 (-0.08-0.13) |
| East Asia | 2.49 (1.68-3.44) | | 2.01 (1.37-2.73) | | -0.19 | | | 747.15 (505.4-1030.84) | | 607.38 (414.73-824.47) | | -0.8 (-1.01--0.6) |
| Eastern Europe | 0.49 (0.32-0.68) | | 0.55 (0.36-0.76) | | 0.12 | | | 879.63 (584.76-1223.74) | | 1132.7 (750.71-1583.79) | | 0.04 (-0.2-0.28) |
| Eastern Sub-Saharan Africa | 0.48 (0.32-0.67) | | 1.3 (0.85-1.82) | | 1.71 | | | 1115.12 (739.15-1552.98) | | 1216.24 (796.74-1697.91) | | -0.17 (-0.31--0.04) |
| High-income Asia Pacific | 0.28 (0.19-0.38) | | 0.29 (0.2-0.4) | | 0.04 | | | 614.23 (421.78-835.34) | | 762.49 (516.06-1055.81) | | 0.3 (0.11-0.49) |
| High-income North America | 0.89 (0.62-1.21) | | 1.6 (1.13-2.16) | | 0.8 | | | 1192.4 (828.98-1628.2) | | 1900.89 (1345.95-2573.86) | | 0.61 (0.34-0.89) |
| North Africa and Middle East | 1.01 (0.68-1.42) | | 2.42 (1.57-3.45) | | 1.4 | | | 1287.49 (865.19-1813.29) | | 1518.75 (986.69-2163.34) | | 0.3 (0.16-0.45) |
| Oceania | 0.01 (0.01-0.02) | | 0.02 (0.02-0.04) | | 1 | | | 688.18 (460.18-966.69) | | 717.16 (444.54-1027.31) | | -0.07 (-0.12--0.01) |
| South Asia | 2.65 (1.76-3.65) | | 5.46 (3.65-7.53) | | 1.06 | | | 1038.02 (690.83-1431.34) | | 1105.56 (738.45-1523.45) | | -0.69 (-0.97--0.42) |
| Southeast Asia | | 0.69 (0.47-0.96) | | 1.26 (0.85-1.75) | | 0.83 | 575.77 (392.97-798.64) | | 688.09 (462.97-957.68) | | 0.13 (-0.03-0.28) | |
| Southern Latin America | | 0.13 (0.09-0.19) | | 0.21 (0.14-0.3) | | 0.62 | 1065.9 (729.5-1494.11) | | 1210.42 (804.24-1696.65) | | -0.22 (-0.45-0.02) | |
| Southern Sub-Saharan Africa | | 0.14 (0.09-0.19) | | 0.29 (0.2-0.39) | | 1.07 | 1019.71 (700.77-1395.42) | | 1327.02 (922.92-1817.69) | | 0.44 (0.19-0.69) | |
| Tropical Latin America | | 0.48 (0.32-0.66) | | 0.88 (0.58-1.2) | | 0.83 | 1196.96 (807.4-1648.72) | | 1446.23 (962.22-1978.83) | | -0.28 (-0.63-0.06) | |
| Western Europe | | 1.21 (0.84-1.64) | | 1.43 (0.97-1.99) | | 0.18 | 1264.22 (875.51-1720.96) | | 1533.8 (1037.16-2135.97) | | 0.2 (0.02-0.39) | |
| Western Sub-Saharan Africa | | 0.41 (0.28-0.58) | | 1.13 (0.76-1.58) | | 1.76 | 949.5 (633.71-1333.12) | | 942.73 (631.27-1320.02) | | -0.18 (-0.27--0.08) | |

**Abbreviations: WCBA, Women of Childbearing Age; EAPC, Estimated Annual Percentage Change; CI, Confidence Intervals; UI, Uncertainty Intervals; SDI, Socio-Demographic Index; DALYs, Disability-Adjusted Life Years.**

# **Table S2. Temporal Joinpoint analysis of depression prevalence rates in WCBA, 1990-2021**

| location | Start Obs | End Obs | measure | val | lower | upper | P.Value |
| --- | --- | --- | --- | --- | --- | --- | --- |
| Global | 1990 | 2021 | AAPC | 0.4789 | 0.3289 | 0.629 | <0.001 |
| Low SDI | 1990 | 2021 | AAPC | 0.1818 | 0.0765 | 0.2871 | 0.000706 |
| Low-middle SDI | 1990 | 2021 | AAPC | 0.3338 | 0.1439 | 0.524 | 0.000566 |
| Middle SDI | 1990 | 2021 | AAPC | 0.476 | 0.313 | 0.6393 | <0.001 |
| High-middle SDI | 1990 | 2021 | AAPC | 0.3155 | 0.1455 | 0.4859 | 0.000274 |
| High SDI | 1990 | 2021 | AAPC | 0.8997 | 0.6688 | 1.1312 | <0.001 |
| Andean Latin America | 1990 | 2021 | AAPC | 0.9299 | 0.5177 | 1.3439 | 0.000009 |
| Australasia | 1990 | 2021 | AAPC | 0.3586 | 0.3019 | 0.4154 | <0.001 |
| Caribbean | 1990 | 2021 | AAPC | 0.2275 | 0.1552 | 0.2999 | <0.001 |
| Central Asia | 1990 | 2021 | AAPC | 0.6292 | 0.5046 | 0.754 | <0.001 |
| Central Europe | 1990 | 2021 | AAPC | 0.4759 | 0.4088 | 0.5431 | <0.001 |
| Central Latin America | 1990 | 2021 | AAPC | 1.5241 | 1.312 | 1.7366 | <0.001 |
| Central Sub-Saharan Africa | 1990 | 2021 | AAPC | 0.2189 | 0.0494 | 0.3887 | 0.011348 |
| East Asia | 1990 | 2021 | AAPC | -0.3498 | -0.5315 | -0.1678 | 0.000167 |
| Eastern Europe | 1990 | 2021 | AAPC | 0.7387 | 0.708 | 0.7693 | <0.001 |
| Eastern Sub-Saharan Africa | 1990 | 2021 | AAPC | 0.2339 | 0.1803 | 0.2875 | <0.001 |
| High-income Asia Pacific | 1990 | 2021 | AAPC | 0.645 | 0.5685 | 0.7216 | <0.001 |
| High-income North America | 1990 | 2021 | AAPC | 1.3382 | 1.1976 | 1.4791 | <0.001 |
| North Africa and Middle East | 1990 | 2021 | AAPC | 0.6137 | 0.5096 | 0.7178 | <0.001 |
| Oceania | 1990 | 2021 | AAPC | 0.136 | 0.0716 | 0.2005 | 0.000035 |
| South Asia | 1990 | 2021 | AAPC | 0.2789 | 0.1524 | 0.4055 | 0.000015 |
| Southeast Asia | 1990 | 2021 | AAPC | 0.526 | 0.4944 | 0.5577 | <0.001 |
| Southern Latin America | 1990 | 2021 | AAPC | 0.5513 | 0.4165 | 0.6862 | <0.001 |
| Southern Sub-Saharan Africa | 1990 | 2021 | AAPC | 0.9084 | 0.7158 | 1.1013 | <0.001 |
| Tropical Latin America | 1990 | 2021 | AAPC | 0.6861 | 0.6281 | 0.7442 | <0.001 |
| Western Europe | 1990 | 2021 | AAPC | 0.7135 | 0.564 | 0.8633 | <0.001 |
| Western Sub-Saharan Africa | 1990 | 2021 | AAPC | 0.0252 | -0.1224 | 0.1731 | 0.738011 |
| Global | 1990 | 2005 | APC | 0.0479 | -0.0215 | 0.1173 | 0.165753 |
| Global | 2005 | 2010 | APC | -1.2934 | -1.7845 | -0.7999 | 0.000022 |
| Global | 2010 | 2019 | APC | 0.4191 | 0.2364 | 0.6022 | 0.000102 |
| Global | 2019 | 2021 | APC | 8.7824 | 6.7558 | 10.8476 | <0.001 |
| Low SDI | 1990 | 2005 | APC | 0.0696 | 0.0186 | 0.1205 | 0.009781 |
| Low SDI | 2005 | 2010 | APC | -1.571 | -1.9287 | -1.212 | 0 |
| Low SDI | 2010 | 2019 | APC | 0.0137 | -0.116 | 0.1436 | 0.828627 |
| Low SDI | 2019 | 2021 | APC | 6.386 | 5.0193 | 7.7705 | <0.001 |
| Low-middle SDI | 1990 | 2005 | APC | 0.2378 | 0.1469 | 0.3288 | 0.000021 |
| Low-middle SDI | 2005 | 2010 | APC | -2.5862 | -3.1977 | -1.9707 | <0.001 |
| Low-middle SDI | 2010 | 2019 | APC | 0.1325 | -0.0955 | 0.3609 | 0.240578 |
| Low-middle SDI | 2019 | 2021 | APC | 9.788 | 7.1948 | 12.4438 | <0.001 |
| Middle SDI | 1990 | 2006 | APC | -0.1437 | -0.1981 | -0.0892 | 0.000019 |
| Middle SDI | 2006 | 2009 | APC | -1.5137 | -2.8382 | -0.1711 | 0.029074 |
| Middle SDI | 2009 | 2019 | APC | 0.5572 | 0.4266 | 0.6881 | <0.001 |
| Middle SDI | 2019 | 2021 | APC | 8.3513 | 6.689 | 10.0395 | <0.001 |
| High-middle SDI | 1990 | 2010 | APC | -0.51 | -0.5669 | -0.4532 | <0.001 |
| High-middle SDI | 2010 | 2019 | APC | 0.6596 | 0.4189 | 0.9009 | 0.000008 |
| High-middle SDI | 2019 | 2021 | APC | 7.2911 | 4.6564 | 9.992 | 0.000005 |
| High SDI | 1990 | 1999 | APC | 0.9855 | 0.6505 | 1.3215 | 0.000003 |
| High SDI | 1999 | 2018 | APC | -0.1567 | -0.2749 | -0.0384 | 0.011586 |
| High SDI | 2018 | 2021 | APC | 7.5806 | 5.2826 | 9.9287 | <0.001 |
| Andean Latin America | 1990 | 2018 | APC | -0.2353 | -0.3616 | -0.1089 | 0.000718 |
| Andean Latin America | 2018 | 2021 | APC | 12.4832 | 7.7845 | 17.3868 | 0.000005 |
| Australasia | 1990 | 2000 | APC | 0.5563 | 0.5203 | 0.5923 | <0.001 |
| Australasia | 2000 | 2005 | APC | 1.5069 | 1.3816 | 1.6325 | <0.001 |
| Australasia | 2005 | 2019 | APC | -0.7589 | -0.7852 | -0.7325 | <0.001 |
| Australasia | 2019 | 2021 | APC | 4.4649 | 3.5926 | 5.3446 | <0.001 |
| Caribbean | 1990 | 2010 | APC | -0.8257 | -0.848 | -0.8034 | <0.001 |
| Caribbean | 2010 | 2019 | APC | 0.2494 | 0.154 | 0.3448 | 0.000015 |
| Caribbean | 2019 | 2021 | APC | 11.2857 | 10.0959 | 12.4884 | <0.001 |
| Central Asia | 1990 | 2011 | APC | -0.0362 | -0.075 | 0.0027 | 0.066742 |
| Central Asia | 2011 | 2019 | APC | 0.4962 | 0.2814 | 0.7114 | 0.000074 |
| Central Asia | 2019 | 2021 | APC | 8.4613 | 6.5409 | 10.4164 | <0.001 |
| Central Europe | 1990 | 1998 | APC | -0.2081 | -0.2971 | -0.1189 | 0.000085 |
| Central Europe | 1998 | 2010 | APC | -0.8469 | -0.9017 | -0.7921 | <0.001 |
| Central Europe | 2010 | 2019 | APC | 0.5937 | 0.4998 | 0.6876 | <0.001 |
| Central Europe | 2019 | 2021 | APC | 11.2183 | 10.2323 | 12.2132 | <0.001 |
| Central Latin America | 1990 | 2019 | APC | 0.7896 | 0.7397 | 0.8395 | <0.001 |
| Central Latin America | 2019 | 2021 | APC | 12.7964 | 9.1167 | 16.6001 | <0.001 |
| Central Sub-Saharan Africa | 1990 | 2018 | APC | -0.1375 | -0.1917 | -0.0833 | 0.000018 |
| Central Sub-Saharan Africa | 2018 | 2021 | APC | 3.6076 | 1.8004 | 5.4468 | 0.000311 |
| East Asia | 1990 | 1996 | APC | -0.2275 | -0.5352 | 0.0811 | 0.140065 |
| East Asia | 1996 | 1999 | APC | -2.4814 | -4.1859 | -0.7467 | 0.007414 |
| East Asia | 1999 | 2009 | APC | -0.9963 | -1.1588 | -0.8335 | <0.001 |
| East Asia | 2009 | 2021 | APC | 0.6734 | 0.5573 | 0.7897 | <0.001 |
| Eastern Europe | 1990 | 1995 | APC | 0.8728 | 0.8109 | 0.9347 | <0.001 |
| Eastern Europe | 1995 | 2001 | APC | -0.9814 | -1.0404 | -0.9225 | <0.001 |
| Eastern Europe | 2001 | 2010 | APC | -0.4384 | -0.4664 | -0.4104 | <0.001 |
| Eastern Europe | 2010 | 2015 | APC | 1.0622 | 0.9773 | 1.1471 | <0.001 |
| Eastern Europe | 2015 | 2019 | APC | 0.0221 | -0.1148 | 0.1591 | 0.735915 |
| Eastern Europe | 2019 | 2021 | APC | 12.1708 | 11.8702 | 12.4722 | <0.001 |
| Eastern Sub-Saharan Africa | 1990 | 2002 | APC | -0.0367 | -0.0739 | 0.0005 | 0.053114 |
| Eastern Sub-Saharan Africa | 2002 | 2013 | APC | -0.6353 | -0.6834 | -0.5872 | <0.001 |
| Eastern Sub-Saharan Africa | 2013 | 2019 | APC | 0.2754 | 0.1272 | 0.4237 | 0.000889 |
| Eastern Sub-Saharan Africa | 2019 | 2021 | APC | 6.739 | 6.0199 | 7.463 | <0.001 |
| High-income Asia Pacific | 1990 | 1995 | APC | -0.4952 | -0.6403 | -0.3499 | 0.000003 |
| High-income Asia Pacific | 1995 | 2000 | APC | 2.3723 | 2.1693 | 2.5758 | <0.001 |
| High-income Asia Pacific | 2000 | 2005 | APC | 0.2226 | 0.0405 | 0.405 | 0.019869 |
| High-income Asia Pacific | 2005 | 2015 | APC | -0.8398 | -0.8921 | -0.7875 | <0.001 |
| High-income Asia Pacific | 2015 | 2019 | APC | 0.0443 | -0.2627 | 0.3522 | 0.762913 |
| High-income Asia Pacific | 2019 | 2021 | APC | 9.327 | 8.4761 | 10.1845 | <0.001 |
| High-income North America | 1990 | 1997 | APC | 2.6995 | 2.4367 | 2.963 | <0.001 |
| High-income North America | 1997 | 2019 | APC | -0.1751 | -0.2218 | -0.1283 | <0.001 |
| High-income North America | 2019 | 2021 | APC | 14.122 | 11.8428 | 16.4476 | <0.001 |
| North Africa and Middle East | 1990 | 2001 | APC | -0.2508 | -0.3392 | -0.1623 | 0.000005 |
| North Africa and Middle East | 2001 | 2019 | APC | 0.3722 | 0.3253 | 0.4191 | <0.001 |
| North Africa and Middle East | 2019 | 2021 | APC | 7.8106 | 6.1406 | 9.5069 | <0.001 |
| Oceania | 1990 | 2006 | APC | 0.0087 | -0.0178 | 0.0352 | 0.501023 |
| Oceania | 2006 | 2011 | APC | -0.3326 | -0.5506 | -0.1141 | 0.004686 |
| Oceania | 2011 | 2019 | APC | 0.0709 | -0.0211 | 0.1631 | 0.123942 |
| Oceania | 2019 | 2021 | APC | 2.62 | 1.8028 | 3.4437 | 0.000001 |
| South Asia | 1990 | 1994 | APC | 1.7131 | 1.3116 | 2.1161 | <0.001 |
| South Asia | 1994 | 2006 | APC | -0.1732 | -0.2562 | -0.0901 | 0.000362 |
| South Asia | 2006 | 2010 | APC | -4.1623 | -4.7156 | -3.6058 | <0.001 |
| South Asia | 2010 | 2019 | APC | 0.0936 | -0.0337 | 0.2212 | 0.139966 |
| South Asia | 2019 | 2021 | APC | 10.5623 | 9.0683 | 12.0767 | <0.001 |
| Southeast Asia | 1990 | 2000 | APC | -0.1079 | -0.1262 | -0.0897 | <0.001 |
| Southeast Asia | 2000 | 2005 | APC | 0.2884 | 0.2156 | 0.3613 | <0.001 |
| Southeast Asia | 2005 | 2010 | APC | -0.2796 | -0.3524 | -0.2067 | 0.000001 |
| Southeast Asia | 2010 | 2013 | APC | 0.3165 | 0.0828 | 0.5507 | 0.011276 |
| Southeast Asia | 2013 | 2019 | APC | 0.4402 | 0.387 | 0.4933 | <0.001 |
| Southeast Asia | 2019 | 2021 | APC | 7.1014 | 6.8418 | 7.3616 | <0.001 |
| Southern Latin America | 1990 | 2006 | APC | -0.0926 | -0.135 | -0.0501 | 0.000182 |
| Southern Latin America | 2006 | 2009 | APC | -2.9077 | -3.9058 | -1.8992 | 0.000007 |
| Southern Latin America | 2009 | 2019 | APC | -0.0491 | -0.141 | 0.0429 | 0.279339 |
| Southern Latin America | 2019 | 2021 | APC | 14.9519 | 13.2848 | 16.6435 | <0.001 |
| Southern Sub-Saharan Africa | 1990 | 2006 | APC | -0.2879 | -0.3873 | -0.1885 | 0.000004 |
| Southern Sub-Saharan Africa | 2006 | 2019 | APC | 0.9098 | 0.7573 | 1.0626 | <0.001 |
| Southern Sub-Saharan Africa | 2019 | 2021 | APC | 11.0003 | 7.8821 | 14.2086 | <0.001 |
| Tropical Latin America | 1990 | 1995 | APC | 0.2108 | 0.0868 | 0.335 | 0.002495 |
| Tropical Latin America | 1995 | 2000 | APC | 2.7378 | 2.5692 | 2.9066 | <0.001 |
| Tropical Latin America | 2000 | 2006 | APC | 0.0914 | -0.0244 | 0.2074 | 0.113231 |
| Tropical Latin America | 2006 | 2010 | APC | -4.5907 | -4.8039 | -4.3771 | <0.001 |
| Tropical Latin America | 2010 | 2019 | APC | -0.4143 | -0.4627 | -0.366 | <0.001 |
| Tropical Latin America | 2019 | 2021 | APC | 15.3877 | 14.7449 | 16.0341 | <0.001 |
| Western Europe | 1990 | 2019 | APC | -0.0065 | -0.0343 | 0.0214 | 0.637579 |
| Western Europe | 2019 | 2021 | APC | 11.7558 | 9.1311 | 14.4437 | <0.001 |
| Western Sub-Saharan Africa | 1990 | 2004 | APC | 0.1849 | 0.0382 | 0.3318 | 0.015668 |
| Western Sub-Saharan Africa | 2004 | 2016 | APC | -0.679 | -0.8797 | -0.4779 | <0.001 |
| Western Sub-Saharan Africa | 2016 | 2021 | APC | 1.2824 | 0.5506 | 2.0196 | 0.001345 |

**Abbreviations: WCBA, Women of Childbearing Age; APC, annual percentage change; AAPC, average annual percentage change; SDI, Socio-Demographic Index.**

# **Table S3. Temporal Joinpoint Analysis of Depression DALYs Rates in WCBA, 1990-2021**

| location | Start Obs | End Obs | measure | val | lower | upper | P.Value |
| --- | --- | --- | --- | --- | --- | --- | --- |
| Global | 1990 | 2021 | AAPC | 0.524 | 0.3756 | 0.6725 | <0.001 |
| Low SDI | 1990 | 2021 | AAPC | 0.2348 | 0.1148 | 0.355 | 0.000124 |
| Low-middle SDI | 1990 | 2021 | AAPC | 0.3599 | 0.1423 | 0.5779 | 0.001178 |
| Middle SDI | 1990 | 2021 | AAPC | 0.455 | 0.2028 | 0.7078 | 0.000401 |
| High-middle SDI | 1990 | 2021 | AAPC | 0.2914 | 0.1233 | 0.4597 | 0.000675 |
| High SDI | 1990 | 2021 | AAPC | 1.1262 | 1.0556 | 1.1969 | <0.001 |
| Andean Latin America | 1990 | 2021 | AAPC | 1.0717 | 0.5649 | 1.5809 | 0.000032 |
| Australasia | 1990 | 2021 | AAPC | 0.4056 | 0.3551 | 0.456 | <0.001 |
| Caribbean | 1990 | 2021 | AAPC | 0.2228 | 0.1602 | 0.2854 | <0.001 |
| Central Asia | 1990 | 2021 | AAPC | 0.7203 | 0.5998 | 0.8409 | <0.001 |
| Central Europe | 1990 | 2021 | AAPC | 0.5459 | 0.4542 | 0.6377 | <0.001 |
| Central Latin America | 1990 | 2021 | AAPC | 1.6654 | 1.4376 | 1.8936 | <0.001 |
| Central Sub-Saharan Africa | 1990 | 2021 | AAPC | 0.2866 | 0.0885 | 0.4852 | 0.004557 |
| East Asia | 1990 | 2021 | AAPC | -0.6107 | -0.8531 | -0.3678 | 0.000001 |
| Eastern Europe | 1990 | 2021 | AAPC | 0.8336 | 0.794 | 0.8732 | <0.001 |
| Eastern Sub-Saharan Africa | 1990 | 2021 | AAPC | 0.3128 | 0.2513 | 0.3744 | <0.001 |
| High-income Asia Pacific | 1990 | 2021 | AAPC | 0.7443 | 0.6474 | 0.8414 | <0.001 |
| High-income North America | 1990 | 2021 | AAPC | 1.5849 | 1.3028 | 1.8678 | <0.001 |
| North Africa and Middle East | 1990 | 2021 | AAPC | 0.6572 | 0.5452 | 0.7693 | <0.001 |
| Oceania | 1990 | 2021 | AAPC | 0.1141 | 0.0384 | 0.1899 | 0.003131 |
| South Asia | 1990 | 2021 | AAPC | 0.1782 | -0.218 | 0.576 | 0.378511 |
| Southeast Asia | 1990 | 2021 | AAPC | 0.5757 | 0.5257 | 0.6257 | <0.001 |
| Southern Latin America | 1990 | 2021 | AAPC | 0.5157 | 0.1282 | 0.9048 | 0.009059 |
| Southern Sub-Saharan Africa | 1990 | 2021 | AAPC | 0.9874 | 0.775 | 1.2002 | <0.001 |
| Tropical Latin America | 1990 | 2021 | AAPC | 0.7061 | 0.6465 | 0.7659 | <0.001 |
| Western Europe | 1990 | 2021 | AAPC | 0.7949 | 0.6649 | 0.9251 | <0.001 |
| Western Sub-Saharan Africa | 1990 | 2021 | AAPC | 0.0631 | -0.1324 | 0.2591 | 0.527137 |
| Global | 1990 | 2005 | APC | 0.0449 | -0.0273 | 0.1172 | 0.209599 |
| Global | 2005 | 2010 | APC | -1.6155 | -2.1412 | -1.0871 | 0.000003 |
| Global | 2010 | 2019 | APC | 0.4509 | 0.2641 | 0.638 | 0.000056 |
| Global | 2019 | 2021 | APC | 10.308 | 8.3798 | 12.2706 | <0.001 |
| Low SDI | 1990 | 2005 | APC | 0.1133 | 0.0521 | 0.1745 | 0.00092 |
| Low SDI | 2005 | 2011 | APC | -1.6685 | -1.9823 | -1.3537 | <0.001 |
| Low SDI | 2011 | 2019 | APC | 0.1879 | -0.0057 | 0.3818 | 0.056506 |
| Low SDI | 2019 | 2021 | APC | 7.3403 | 5.7767 | 8.927 | <0.001 |
| Low-middle SDI | 1990 | 2005 | APC | 0.2734 | 0.1659 | 0.381 | 0.00003 |
| Low-middle SDI | 2005 | 2010 | APC | -3.1095 | -3.8708 | -2.3422 | <0.001 |
| Low-middle SDI | 2010 | 2019 | APC | 0.1448 | -0.1292 | 0.4195 | 0.284537 |
| Low-middle SDI | 2019 | 2021 | APC | 11.3674 | 8.525 | 14.2842 | <0.001 |
| Middle SDI | 1990 | 2012 | APC | -0.446 | -0.5265 | -0.3655 | <0.001 |
| Middle SDI | 2012 | 2019 | APC | 0.738 | 0.161 | 1.3183 | 0.014282 |
| Middle SDI | 2019 | 2021 | APC | 9.8334 | 6.094 | 13.7045 | 0.000009 |
| High-middle SDI | 1990 | 2010 | APC | -0.6931 | -0.7555 | -0.6306 | <0.001 |
| High-middle SDI | 2010 | 2019 | APC | 0.7472 | 0.495 | 1.0001 | 0.000002 |
| High-middle SDI | 2019 | 2021 | APC | 8.4529 | 5.8663 | 11.1027 | <0.001 |
| High SDI | 1990 | 2000 | APC | 1.2051 | 1.1266 | 1.2836 | <0.001 |
| High SDI | 2000 | 2019 | APC | -0.1616 | -0.194 | -0.1293 | <0.001 |
| High SDI | 2019 | 2021 | APC | 13.7765 | 12.6129 | 14.9521 | <0.001 |
| Andean Latin America | 1990 | 2018 | APC | -0.3102 | -0.4736 | -0.1466 | 0.000596 |
| Andean Latin America | 2018 | 2021 | APC | 14.9296 | 9.0872 | 21.0849 | 0.000009 |
| Australasia | 1990 | 2000 | APC | 0.6044 | 0.5613 | 0.6475 | <0.001 |
| Australasia | 2000 | 2005 | APC | 1.6796 | 1.5109 | 1.8486 | <0.001 |
| Australasia | 2005 | 2019 | APC | -0.8249 | -0.854 | -0.7958 | <0.001 |
| Australasia | 2019 | 2021 | APC | 5.0168 | 4.3362 | 5.7017 | <0.001 |
| Caribbean | 1990 | 2011 | APC | -0.9467 | -0.9676 | -0.9258 | <0.001 |
| Caribbean | 2011 | 2019 | APC | 0.4361 | 0.3227 | 0.5495 | <0.001 |
| Caribbean | 2019 | 2021 | APC | 12.4072 | 11.4144 | 13.4089 | <0.001 |
| Central Asia | 1990 | 2011 | APC | -0.0774 | -0.1174 | -0.0375 | 0.000527 |
| Central Asia | 2011 | 2019 | APC | 0.5346 | 0.3209 | 0.7488 | 0.000027 |
| Central Asia | 2019 | 2021 | APC | 10.3015 | 8.4344 | 12.2007 | <0.001 |
| Central Europe | 1990 | 1998 | APC | -0.3048 | -0.4287 | -0.1808 | 0.000047 |
| Central Europe | 1998 | 2011 | APC | -1.0574 | -1.1242 | -0.9905 | <0.001 |
| Central Europe | 2011 | 2019 | APC | 0.8038 | 0.6479 | 0.9599 | <0.001 |
| Central Europe | 2019 | 2021 | APC | 14.3018 | 12.9431 | 15.6769 | <0.001 |
| Central Latin America | 1990 | 2019 | APC | 0.862 | 0.8067 | 0.9173 | <0.001 |
| Central Latin America | 2019 | 2021 | APC | 14.0597 | 10.0808 | 18.1825 | <0.001 |
| Central Sub-Saharan Africa | 1990 | 2018 | APC | -0.1109 | -0.1777 | -0.0442 | 0.002068 |
| Central Sub-Saharan Africa | 2018 | 2021 | APC | 4.0746 | 1.9665 | 6.2263 | 0.000437 |
| East Asia | 1990 | 1995 | APC | -0.4133 | -1.1267 | 0.3053 | 0.244298 |
| East Asia | 1995 | 1999 | APC | -2.7791 | -4.281 | -1.2537 | 0.001139 |
| East Asia | 1999 | 2009 | APC | -1.4221 | -1.6993 | -1.1442 | <0.001 |
| East Asia | 2009 | 2021 | APC | 0.7258 | 0.541 | 0.9109 | <0.001 |
| Eastern Europe | 1990 | 1995 | APC | 0.9705 | 0.8934 | 1.0477 | <0.001 |
| Eastern Europe | 1995 | 2000 | APC | -1.2646 | -1.3696 | -1.1595 | <0.001 |
| Eastern Europe | 2000 | 2010 | APC | -0.5762 | -0.6063 | -0.546 | <0.001 |
| Eastern Europe | 2010 | 2015 | APC | 1.2005 | 1.0931 | 1.3079 | <0.001 |
| Eastern Europe | 2015 | 2019 | APC | -0.0815 | -0.2538 | 0.091 | 0.329725 |
| Eastern Europe | 2019 | 2021 | APC | 14.6867 | 14.2929 | 15.0817 | <0.001 |
| Eastern Sub-Saharan Africa | 1990 | 2002 | APC | 0.0185 | -0.025 | 0.062 | 0.386291 |
| Eastern Sub-Saharan Africa | 2002 | 2013 | APC | -0.7456 | -0.8018 | -0.6894 | <0.001 |
| Eastern Sub-Saharan Africa | 2013 | 2019 | APC | 0.3393 | 0.1698 | 0.5091 | 0.000436 |
| Eastern Sub-Saharan Africa | 2019 | 2021 | APC | 8.1452 | 7.3144 | 8.9825 | <0.001 |
| High-income Asia Pacific | 1990 | 1995 | APC | -0.601 | -0.7855 | -0.4161 | 0.000005 |
| High-income Asia Pacific | 1995 | 2000 | APC | 2.7408 | 2.4653 | 3.017 | <0.001 |
| High-income Asia Pacific | 2000 | 2006 | APC | 0.1583 | -0.032 | 0.349 | 0.096502 |
| High-income Asia Pacific | 2006 | 2015 | APC | -0.893 | -0.982 | -0.8039 | <0.001 |
| High-income Asia Pacific | 2015 | 2019 | APC | 0.044 | -0.3775 | 0.4673 | 0.827185 |
| High-income Asia Pacific | 2019 | 2021 | APC | 10.2035 | 9.2435 | 11.1719 | <0.001 |
| High-income North America | 1990 | 1999 | APC | 2.9539 | 2.5663 | 3.3429 | <0.001 |
| High-income North America | 1999 | 2019 | APC | -0.3224 | -0.4425 | -0.2022 | 0.000011 |
| High-income North America | 2019 | 2021 | APC | 15.6068 | 11.0362 | 20.3656 | <0.001 |
| North Africa and Middle East | 1990 | 2002 | APC | -0.2447 | -0.3343 | -0.155 | 0.000009 |
| North Africa and Middle East | 2002 | 2019 | APC | 0.3811 | 0.3208 | 0.4415 | <0.001 |
| North Africa and Middle East | 2019 | 2021 | APC | 8.7516 | 6.9639 | 10.5691 | <0.001 |
| Oceania | 1990 | 2006 | APC | -0.0326 | -0.0621 | -0.0031 | 0.031754 |
| Oceania | 2006 | 2010 | APC | -0.5531 | -0.9488 | -0.1559 | 0.008696 |
| Oceania | 2010 | 2019 | APC | -0.0041 | -0.0872 | 0.0791 | 0.920068 |
| Oceania | 2019 | 2021 | APC | 3.2073 | 2.3368 | 4.0852 | <0.001 |
| South Asia | 1990 | 2005 | APC | 0.2952 | 0.0994 | 0.4915 | 0.004984 |
| South Asia | 2005 | 2010 | APC | -4.4753 | -5.852 | -3.0785 | 0.000002 |
| South Asia | 2010 | 2019 | APC | 0.0474 | -0.4523 | 0.5496 | 0.845941 |
| South Asia | 2019 | 2021 | APC | 12.5044 | 7.3399 | 17.9174 | 0.000036 |
| Southeast Asia | 1990 | 2000 | APC | -0.3279 | -0.3568 | -0.299 | <0.001 |
| Southeast Asia | 2000 | 2005 | APC | 0.2164 | 0.0992 | 0.3337 | 0.001318 |
| Southeast Asia | 2005 | 2010 | APC | -0.4928 | -0.6098 | -0.3758 | <0.001 |
| Southeast Asia | 2010 | 2013 | APC | 0.3265 | -0.0413 | 0.6956 | 0.07798 |
| Southeast Asia | 2013 | 2019 | APC | 0.5274 | 0.4434 | 0.6115 | <0.001 |
| Southeast Asia | 2019 | 2021 | APC | 9.601 | 9.1862 | 10.0174 | <0.001 |
| Southern Latin America | 1990 | 2019 | APC | -0.4826 | -0.5713 | -0.3938 | <0.001 |
| Southern Latin America | 2019 | 2021 | APC | 16.1691 | 9.2578 | 23.5176 | 0.000029 |
| Southern Sub-Saharan Africa | 1990 | 2005 | APC | -0.5306 | -0.6593 | -0.4018 | <0.001 |
| Southern Sub-Saharan Africa | 2005 | 2019 | APC | 0.9696 | 0.8071 | 1.1323 | <0.001 |
| Southern Sub-Saharan Africa | 2019 | 2021 | APC | 13.2755 | 9.8203 | 16.8393 | <0.001 |
| Tropical Latin America | 1990 | 1995 | APC | 0.2298 | 0.1097 | 0.3501 | 0.000987 |
| Tropical Latin America | 1995 | 2000 | APC | 2.9378 | 2.7663 | 3.1096 | <0.001 |
| Tropical Latin America | 2000 | 2006 | APC | 0.0706 | -0.0442 | 0.1855 | 0.209541 |
| Tropical Latin America | 2006 | 2010 | APC | -5.091 | -5.3345 | -4.8469 | <0.001 |
| Tropical Latin America | 2010 | 2019 | APC | -0.4687 | -0.5249 | -0.4126 | <0.001 |
| Tropical Latin America | 2019 | 2021 | APC | 16.7041 | 16.0796 | 17.3319 | <0.001 |
| Western Europe | 1990 | 2019 | APC | -0.0071 | -0.0375 | 0.0233 | 0.63551 |
| Western Europe | 2019 | 2021 | APC | 13.1739 | 10.8797 | 15.5156 | <0.001 |
| Western Sub-Saharan Africa | 1990 | 2004 | APC | 0.206 | 0.0212 | 0.3911 | 0.030377 |
| Western Sub-Saharan Africa | 2004 | 2017 | APC | -0.7392 | -0.9672 | -0.5107 | 0.000001 |
| Western Sub-Saharan Africa | 2017 | 2021 | APC | 2.2044 | 0.9304 | 3.4945 | 0.001482 |

**Abbreviations: WCBA, Women of Childbearing Age; APC, annual percentage change; AAPC, average annual percentage change; SDI, Socio-Demographic Index; DALYs, Disability-Adjusted Life Years.**

# **Table S4. The prevalence of depression cases and rates among WCBA in 1990 and 2021 across 204 countries, and the trends from 1990 to 2021.**

| location | Prevalent cases | | | Prevalent rates | | |
| --- | --- | --- | --- | --- | --- | --- |
|  | 1990_thousands(95% UI) | 2021_thousands(95% UI) | percentage change(100%) | 1990_per 100 000(95% UI) | 2021_per 100 000(95% UI) | EAPC(95% CI) |
| American Samoa | 0.43 (0.36-0.52) | 0.47 (0.37-0.59) | 0.09 | 3558.26 (2962.17-4348.83) | 3995.62 (3176.44-5035.14) | 0.17 (0.12-0.23) |
| Antigua and Barbuda | 0.74 (0.6-0.92) | 1.39 (1.06-1.85) | 0.88 | 4528.77 (3686.6-5691.16) | 5755.74 (4379.96-7657.29) | 0.34 (0.15-0.53) |
| Arab Republic of Egypt | 816.66 (661.97-1015.1) | 1949.84 (1479.47-2546.71) | 1.39 | 6227.68 (5048.03-7740.98) | 7514.5 (5701.74-9814.76) | 0.27 (0.09-0.44) |
| Argentine Republic | 375.2 (308.01-463.78) | 656.16 (522.87-812.21) | 0.75 | 4674.37 (3837.26-5777.94) | 5520.32 (4398.95-6833.14) | -0.14 (-0.37-0.1) |
| Australia | 347.65 (302.86-400.16) | 519.83 (405.83-659.93) | 0.5 | 7788.83 (6785.36-8965.15) | 8628.89 (6736.59-10954.38) | 0.18 (0.02-0.34) |
| Barbados | 3.14 (2.56-3.96) | 4.3 (3.3-5.71) | 0.37 | 4577.44 (3730.04-5781.69) | 6045.79 (4631.16-8026.63) | 0.37 (0.19-0.55) |
| Belize | 1.86 (1.53-2.39) | 6.85 (5.25-8.87) | 2.68 | 4420.81 (3622.71-5680.53) | 5672.62 (4342.7-7341.8) | 0.45 (0.29-0.62) |
| Bermuda | 0.94 (0.77-1.16) | 0.81 (0.61-1.06) | -0.14 | 5520.31 (4501.37-6781.87) | 6042.1 (4531.65-7842.2) | -0.26 (-0.46--0.07) |
| Bolivarian Republic of Venezuela | 214.57 (175.6-272.49) | 356.24 (268.98-466.23) | 0.66 | 4432.8 (3627.72-5629.35) | 5190.12 (3918.85-6792.59) | 0.23 (0.12-0.33) |
| Bosnia and Herzegovina | 60.18 (49.84-73.1) | 41.04 (31.62-52.4) | -0.32 | 5168.76 (4281.14-6278.73) | 5609.55 (4322.21-7162.01) | -0.5 (-0.72--0.28) |
| Brunei Darussalam | 1.9 (1.56-2.39) | 3.95 (3.12-5.16) | 1.08 | 2806.96 (2303.01-3533.57) | 3160.63 (2499.2-4129.54) | 0.11 (-0.05-0.28) |
| Burkina Faso | 120.09 (98.15-148.37) | 318.51 (249.38-409.02) | 1.65 | 5737.02 (4688.87-7088.28) | 5798.57 (4540.02-7446.38) | 0.26 (0.16-0.36) |
| Canada | 459.02 (393.31-534.49) | 653.78 (506.81-843.55) | 0.42 | 6239.93 (5346.67-7265.93) | 7882.65 (6110.58-10170.66) | 0.03 (-0.2-0.27) |
| Central African Republic | 57.57 (46.77-73.15) | 130.05 (98.09-170.8) | 1.26 | 8981.41 (7295.8-11412.03) | 9400.22 (7090.02-12346) | 0.01 (-0.08-0.11) |
| Commonwealth of Dominica | 0.75 (0.61-0.96) | 0.94 (0.7-1.25) | 0.25 | 4426.13 (3605.97-5656.79) | 5728.5 (4281.13-7612.92) | 0.34 (0.16-0.53) |
| Commonwealth of the Bahamas | 3.28 (2.68-4.15) | 6.32 (4.74-8.29) | 0.93 | 4523.56 (3690.61-5726.77) | 5858.55 (4398.6-7689.61) | 0.37 (0.16-0.57) |
| Cook Islands | 0.2 (0.16-0.24) | 0.21 (0.16-0.27) | 0.05 | 4263.5 (3477.58-5320.55) | 4857.16 (3792.99-6332.92) | 0.25 (0.21-0.3) |
| Czech Republic | 132.45 (110.63-157.45) | 128.66 (100.46-163.37) | -0.03 | 5144.56 (4297.12-6115.64) | 5608.72 (4379.45-7122.07) | -0.3 (-0.5--0.1) |
| Democratic People's Republic of Korea | 223.32 (186.35-270.63) | 264.16 (211.45-331.52) | 0.18 | 3937.95 (3286.17-4772.21) | 4008.53 (3208.69-5030.73) | -0.04 (-0.06--0.01) |
| Democratic Republic of Sao Tome and Principe | 1.36 (1.13-1.7) | 3.29 (2.62-4.14) | 1.42 | 5311.17 (4386.25-6609.79) | 5864.14 (4665.32-7376.23) | 0.24 (0.15-0.32) |
| Democratic Republic of the Congo | 720.61 (591.11-896.16) | 1849.04 (1414.65-2402.09) | 1.57 | 8443.42 (6925.99-10500.25) | 8677.9 (6639.23-11273.47) | -0.04 (-0.13-0.06) |
| Democratic Republic of Timor-Leste | 7.97 (6.65-9.85) | 14.48 (11.49-19) | 0.82 | 4276.91 (3566.04-5283.94) | 4182.79 (3318.67-5489.29) | -0.57 (-0.72--0.41) |
| Democratic Socialist Republic of Sri Lanka | 230.43 (194.37-272.07) | 277.4 (224.93-345.04) | 0.2 | 5019.44 (4233.8-5926.38) | 4912.24 (3983.02-6110) | -0.64 (-0.87--0.41) |
| Dominican Republic | 104.03 (84.32-132.16) | 192.31 (142.84-254.25) | 0.85 | 5510.08 (4466.12-7000.13) | 6638.74 (4931-8776.93) | 0.26 (0.09-0.43) |
| Eastern Republic of Uruguay | 37.46 (31.01-46.55) | 58.63 (44.42-75.64) | 0.57 | 4988.11 (4128.9-6198.54) | 7043.52 (5336.18-9087.43) | 0.79 (0.6-0.98) |
| Federal Democratic Republic of Ethiopia | 728.81 (627.72-861.36) | 1787.21 (1510.05-2137.46) | 1.45 | 6459.03 (5563.17-7633.77) | 6447.58 (5447.67-7711.15) | -0.33 (-0.51--0.15) |
| Federal Democratic Republic of Nepal | 270.99 (223.66-330.49) | 740.82 (563.73-959.5) | 1.73 | 5932.98 (4896.65-7235.64) | 8166.63 (6214.44-10577.27) | 0.6 (0.4-0.81) |
| Federal Republic of Germany | 1019.81 (888.93-1179.18) | 1192.13 (920.37-1571.51) | 0.17 | 5247.22 (4573.82-6067.22) | 6999.86 (5404.19-9227.5) | 0.89 (0.76-1.02) |
| Federal Republic of Nigeria | 1111.84 (952.86-1312.91) | 2881.91 (2479.49-3361.8) | 1.59 | 5490.03 (4705.02-6482.91) | 5018.93 (4318.1-5854.67) | -0.44 (-0.59--0.3) |
| Federal Republic of Somalia | 114.7 (94.64-140.02) | 381.75 (299.03-489.21) | 2.33 | 6772.36 (5587.86-8267.05) | 7893.2 (6182.81-10115.18) | 0.04 (-0.1-0.18) |
| Federated States of Micronesia | 0.91 (0.75-1.12) | 1.09 (0.86-1.38) | 0.2 | 3929.58 (3246.63-4810.23) | 4161.62 (3286.01-5287.79) | -0.01 (-0.08-0.05) |
| Federative Republic of Brazil | 2539.49 (2193.88-2972.47) | 4589.45 (3913.02-5372.83) | 0.81 | 6519.72 (5632.42-7631.32) | 7816.37 (6664.34-9150.56) | -0.23 (-0.56-0.09) |
| French Republic | 1151.97 (1044.44-1276.81) | 1192.02 (924.1-1540.31) | 0.03 | 7960.01 (7217-8822.7) | 8396.09 (6508.99-10849.3) | -0.24 (-0.42--0.05) |
| Gabonese Republic | 18.11 (14.69-22.58) | 45.04 (34.21-58.95) | 1.49 | 8179.21 (6634.18-10200.82) | 9199.61 (6987.85-12041.6) | 0 (-0.12-0.12) |
| Georgia | 59.94 (49.83-73.48) | 43.98 (33.69-56.08) | -0.27 | 4366.93 (3630.01-5352.93) | 5589.06 (4281.39-7125.62) | 0.31 (0.14-0.47) |
| Grand Duchy of Luxembourg | 6.78 (5.66-8.16) | 10.2 (7.95-12.69) | 0.5 | 6981.89 (5821.97-8399.14) | 6550.34 (5107.72-8151.27) | -0.68 (-0.88--0.48) |
| Greenland | 2.11 (1.74-2.6) | 1.99 (1.56-2.54) | -0.06 | 14321.84 (11791.88-17634.68) | 15638.17 (12258.72-19916.72) | -0.37 (-0.58--0.15) |
| Grenada | 0.88 (0.71-1.12) | 1.47 (1.13-1.88) | 0.67 | 4504.07 (3656.19-5772.32) | 5744.88 (4396.7-7337.03) | 0.37 (0.21-0.52) |
| Guam | 1.44 (1.18-1.75) | 1.74 (1.41-2.16) | 0.21 | 4117.42 (3387.4-5008.6) | 4819.67 (3920.9-5998.78) | 0.2 (0.09-0.31) |
| Hashemite Kingdom of Jordan | 61.06 (49.23-77.07) | 244.69 (186.22-329.35) | 3.01 | 7321.36 (5903.64-9241.9) | 7895.86 (6008.98-10627.61) | -0.1 (-0.22-0.02) |
| Hellenic Republic | 219.76 (177.32-278.75) | 235.56 (173.73-316.64) | 0.07 | 8721.5 (7037.27-11062.61) | 10867.51 (8014.92-14608.04) | 0.02 (-0.32-0.36) |
| Hungary | 136.17 (115.93-163.34) | 111.48 (88.39-139.35) | -0.18 | 5372.18 (4573.74-6444.06) | 5212.11 (4132.55-6514.93) | -0.65 (-0.84--0.45) |
| Independent State of Papua New Guinea | 42.03 (34.38-52.34) | 116.04 (92.47-145.47) | 1.76 | 4361.16 (3567.49-5431.46) | 4435.25 (3534.54-5560.15) | -0.08 (-0.12--0.04) |
| Independent State of Samoa | 1.4 (1.17-1.69) | 1.97 (1.52-2.47) | 0.41 | 3794.44 (3166.08-4588.47) | 4049.84 (3129.21-5085.92) | -0.03 (-0.09-0.04) |
| Ireland | 60.03 (51.36-70.35) | 106.41 (82.01-135.59) | 0.77 | 6798.45 (5816.53-7967.21) | 9120.29 (7029.27-11621.66) | 0.4 (0.2-0.6) |
| Islamic Republic of Afghanistan | 168.98 (137.73-207.63) | 650.1 (493.12-848.42) | 2.85 | 7679.79 (6259.54-9436.55) | 9035.01 (6853.43-11791.33) | 0.17 (0.04-0.3) |
| Islamic Republic of Iran | 1060.51 (898.52-1302.31) | 2353.35 (1978.06-2856.21) | 1.22 | 8385.13 (7104.38-10297) | 10123.93 (8509.47-12287.18) | 0.77 (0.56-0.98) |
| Islamic Republic of Mauritania | 22.92 (19.08-28.41) | 54.65 (42.59-70.06) | 1.38 | 4875.5 (4059.41-6043.34) | 5091.43 (3967.98-6527.09) | -0.05 (-0.15-0.05) |
| Islamic Republic of Pakistan | 1333.36 (1145.69-1588.64) | 3807.81 (3188.22-4604.84) | 1.86 | 5636.76 (4843.41-6715.96) | 6286.18 (5263.33-7601.98) | 0.15 (-0.01-0.3) |
| Jamaica | 26.52 (21.55-33.63) | 45.31 (33.9-60.1) | 0.71 | 4450.17 (3617.25-5644.12) | 5868.79 (4390.02-7784.21) | 0.34 (0.13-0.55) |
| Japan | 1104.9 (977.99-1246.65) | 1088.95 (956.61-1257.96) | -0.01 | 3441.73 (3046.43-3883.28) | 4379.19 (3847.01-5058.9) | 0.32 (0.07-0.58) |
| Kingdom of Bahrain | 9.52 (7.72-11.97) | 29.29 (21.99-38.1) | 2.08 | 8259.64 (6694.07-10385.6) | 8984.47 (6745.07-11685.41) | -0.26 (-0.42--0.1) |
| Kingdom of Belgium | 138.86 (125.25-156.92) | 183.59 (143.03-230.93) | 0.32 | 5703.73 (5144.66-6445.62) | 7409.84 (5772.94-9320.97) | 0.75 (0.57-0.93) |
| Kingdom of Bhutan | 7.72 (6.33-9.51) | 11.59 (9-15.03) | 0.5 | 5393.84 (4420.65-6643.44) | 5596.09 (4347.19-7261.11) | 0 (-0.13-0.13) |
| Kingdom of Cambodia | 113.72 (95.11-142.64) | 212.65 (166.01-264.57) | 0.87 | 4541.02 (3797.76-5695.72) | 4706.26 (3674.08-5855.15) | -0.46 (-0.61--0.32) |
| Kingdom of Denmark | 105.81 (88.58-124.63) | 92.01 (71.33-118.54) | -0.13 | 8104.13 (6784.07-9544.92) | 7284.77 (5647.48-9384.83) | -0.84 (-1.08--0.61) |
| Kingdom of Eswatini | 11.05 (9.05-13.51) | 26.89 (20.41-35.18) | 1.43 | 5672.07 (4644.93-6930.78) | 8533.02 (6475.21-11160.57) | 0.58 (0.35-0.82) |
| Kingdom of Lesotho | 32.49 (27.16-40.38) | 54.74 (42.56-70.88) | 0.68 | 8587.97 (7178.19-10673.26) | 10798.47 (8396.93-13983.26) | 0.29 (0.06-0.52) |
| Kingdom of Morocco | 502.45 (409.41-628.36) | 916.6 (712.21-1202.12) | 0.82 | 7978.37 (6501.02-9977.74) | 9463.34 (7353.16-12411.16) | 0.16 (-0.02-0.35) |
| Kingdom of Norway | 67.05 (57.72-79.12) | 92.64 (77.12-110.57) | 0.38 | 6352.24 (5468.21-7495.95) | 7649 (6367.38-9129.95) | 0.22 (0.07-0.36) |
| Kingdom of Saudi Arabia | 212.13 (171.39-268.64) | 792.61 (613.4-1038.66) | 2.74 | 6504.86 (5255.8-8237.71) | 7811.87 (6045.6-10237) | 0.49 (0.35-0.63) |
| Kingdom of Spain | 687.67 (622.53-772.35) | 1003.41 (794.84-1257.08) | 0.46 | 7140.36 (6464.03-8019.61) | 10132.18 (8026.06-12693.62) | 1.24 (0.91-1.58) |
| Kingdom of Sweden | 173.24 (151.03-197.74) | 215.58 (174.67-262.97) | 0.24 | 8439.42 (7357.61-9632.62) | 9812.54 (7950.46-11969.58) | 0.07 (-0.07-0.22) |
| Kingdom of Thailand | 645.29 (533.34-790.6) | 748.43 (585.68-941.31) | 0.16 | 4035.55 (3335.4-4944.28) | 4591.17 (3592.82-5774.41) | 0.16 (0.09-0.22) |
| Kingdom of the Netherlands | 260.28 (233.7-291.68) | 279.3 (216.65-362.85) | 0.07 | 6569.67 (5898.73-7362.42) | 7611.16 (5903.83-9888.03) | -0.08 (-0.3-0.14) |
| Kingdom of Tonga | 0.83 (0.69-1.02) | 1.02 (0.82-1.3) | 0.23 | 3730.81 (3098.47-4546.91) | 4033.61 (3258.02-5159.19) | 0.06 (0-0.12) |
| Kyrgyz Republic | 49.31 (40.5-59.49) | 98.45 (77.32-123.58) | 1 | 4704.04 (3863.9-5675.74) | 5710.03 (4484.61-7167.83) | 0.17 (0-0.34) |
| Lao People's Democratic Republic | 37.25 (31.24-45.81) | 77.28 (61.01-97.52) | 1.07 | 3849.64 (3228.54-4733.64) | 3894.58 (3074.41-4914.29) | -0.24 (-0.43--0.05) |
| Lebanese Republic | 51.11 (42.65-62.6) | 154.54 (116.09-201.73) | 2.02 | 6820.64 (5691.25-8353.17) | 10416.51 (7825.02-13597.53) | 0.49 (0.27-0.71) |
| Malaysia | 186.34 (154.69-229.25) | 413.1 (324.81-522.76) | 1.22 | 4158.94 (3452.53-5116.58) | 4902.33 (3854.5-6203.63) | 0.77 (0.6-0.94) |
| Mongolia | 28.74 (23.72-35.1) | 50.68 (40.71-65.36) | 0.76 | 5616.09 (4634.24-6859.57) | 6006.35 (4823.88-7745.78) | 0.05 (0.01-0.09) |
| Montenegro | 6.96 (5.79-8.3) | 8.29 (6.46-10.69) | 0.19 | 4458.35 (3710.41-5314.97) | 5754.23 (4486.68-7423.7) | 0.2 (0.02-0.38) |
| New Zealand | 60.17 (50.97-72.88) | 84.43 (67.93-105.23) | 0.4 | 6656.55 (5638.48-8062.78) | 7066.92 (5685.38-8807.39) | 0.05 (0-0.11) |
| North Macedonia | 21.45 (17.99-26.15) | 28.81 (22.73-36.49) | 0.34 | 4215.69 (3535.57-5139.03) | 5428.83 (4283.64-6875.35) | 0.11 (-0.13-0.36) |
| Northern Mariana Islands | 0.48 (0.39-0.59) | 0.5 (0.4-0.63) | 0.04 | 3507.63 (2821.59-4303.93) | 4378.41 (3502.91-5504.48) | 0.42 (0.31-0.53) |
| Palestine | 38.2 (30.89-48.46) | 131.09 (99.25-173.06) | 2.43 | 8641.92 (6986.97-10962.69) | 10067.64 (7622.61-13290.53) | 0.1 (-0.07-0.27) |
| People's Democratic Republic of Algeria | 386.86 (312.35-490.77) | 860.1 (668.48-1154.95) | 1.22 | 6657.04 (5374.84-8445.08) | 7663.55 (5956.17-10290.61) | 0.26 (0.11-0.4) |
| People's Republic of Bangladesh | 1779.73 (1466.28-2182.07) | 3801.69 (2928.45-4916.54) | 1.14 | 7228.93 (5955.74-8863.15) | 8265.4 (6366.85-10689.22) | 0.13 (-0.01-0.27) |
| People's Republic of China | 14618.43 (12843.15-16790.74) | 12975.94 (11306.1-15071.16) | -0.11 | 4536.92 (3985.95-5211.11) | 4071.55 (3547.6-4728.99) | -0.54 (-0.7--0.37) |
| Plurinational State of Bolivia | 82.36 (68.18-103.46) | 219.7 (167.42-291.08) | 1.67 | 5383.58 (4456.64-6762.75) | 7046.26 (5369.46-9335.69) | 0.13 (-0.2-0.46) |
| Portuguese Republic | 233.56 (193.65-283.13) | 241.64 (181.76-322.54) | 0.03 | 9232.08 (7654.43-11191.31) | 10380.37 (7807.85-13855.54) | -0.36 (-0.64--0.07) |
| Principality of Andorra | 0.99 (0.83-1.22) | 1.57 (1.2-2.08) | 0.59 | 6608.08 (5529.46-8170.65) | 7837.3 (5988.22-10390.09) | 0.14 (-0.06-0.33) |
| Principality of Monaco | 0.51 (0.4-0.64) | 0.63 (0.47-0.84) | 0.24 | 7266.8 (5732.26-9178.03) | 8836.15 (6590.49-11767.32) | 0.16 (0.02-0.31) |
| Puerto Rico | 37.64 (31.27-45.53) | 35.25 (27.49-45.35) | -0.06 | 3929.14 (3263.62-4752.57) | 4700.99 (3665.57-6047.94) | 0.2 (0.02-0.38) |
| Republic of Albania | 31.76 (26.49-39.42) | 31.19 (24.44-40.15) | -0.02 | 3803.37 (3171.83-4720.54) | 5081.76 (3982.93-6541.33) | 0.39 (0.22-0.57) |
| Republic of Angola | 207.23 (167.81-260.58) | 750.6 (579.68-1000.55) | 2.62 | 9001.82 (7289.59-11319.52) | 9779.78 (7552.76-13036.51) | -0.01 (-0.11-0.1) |
| Republic of Armenia | 34.41 (28.61-42.41) | 38.84 (30.09-49.49) | 0.13 | 3972.49 (3303.69-4896.19) | 5271.38 (4083.2-6716.39) | 0.42 (0.22-0.62) |
| Republic of Austria | 125.5 (106.19-150.52) | 126.19 (96.78-160.14) | 0.01 | 6341.87 (5366.09-7606.08) | 6391.11 (4901.48-8110.39) | -0.49 (-0.71--0.28) |
| Republic of Azerbaijan | 72.64 (60.22-89.77) | 138.36 (109.51-174.85) | 0.9 | 3874.02 (3211.59-4787.17) | 5047.65 (3995.09-6378.74) | 0.4 (0.22-0.59) |
| Republic of Belarus | 142.91 (118.9-172.13) | 174.83 (135.25-224.85) | 0.22 | 5640.33 (4692.88-6793.68) | 8244.04 (6377.48-10602.69) | 0.38 (0.16-0.59) |
| Republic of Benin | 64.51 (52.89-80.53) | 204.2 (160.77-267.72) | 2.17 | 5889.24 (4828.27-7350.95) | 6294.41 (4955.76-8252.6) | 0.05 (-0.02-0.11) |
| Republic of Botswana | 18.38 (15.05-22.97) | 52.18 (40.69-67.62) | 1.84 | 5709.77 (4674.8-7134.21) | 7669.38 (5980.97-9938.14) | 0.37 (0.23-0.51) |
| Republic of Bulgaria | 95.9 (79.9-117.05) | 82.53 (64.71-105.1) | -0.14 | 4631.01 (3858.14-5652.12) | 5793.67 (4542.75-7378.42) | -0.11 (-0.35-0.13) |
| Republic of Burundi | 92.41 (76.1-114.61) | 222.61 (168.17-288.38) | 1.41 | 7338 (6042.76-9100.57) | 7117.88 (5377.17-9220.87) | -0.58 (-0.74--0.41) |
| Republic of Cabo Verde | 4.44 (3.61-5.62) | 11.25 (8.6-14.53) | 1.53 | 5603.49 (4548.14-7087.68) | 7484.86 (5721.11-9665.07) | 0.5 (0.35-0.65) |
| Republic of Cameroon | 144.37 (117.91-178.46) | 512.7 (399.8-666.01) | 2.55 | 6067.69 (4955.29-7500.09) | 6545.6 (5104.29-8502.95) | 0.08 (0.02-0.14) |
| Republic of Chad | 91.62 (74.81-114.36) | 272.09 (204.85-361.56) | 1.97 | 6890.5 (5626.45-8600.54) | 7034.38 (5296.13-9347.59) | 0.01 (-0.06-0.08) |
| Republic of Chile | 298.42 (265.36-341.16) | 413.41 (312.1-554.24) | 0.39 | 8256.18 (7341.48-9438.53) | 8776.98 (6626.05-11766.81) | -0.38 (-0.61--0.15) |
| Republic of Colombia | 285.92 (235.26-352.63) | 424.89 (332.35-530.18) | 0.49 | 3292.18 (2708.92-4060.3) | 3243.33 (2536.97-4047.04) | -0.69 (-1.01--0.37) |
| Republic of Costa Rica | 34.44 (28.06-42.6) | 77.02 (57.13-101.73) | 1.24 | 4429.02 (3607.89-5478.78) | 5949.29 (4412.92-7858.76) | 0.52 (0.36-0.68) |
| Republic of Côte d'Ivoire | 144.68 (118.58-179.42) | 371.74 (289.56-466.6) | 1.57 | 5287.18 (4333.21-6556.75) | 5582.67 (4348.45-7007.25) | 0.09 (0.03-0.16) |
| Republic of Croatia | 62.54 (52.12-74.68) | 47.75 (37.44-59.85) | -0.24 | 5208.45 (4340.61-6219.73) | 5323.34 (4174.2-6672.42) | -0.48 (-0.67--0.29) |
| Republic of Cuba | 246.68 (208.1-294.21) | 167.3 (132.4-220.12) | -0.32 | 8050.57 (6791.4-9601.85) | 6721.88 (5319.79-8844.28) | -1.36 (-1.64--1.09) |
| Republic of Cyprus | 11.2 (9.27-13.97) | 23.45 (18.11-30.82) | 1.09 | 5667.86 (4694.61-7072.24) | 6566.37 (5070.67-8630.82) | 0.17 (0-0.33) |
| Republic of Djibouti | 5.9 (4.77-7.41) | 22.63 (17.68-28.59) | 2.84 | 6001.66 (4850.45-7534.31) | 7042.2 (5502.74-8899.26) | 0.31 (0.21-0.41) |
| Republic of Ecuador | 124.09 (102.03-152.68) | 300.05 (234.05-386.1) | 1.42 | 4920.11 (4045.44-6053.9) | 6363.03 (4963.32-8187.71) | 0.29 (0.03-0.54) |
| Republic of El Salvador | 79.82 (65.07-98.01) | 114.77 (87.2-152.12) | 0.44 | 6089.71 (4964.06-7477) | 6461.05 (4909.22-8563.78) | -0.3 (-0.51--0.09) |
| Republic of Equatorial Guinea | 8.97 (7.33-11.32) | 35.02 (27.08-46.24) | 2.9 | 9083.41 (7421.09-11459.63) | 9608.97 (7428.94-12685.68) | 0.05 (-0.06-0.17) |
| Republic of Estonia | 26.7 (22.28-32.05) | 19.34 (15.01-24.72) | -0.28 | 7008.07 (5845.76-8410.61) | 6948.87 (5391.56-8880.01) | -0.9 (-1.13--0.68) |
| Republic of Fiji | 7.81 (6.49-9.42) | 10.58 (8.41-13.36) | 0.35 | 3995.05 (3321.88-4819.44) | 4633.33 (3684.12-5848.39) | 0.12 (0.02-0.21) |
| Republic of Finland | 111.55 (94.21-132.05) | 104.15 (82.56-131.22) | -0.07 | 8829.73 (7457.2-10452.77) | 9163.62 (7264.56-11545.38) | -0.46 (-0.65--0.26) |
| Republic of Ghana | 208.17 (171.53-259.24) | 591.4 (462.31-763.68) | 1.84 | 5902.31 (4863.52-7350.39) | 6477.29 (5063.46-8364.26) | 0.05 (-0.04-0.14) |
| Republic of Guatemala | 96 (77.72-123.82) | 289.72 (215.88-399.57) | 2.02 | 5225.53 (4230.39-6739.76) | 6591.44 (4911.49-9090.74) | 0.18 (-0.02-0.39) |
| Republic of Guinea | 83.56 (68.86-103.39) | 219.12 (173.36-283.22) | 1.62 | 6126.1 (5048.16-7579.71) | 6602.89 (5223.84-8534.27) | 0.02 (-0.08-0.12) |
| Republic of Guinea-Bissau | 14.22 (11.65-17.93) | 35.51 (27.52-45.46) | 1.5 | 6097.65 (4995.66-7684.95) | 6760.37 (5238.83-8654.1) | 0.17 (0.07-0.26) |
| Republic of Guyana | 13.95 (11.32-16.74) | 20.28 (15.49-27) | 0.45 | 6828.58 (5541.03-8195.63) | 9981.22 (7623.71-13289.62) | 0.6 (0.42-0.79) |
| Republic of Haiti | 86.69 (70.16-110.9) | 230.96 (175.37-302.92) | 1.66 | 5634.61 (4559.86-7207.74) | 6524.39 (4953.99-8557.09) | 0.06 (-0.09-0.22) |
| Republic of Honduras | 44.16 (36.08-56.34) | 161.82 (120.23-210.92) | 2.66 | 4185.92 (3419.91-5340.38) | 5700.83 (4235.88-7430.88) | 0.43 (0.21-0.66) |
| Republic of Iceland | 3.83 (3.22-4.6) | 4.61 (3.56-5.84) | 0.2 | 5904.83 (4960.98-7094.53) | 5789.07 (4471.05-7329.75) | -0.36 (-0.5--0.21) |
| Republic of India | 11776.15 (10249.27-13687.54) | 22836.7 (19666.09-26556.82) | 0.94 | 5832.45 (5076.22-6779.12) | 6036.84 (5198.69-7020.24) | -0.79 (-1.06--0.52) |
| Republic of Indonesia | 1624.37 (1403.77-1892.46) | 3189.28 (2739.6-3726.32) | 0.96 | 3389.44 (2929.13-3948.83) | 4235.79 (3638.56-4949.06) | 0.39 (0.26-0.52) |
| Republic of Iraq | 262.74 (216.25-319.87) | 755.61 (587.52-977.09) | 1.88 | 6475.03 (5329.33-7883.1) | 7167.29 (5572.88-9268.11) | 0.26 (-0.11-0.64) |
| Republic of Italy | 949.55 (812.5-1119.05) | 989.54 (839.25-1175.47) | 0.04 | 6643.54 (5684.66-7829.44) | 8155.51 (6916.93-9687.98) | -0.18 (-0.45-0.09) |
| Republic of Kazakhstan | 192.89 (159.92-231.54) | 270.34 (209.56-331.83) | 0.4 | 4687.59 (3886.45-5626.8) | 5708.4 (4425.01-7006.92) | 0.46 (0.33-0.59) |
| Republic of Kenya | 328.83 (284.6-385.31) | 893.84 (767.34-1044.83) | 1.72 | 6348.81 (5494.84-7439.13) | 6734.46 (5781.37-7872.02) | -0.08 (-0.19-0.03) |
| Republic of Kiribati | 0.77 (0.64-0.93) | 1.33 (1.05-1.66) | 0.73 | 4102.01 (3401.76-4970.05) | 4169.24 (3292.69-5185.23) | -0.15 (-0.24--0.07) |
| Republic of Korea | 441.22 (378.9-506.56) | 462.22 (369.79-571.89) | 0.05 | 3492.03 (2998.83-4009.21) | 3987.11 (3189.87-4933.13) | 0.22 (0.11-0.34) |
| Republic of Latvia | 40.14 (33.14-48.45) | 28.13 (21.42-36.38) | -0.3 | 6193.87 (5113.15-7475.14) | 7218.57 (5498.02-9336.41) | -0.21 (-0.41-0) |
| Republic of Liberia | 39.96 (32.84-49.93) | 105.93 (81.08-136.87) | 1.65 | 7202.53 (5919.12-8998.37) | 7591.76 (5810.51-9808.8) | 0.43 (0.21-0.65) |
| Republic of Lithuania | 57.73 (48.34-68.79) | 46.93 (36.24-59.44) | -0.19 | 6261.91 (5243.84-7461.73) | 8191.06 (6325.87-10375.54) | -0.04 (-0.27-0.19) |
| Republic of Madagascar | 171.95 (140.43-212.15) | 508.94 (393.47-650.84) | 1.96 | 6384.66 (5214.05-7877.16) | 7026.3 (5432.09-8985.29) | 0.02 (-0.09-0.13) |
| Republic of Malawi | 132.86 (107.43-162.28) | 314.39 (244.6-404.8) | 1.37 | 5884.35 (4757.75-7187.07) | 6276.11 (4882.94-8081.02) | -0.2 (-0.33--0.07) |
| Republic of Maldives | 2.57 (2.15-3.16) | 5.87 (4.74-7.38) | 1.28 | 5407.58 (4520.97-6645.33) | 5095.42 (4117-6409.05) | -0.67 (-0.84--0.49) |
| Republic of Mali | 99.7 (81.74-123.86) | 282.11 (220.64-362.34) | 1.83 | 5195.83 (4259.49-6454.61) | 5181.65 (4052.62-6655.33) | -0.16 (-0.28--0.05) |
| Republic of Malta | 5.38 (4.4-6.66) | 6.09 (4.69-7.94) | 0.13 | 5677.88 (4635.54-7024.93) | 6463.67 (4976.36-8425.95) | 0.17 (-0.01-0.36) |
| Republic of Mauritius | 20.25 (16.78-24.27) | 23.09 (17.74-29.47) | 0.14 | 6778.98 (5616.74-8124.67) | 7293.97 (5606.49-9310.87) | -0.32 (-0.5--0.15) |
| Republic of Moldova | 60.05 (50.02-71.46) | 53 (40.97-68.44) | -0.12 | 5335.35 (4444.42-6349.4) | 6008.84 (4645.11-7758.91) | -0.24 (-0.46--0.01) |
| Republic of Mozambique | 201.12 (165.29-248) | 552.99 (428.41-726.66) | 1.75 | 6373.6 (5237.92-7859.27) | 7283.57 (5642.7-9571.04) | 0.08 (-0.02-0.18) |
| Republic of Namibia | 17.01 (14-20.94) | 43.79 (33.99-56.31) | 1.57 | 5015.36 (4128.62-6174.75) | 6621.47 (5139.94-8514.13) | 0.15 (-0.09-0.4) |
| Republic of Nauru | 0.1 (0.08-0.13) | 0.13 (0.1-0.17) | 0.3 | 4246.22 (3427.44-5310.17) | 4709.74 (3657.78-6059.01) | 0.1 (0.04-0.16) |
| Republic of Nicaragua | 43.25 (35.55-54) | 108.54 (82.38-139.18) | 1.51 | 4793.99 (3940.36-5984.94) | 5987.26 (4544.28-7677.48) | 0.2 (0.02-0.38) |
| Republic of Niue | 0.02 (0.02-0.03) | 0.02 (0.01-0.02) | 0 | 4340.73 (3546.35-5415.12) | 4864.26 (3790.37-6227.65) | 0.18 (0.13-0.23) |
| Republic of Palau | 0.18 (0.14-0.22) | 0.19 (0.15-0.25) | 0.06 | 4303.24 (3506.7-5335.24) | 5006.8 (3945.42-6545.21) | 0.26 (0.21-0.31) |
| Republic of Panama | 25.81 (20.96-32.52) | 57.79 (42.91-75.52) | 1.24 | 4217.76 (3425.17-5314.25) | 5406.24 (4014.59-7064.97) | 0.38 (0.17-0.58) |
| Republic of Paraguay | 49.88 (41.1-61.95) | 137.31 (104.65-180.82) | 1.75 | 5305.87 (4371.99-6589.35) | 7238.18 (5516.54-9531.9) | 0.45 (0.27-0.63) |
| Republic of Peru | 188.44 (154.57-232.32) | 419.43 (326.12-533.68) | 1.23 | 3468.88 (2845.33-4276.71) | 4360.47 (3390.43-5548.28) | 0.04 (-0.27-0.35) |
| Republic of Poland | 318.48 (274.66-370.76) | 354.24 (304.18-415.54) | 0.11 | 3390.44 (2923.9-3947.05) | 4012.66 (3445.55-4707.08) | -0.05 (-0.28-0.19) |
| Republic of Rwanda | 128.44 (106.16-161.79) | 286.24 (222.46-368.62) | 1.23 | 7916.21 (6542.9-9971.93) | 8128.17 (6317.08-10467.65) | -0.54 (-0.71--0.37) |
| Republic of San Marino | 0.45 (0.36-0.57) | 0.64 (0.47-0.86) | 0.42 | 7162.03 (5785.47-9220.16) | 8947.82 (6639.46-12010.67) | 0.3 (0.12-0.48) |
| Republic of Senegal | 88.03 (72.26-108.97) | 232.41 (182.45-302.26) | 1.64 | 5116.13 (4199.24-6332.7) | 5957.66 (4677.14-7748.36) | 0.2 (0.06-0.34) |
| Republic of Serbia | 111.49 (94.22-134.22) | 106.44 (83.57-135.57) | -0.05 | 4774.67 (4035.41-5748.34) | 5236.11 (4111.21-6669.34) | -0.22 (-0.39--0.05) |
| Republic of Seychelles | 0.62 (0.51-0.76) | 1.06 (0.84-1.34) | 0.71 | 3453.19 (2809.09-4219.89) | 4392.48 (3482.35-5522.99) | 0.4 (0.28-0.51) |
| Republic of Sierra Leone | 58.85 (48.2-72.8) | 141.59 (110.9-182.91) | 1.41 | 5861.84 (4800.5-7251.29) | 6225.99 (4876.54-8042.82) | 0.2 (0.14-0.25) |
| Republic of Singapore | 49.48 (43.01-57.12) | 50.16 (39.64-63.63) | 0.01 | 5293.72 (4601.53-6111.55) | 3448.16 (2725.13-4374.47) | -1.99 (-2.29--1.69) |
| Republic of Slovenia | 29.78 (25.1-35.32) | 25.15 (19.43-32.01) | -0.16 | 5984.68 (5044.36-7099.17) | 5978.4 (4620.33-7611.06) | -0.54 (-0.72--0.37) |
| Republic of South Africa | 615.82 (537.79-706.36) | 1277.53 (1098.76-1485.58) | 1.07 | 6379.37 (5571.01-7317.25) | 8248.65 (7094.41-9591.96) | 0.5 (0.27-0.72) |
| Republic of South Sudan | 82.51 (67.11-101.65) | 160.25 (124.28-207.3) | 0.94 | 6371.55 (5182.42-7849.33) | 6902.35 (5352.94-8928.99) | 0.16 (0.09-0.23) |
| Republic of Sudan | 324.2 (264.19-414.34) | 849.27 (648.21-1115.64) | 1.62 | 6924.55 (5642.73-8849.96) | 7522.99 (5741.97-9882.61) | -0.11 (-0.26-0.03) |
| Republic of Suriname | 6.58 (5.45-8.12) | 14.02 (10.45-18.29) | 1.13 | 6762.46 (5604.94-8350.49) | 9662.8 (7199.21-12606.06) | 0.43 (0.23-0.63) |
| Republic of Tajikistan | 49.72 (40.77-61.26) | 124.85 (97.7-159.25) | 1.51 | 4124.17 (3381.38-5081.16) | 4912.75 (3844.35-6266.15) | 0.07 (-0.1-0.25) |
| Republic of the Congo | 49.61 (39.93-62.86) | 139.33 (105.52-185.44) | 1.81 | 8801.66 (7083.35-11152.62) | 9724.38 (7364.8-12942.97) | 0.04 (-0.11-0.19) |
| Republic of the Gambia | 20.3 (16.63-25.38) | 58.24 (44.42-75.38) | 1.87 | 8945.86 (7327.75-11184.71) | 9470.39 (7223.38-12257.12) | -0.3 (-0.45--0.16) |
| Republic of the Marshall Islands | 0.37 (0.31-0.46) | 0.63 (0.5-0.78) | 0.7 | 3832.16 (3190.36-4709.24) | 4235.06 (3374.82-5256.59) | 0.1 (0.04-0.16) |
| Republic of the Niger | 102.17 (83.53-125.46) | 301.92 (238.39-387.32) | 1.96 | 5873.14 (4801.99-7212.34) | 5663.34 (4471.68-7265.26) | -0.1 (-0.14--0.06) |
| Republic of the Philippines | 627.34 (539.1-734.72) | 1362.93 (1170.52-1609.02) | 1.17 | 4042.31 (3473.72-4734.24) | 4649.58 (3993.17-5489.09) | 0.06 (-0.11-0.23) |
| Republic of the Union of Myanmar | 295.87 (236.44-363.15) | 535.79 (428.54-667.86) | 0.81 | 2838.85 (2268.6-3484.45) | 3544.08 (2834.63-4417.7) | 0.36 (0.2-0.52) |
| Republic of Trinidad and Tobago | 18.76 (15.32-22.45) | 26.02 (19.02-34.24) | 0.39 | 6098.05 (4980.69-7296.68) | 7655.8 (5597.61-10073.32) | -0.01 (-0.24-0.23) |
| Republic of Tunisia | 157.26 (127.5-198.66) | 315.46 (236.51-416.11) | 1.01 | 7691.54 (6235.92-9716.06) | 10281.82 (7708.41-13562.23) | 0.32 (0.09-0.55) |
| Republic of Turkey | 968.52 (852.08-1097.03) | 1774.24 (1345.76-2318.12) | 0.83 | 6799.41 (5981.94-7701.57) | 8194.53 (6215.54-10706.47) | 0.3 (0.16-0.44) |
| Republic of Uganda | 331.35 (272.08-415.83) | 980.32 (758.03-1274.24) | 1.96 | 8610.99 (7070.68-10806.45) | 9413.75 (7279.17-12236.19) | -0.46 (-0.75--0.18) |
| Republic of Uzbekistan | 219.04 (177.59-264.75) | 454.72 (356.51-582.01) | 1.08 | 4471.42 (3625.31-5404.45) | 5110 (4006.34-6540.44) | 0.09 (-0.03-0.21) |
| Republic of Vanuatu | 1.46 (1.21-1.78) | 3.45 (2.74-4.3) | 1.36 | 4176.23 (3445.68-5084.28) | 4401.06 (3489.35-5478.22) | -0.09 (-0.16--0.01) |
| Republic of Yemen | 224.33 (180.79-285.56) | 684.57 (520.79-891.96) | 2.05 | 8189.59 (6600.03-10425.17) | 8192.84 (6232.74-10674.89) | -0.07 (-0.13--0.01) |
| Republic of Zambia | 100.91 (82.87-124.62) | 307.91 (241.74-394.57) | 2.05 | 5486.86 (4505.88-6776.42) | 6259.83 (4914.45-8021.56) | 0.01 (-0.1-0.12) |
| Republic of Zimbabwe | 103.19 (84.4-128.24) | 207.36 (164.08-263.42) | 1.01 | 4291.72 (3510.31-5333.32) | 5106.52 (4040.66-6487.13) | 0.26 (0.11-0.4) |
| Romania | 232.1 (192.59-278.25) | 212.22 (166.33-268.5) | -0.09 | 4127.57 (3424.91-4948.27) | 5226.37 (4096.1-6612.44) | 0.26 (0.07-0.45) |
| Russian Federation | 1749.45 (1494.45-2067.74) | 2031.95 (1728.82-2388.73) | 0.16 | 4724.97 (4036.26-5584.63) | 5995.06 (5100.7-7047.7) | 0.21 (-0.04-0.45) |
| Saint Kitts and Nevis | 0.55 (0.43-0.72) | 1.11 (0.81-1.52) | 1.02 | 5504.67 (4345.32-7256.37) | 7088.05 (5174.62-9720.4) | 0.41 (0.29-0.54) |
| Saint Lucia | 1.54 (1.25-1.93) | 2.86 (2.16-3.8) | 0.86 | 4501.02 (3661.21-5673.08) | 6269.95 (4736.82-8308.69) | 0.5 (0.3-0.71) |
| Saint Vincent and the Grenadines | 1.17 (0.96-1.49) | 1.65 (1.25-2.16) | 0.41 | 4440.61 (3625.14-5641.2) | 5924.63 (4506.2-7771.92) | 0.48 (0.31-0.65) |
| Slovak Republic | 59.57 (49.73-71.16) | 70.45 (54.78-92.13) | 0.18 | 4490.66 (3749.24-5364.65) | 5581.35 (4340.14-7299.19) | 0.05 (-0.19-0.3) |
| Socialist Republic of Viet Nam | 638.71 (529.93-772.42) | 1086.53 (876.45-1343.94) | 0.7 | 3742.93 (3105.48-4526.49) | 4253.95 (3431.45-5261.76) | 0.04 (-0.05-0.14) |
| Solomon Islands | 3.08 (2.55-3.84) | 7.62 (6.04-9.62) | 1.47 | 4137.67 (3421.56-5164.7) | 4445.55 (3523.19-5610.69) | 0.02 (-0.05-0.08) |
| State of Eritrea | 52.04 (42.95-63.3) | 116.6 (91.3-149.07) | 1.24 | 6598.64 (5446.02-8026.89) | 7044.31 (5516.08-9006.26) | 0 (-0.08-0.09) |
| State of Israel | 90.68 (74.5-112.04) | 184.41 (141.03-241.04) | 1.03 | 7433.08 (6107.33-9184.65) | 8290.31 (6340.07-10836.06) | -0.27 (-0.45--0.09) |
| State of Kuwait | 28.46 (23.03-35.75) | 106.04 (80.5-140.36) | 2.73 | 6866.81 (5556.38-8626.81) | 7254.47 (5507.41-9602.42) | 0.06 (-0.03-0.14) |
| State of Libya | 61.51 (49.72-78.13) | 160.74 (125.01-208.14) | 1.61 | 6753.53 (5459.02-8579.32) | 8080.83 (6284.8-10463.73) | 0.38 (0.27-0.5) |
| State of Qatar | 6.06 (4.96-7.44) | 44.5 (34-58.85) | 6.34 | 7664.9 (6269.96-9412.55) | 8021.93 (6127.86-10606.95) | -0.18 (-0.32--0.04) |
| Sultanate of Oman | 22.04 (17.87-27.44) | 80.51 (61.63-107.3) | 2.65 | 6501.93 (5272.69-8094.04) | 7928.14 (6069.12-10566.72) | 0.35 (0.15-0.54) |
| Swiss Confederation | 140.27 (118.58-164.47) | 155.97 (118.68-202.07) | 0.11 | 7968.71 (6736.43-9343.81) | 7971.45 (6065.91-10327.59) | -0.6 (-0.98--0.22) |
| Syrian Arab Republic | 175.26 (141.42-223.25) | 296.11 (220.06-395.71) | 0.69 | 6355.77 (5128.68-8096.15) | 7611.24 (5656.35-10171.27) | 0.3 (0.14-0.46) |
| Taiwan (Province of China) | 180.05 (146.81-221.21) | 230.94 (182.92-285.23) | 0.28 | 3272.24 (2668.23-4020.32) | 4110.92 (3256.09-5077.43) | 0.67 (0.62-0.72) |
| Togolese Republic | 50.5 (41.62-62.31) | 138.06 (106.08-175.42) | 1.73 | 5918.08 (4877.34-7302.85) | 6402.49 (4919.55-8135.02) | 0.15 (0.08-0.21) |
| Tokelau | 0.02 (0.01-0.02) | 0.02 (0.01-0.02) | 0 | 4270.76 (3488.07-5298.79) | 4810.87 (3787.1-6261) | 0.16 (0.09-0.23) |
| Turkmenistan | 38.73 (31.95-46.87) | 63.51 (49.69-79.69) | 0.64 | 4382.49 (3615.01-5302.55) | 5054.35 (3954.14-6341.62) | 0.07 (-0.07-0.2) |
| Tuvalu | 0.11 (0.09-0.13) | 0.14 (0.11-0.18) | 0.27 | 4368.22 (3572.83-5456.97) | 4692.62 (3653.85-6130.71) | -0.02 (-0.08-0.03) |
| Ukraine | 779.75 (660.52-932.35) | 764.47 (596.98-965.08) | -0.02 | 6159.94 (5217.97-7365.46) | 7565.11 (5907.62-9550.26) | -0.19 (-0.39-0.01) |
| Union of the Comoros | 6.1 (4.97-7.52) | 12.89 (10.11-16.75) | 1.11 | 5791.17 (4721.33-7133.78) | 6601.1 (5180.67-8577.94) | 0.11 (0-0.22) |
| United Arab Emirates | 22.85 (18.63-28.71) | 134.55 (102.59-176.09) | 4.89 | 6675.26 (5442.02-8387.61) | 7804.7 (5951-10214.07) | 0.1 (-0.11-0.3) |
| United Kingdom of Great Britain and Northern Ireland | 1112.44 (955.35-1317.51) | 1309.06 (1107.83-1561.94) | 0.18 | 7838.76 (6731.82-9283.74) | 8533.92 (7222.06-10182.46) | -0.09 (-0.38-0.2) |
| United Mexican States | 951.3 (820.77-1118.84) | 2723.62 (2312.55-3207.8) | 1.86 | 4345.2 (3749-5110.46) | 7772.01 (6598.98-9153.64) | 1.73 (1.49-1.97) |
| United Republic of Tanzania | 381.73 (311.54-464.81) | 1027.56 (807.22-1328.41) | 1.69 | 6361.91 (5192.17-7746.56) | 6915.19 (5432.39-8939.85) | -0.01 (-0.1-0.09) |
| United States of America | 4777.65 (4184.44-5420.18) | 8040.51 (7049.91-9157.39) | 0.68 | 7131.37 (6245.93-8090.46) | 10619.56 (9311.22-12094.68) | 0.48 (0.25-0.71) |
| United States Virgin Islands | 1.38 (1.12-1.72) | 1.01 (0.79-1.3) | -0.27 | 4804.13 (3879.98-5966.73) | 5891.83 (4564.88-7547.92) | 0.3 (0.15-0.45) |

**Abbreviations: WCBA, Women of Childbearing Age; EAPC, Estimated Annual Percentage Change; UI, Uncertainty Intervals CI, Confidence Intervals.**

# **Table S5. The DALYs of depression cases and rates among WCBA in 1990 and 2021 across 204 countries, and the trends from 1990 to 2021.**

| location | DALY cases | | | DALY rates | | |
| --- | --- | --- | --- | --- | --- | --- |
|  | 1990_thousands(95% UI) | 2021_thousands(95% UI) | percentage change(100%) | 1990_per 100 000(95% UI) | 2021_per 100 000(95% UI) | EAPC(95% CI) |
| American Samoa | 0.07 (0.04-0.09) | 0.07 (0.05-0.1) | 0 | 549.67 (359.16-769.92) | 614.74 (398.46-884.26) | 0.1 (0.01-0.19) |
| Antigua and Barbuda | 0.13 (0.08-0.19) | 0.25 (0.16-0.4) | 0.92 | 798.48 (502.72-1177.52) | 1039.7 (643.21-1635.19) | 0.36 (0.13-0.59) |
| Arab Republic of Egypt | 144.32 (94.22-209.14) | 353.48 (218.89-522.15) | 1.45 | 1100.57 (718.54-1594.87) | 1362.27 (843.59-2012.33) | 0.3 (0.1-0.5) |
| Argentine Republic | 67.9 (44.66-96.26) | 120.72 (76.35-171.86) | 0.78 | 845.93 (556.38-1199.2) | 1015.66 (642.32-1445.85) | -0.08 (-0.32-0.16) |
| Australia | 63.36 (43.88-85.15) | 95.82 (62.82-137.63) | 0.51 | 1419.61 (983.07-1907.66) | 1590.49 (1042.76-2284.57) | 0.2 (0.03-0.38) |
| Barbados | 0.55 (0.36-0.8) | 0.78 (0.47-1.18) | 0.42 | 807.04 (522.6-1167.41) | 1097.65 (662.87-1660.61) | 0.4 (0.19-0.61) |
| Belize | 0.33 (0.2-0.48) | 1.25 (0.8-1.81) | 2.79 | 787.13 (485.85-1151.85) | 1033.36 (665.94-1500.93) | 0.48 (0.29-0.67) |
| Bermuda | 0.17 (0.11-0.24) | 0.15 (0.09-0.23) | -0.12 | 997.55 (647.29-1393.26) | 1098.17 (657.92-1712.75) | -0.33 (-0.56--0.1) |
| Bolivarian Republic of Venezuela | 38.56 (24.5-56) | 64.54 (39.1-97.24) | 0.67 | 796.68 (506.19-1156.85) | 940.32 (569.68-1416.75) | 0.22 (0.11-0.34) |
| Bosnia and Herzegovina | 10.41 (6.87-14.53) | 7.07 (4.47-10.78) | -0.32 | 893.82 (589.78-1248.37) | 966.66 (611.6-1473.25) | -0.66 (-0.93--0.39) |
| Brunei Darussalam | 0.32 (0.21-0.47) | 0.69 (0.42-1.02) | 1.16 | 478.85 (308.5-700.98) | 548.68 (333.69-817.04) | 0.12 (-0.09-0.32) |
| Burkina Faso | 20.15 (13.2-28.79) | 53.99 (34.01-82.68) | 1.68 | 962.68 (630.75-1375.47) | 982.91 (619.23-1505.21) | 0.36 (0.23-0.49) |
| Canada | 78.53 (54.53-105.77) | 116.42 (73.82-170.87) | 0.48 | 1067.61 (741.31-1437.85) | 1403.65 (890.04-2060.16) | 0.03 (-0.25-0.31) |
| Central African Republic | 10.38 (6.66-14.9) | 23.73 (15.04-34.65) | 1.29 | 1619.67 (1038.67-2324.52) | 1714.96 (1087.16-2504.74) | 0.05 (-0.06-0.16) |
| Commonwealth of Dominica | 0.13 (0.09-0.19) | 0.17 (0.1-0.27) | 0.31 | 781.9 (499.06-1137.46) | 1039.12 (622.44-1640.85) | 0.36 (0.14-0.58) |
| Commonwealth of the Bahamas | 0.58 (0.37-0.83) | 1.15 (0.69-1.72) | 0.98 | 798.46 (515.31-1150.44) | 1063.98 (637.83-1594.21) | 0.39 (0.15-0.63) |
| Cook Islands | 0.03 (0.02-0.05) | 0.03 (0.02-0.05) | 0 | 694.48 (451.67-1020.26) | 795.55 (501.02-1211.63) | 0.19 (0.13-0.25) |
| Czech Republic | 22.58 (15.05-31.5) | 22.04 (14.08-31.81) | -0.02 | 876.97 (584.66-1223.67) | 960.71 (613.84-1386.92) | -0.44 (-0.68--0.19) |
| Democratic People's Republic of Korea | 35.44 (23.25-49.04) | 40.63 (26.19-58.07) | 0.15 | 625.03 (410-864.71) | 616.52 (397.48-881.13) | -0.2 (-0.25--0.15) |
| Democratic Republic of Sao Tome and Principe | 0.23 (0.15-0.33) | 0.56 (0.35-0.82) | 1.43 | 890.88 (588.52-1274.01) | 995.94 (621.55-1465.06) | 0.25 (0.14-0.36) |
| Democratic Republic of the Congo | 128.26 (84.6-185.3) | 333.56 (210.75-475.37) | 1.6 | 1502.77 (991.22-2171.12) | 1565.46 (989.08-2231.02) | 0.01 (-0.09-0.11) |
| Democratic Republic of Timor-Leste | 1.29 (0.86-1.81) | 2.38 (1.47-3.63) | 0.84 | 693.6 (458.82-971.23) | 688.54 (424.7-1047.57) | -0.62 (-0.82--0.42) |
| Democratic Socialist Republic of Sri Lanka | 38.77 (26.62-54.15) | 45.45 (30.56-65.3) | 0.17 | 844.49 (579.74-1179.43) | 804.86 (541.15-1156.35) | -0.92 (-1.22--0.61) |
| Dominican Republic | 19.09 (11.99-27.84) | 35.65 (21.08-55.19) | 0.87 | 1011.06 (635.26-1474.38) | 1230.74 (727.74-1905.06) | 0.26 (0.07-0.45) |
| Eastern Republic of Uruguay | 6.84 (4.54-9.7) | 11.07 (6.89-16.34) | 0.62 | 910.83 (604.19-1291.22) | 1329.51 (827.44-1963.5) | 0.88 (0.67-1.09) |
| Federal Democratic Republic of Ethiopia | 121.02 (81.04-169.05) | 302.26 (202.1-428.22) | 1.5 | 1072.53 (718.21-1498.17) | 1090.44 (729.1-1544.84) | -0.33 (-0.54--0.13) |
| Federal Democratic Republic of Nepal | 47.15 (31.31-66.87) | 135.81 (85.28-208.34) | 1.88 | 1032.21 (685.55-1463.99) | 1497.15 (940.07-2296.71) | 0.72 (0.49-0.95) |
| Federal Republic of Germany | 176.49 (122.26-237.28) | 216.61 (138.66-315.85) | 0.23 | 908.07 (629.06-1220.89) | 1271.89 (814.19-1854.57) | 1.05 (0.89-1.21) |
| Federal Republic of Nigeria | 185.47 (123.38-258.08) | 470.96 (314.82-655.62) | 1.54 | 915.82 (609.2-1274.36) | 820.2 (548.26-1141.78) | -0.55 (-0.72--0.37) |
| Federal Republic of Somalia | 19.27 (12.6-27.09) | 67.03 (41.19-98.54) | 2.48 | 1137.58 (743.89-1599.3) | 1385.93 (851.59-2037.41) | 0.08 (-0.08-0.25) |
| Federated States of Micronesia | 0.15 (0.1-0.21) | 0.17 (0.11-0.25) | 0.13 | 632.2 (413-895.8) | 664.86 (428.34-968.3) | -0.11 (-0.2--0.02) |
| Federative Republic of Brazil | 468.48 (316.29-644.26) | 851.09 (565.62-1166.21) | 0.82 | 1202.75 (812.03-1654.03) | 1449.51 (963.31-1986.19) | -0.3 (-0.65-0.06) |
| French Republic | 211.81 (149.09-284.73) | 219.87 (142.9-319.7) | 0.04 | 1463.58 (1030.19-1967.45) | 1548.65 (1006.53-2251.85) | -0.28 (-0.48--0.08) |
| Gabonese Republic | 3.25 (2.08-4.67) | 8.18 (5.05-12.55) | 1.52 | 1467.56 (939.58-2108.69) | 1670.13 (1030.56-2564.26) | -0.01 (-0.15-0.12) |
| Georgia | 9.96 (6.6-14.32) | 7.59 (4.78-11.14) | -0.24 | 725.76 (480.72-1043.44) | 963.87 (607.09-1416.14) | 0.33 (0.13-0.53) |
| Grand Duchy of Luxembourg | 1.22 (0.81-1.68) | 1.81 (1.16-2.61) | 0.48 | 1259.5 (836.5-1733.88) | 1165.76 (748.04-1678.6) | -0.8 (-1.03--0.57) |
| Greenland | 0.4 (0.28-0.57) | 0.38 (0.25-0.58) | -0.05 | 2740.72 (1880.87-3881.59) | 3014.3 (1956.14-4524.11) | -0.38 (-0.61--0.15) |
| Grenada | 0.16 (0.1-0.22) | 0.27 (0.17-0.41) | 0.69 | 798.13 (497.97-1148.59) | 1042.39 (659.62-1594.19) | 0.38 (0.2-0.56) |
| Guam | 0.23 (0.15-0.32) | 0.28 (0.19-0.41) | 0.22 | 658.6 (441.87-926.05) | 788.78 (520.67-1134.25) | 0.21 (0.05-0.36) |
| Hashemite Kingdom of Jordan | 11.18 (7.31-16.22) | 44.56 (27.67-69.1) | 2.99 | 1340.65 (876.12-1944.78) | 1437.88 (892.81-2229.89) | -0.17 (-0.3--0.03) |
| Hellenic Republic | 41.03 (25.64-59.4) | 44.58 (26.94-67.66) | 0.09 | 1628.18 (1017.73-2357.6) | 2056.87 (1242.93-3121.65) | -0.01 (-0.38-0.37) |
| Hungary | 23.35 (15.65-32.63) | 18.84 (11.73-27.33) | -0.19 | 921.23 (617.45-1287.21) | 880.71 (548.64-1277.59) | -0.85 (-1.09--0.61) |
| Independent State of Papua New Guinea | 6.87 (4.54-9.67) | 18.75 (11.48-27.28) | 1.73 | 713.33 (471.53-1003.47) | 716.79 (438.73-1042.58) | -0.15 (-0.19--0.11) |
| Independent State of Samoa | 0.22 (0.15-0.32) | 0.31 (0.2-0.47) | 0.41 | 608.89 (404.19-866.65) | 645.66 (406.95-955.82) | -0.14 (-0.23--0.04) |
| Ireland | 10.92 (7.39-15.04) | 19.87 (12.79-29.44) | 0.82 | 1236.46 (836.92-1702.64) | 1703.06 (1096.33-2523.24) | 0.41 (0.19-0.64) |
| Islamic Republic of Afghanistan | 30.64 (19.8-42.85) | 120.27 (75.52-178.09) | 2.93 | 1392.38 (899.9-1947.64) | 1671.5 (1049.55-2475.06) | 0.19 (0.05-0.34) |
| Islamic Republic of Iran | 194.45 (128.77-278.46) | 430.88 (282.96-621.87) | 1.22 | 1537.5 (1018.18-2201.72) | 1853.6 (1217.27-2675.24) | 0.77 (0.55-0.99) |
| Islamic Republic of Mauritania | 3.7 (2.47-5.29) | 8.99 (5.71-13.61) | 1.43 | 787.88 (524.92-1125.04) | 837.88 (531.49-1268.29) | -0.05 (-0.18-0.08) |
| Islamic Republic of Pakistan | 231.46 (152.52-327.64) | 668.29 (432.46-955.79) | 1.89 | 978.48 (644.79-1385.09) | 1103.25 (713.93-1577.88) | 0.15 (-0.03-0.33) |
| Jamaica | 4.71 (3.02-7) | 8.27 (5.07-12.79) | 0.76 | 790.29 (507.13-1174.75) | 1070.84 (656.66-1656.6) | 0.36 (0.12-0.61) |
| Japan | 194.26 (133.01-266.3) | 198.47 (135.65-269.7) | 0.02 | 605.1 (414.33-829.51) | 798.17 (545.5-1084.59) | 0.45 (0.17-0.73) |
| Kingdom of Bahrain | 1.75 (1.15-2.5) | 5.38 (3.38-8.05) | 2.07 | 1519.33 (993.63-2167.26) | 1648.98 (1037.53-2468.65) | -0.33 (-0.51--0.15) |
| Kingdom of Belgium | 24.7 (17.56-32.9) | 33.69 (20.61-47.98) | 0.36 | 1014.62 (721.33-1351.35) | 1359.69 (831.69-1936.46) | 0.82 (0.63-1.02) |
| Kingdom of Bhutan | 1.34 (0.88-1.91) | 2 (1.23-3.04) | 0.49 | 937.21 (617.37-1331.53) | 965.55 (595.94-1468.34) | -0.04 (-0.18-0.11) |
| Kingdom of Cambodia | 18.75 (12.51-26.4) | 34.86 (22.84-51.16) | 0.86 | 748.85 (499.67-1054.36) | 771.46 (505.45-1132.18) | -0.62 (-0.8--0.44) |
| Kingdom of Denmark | 19.49 (13.24-27.01) | 16.66 (10.5-24.45) | -0.15 | 1492.64 (1014.38-2068.87) | 1319.32 (831.45-1936.06) | -0.96 (-1.23--0.69) |
| Kingdom of Eswatini | 1.88 (1.24-2.68) | 4.79 (2.94-7.19) | 1.55 | 962.84 (635.86-1375.57) | 1520.27 (932.87-2280.99) | 0.6 (0.32-0.88) |
| Kingdom of Lesotho | 5.89 (3.86-8.44) | 10.02 (6.21-14.62) | 0.7 | 1556.04 (1020.71-2230.28) | 1977.19 (1225.3-2883.87) | 0.28 (0.02-0.54) |
| Kingdom of Morocco | 92.22 (59.66-131.94) | 169.17 (108.4-247.83) | 0.83 | 1464.43 (947.38-2095.09) | 1746.54 (1119.15-2558.69) | 0.13 (-0.07-0.34) |
| Kingdom of Norway | 11.91 (7.86-16.34) | 16.89 (11.08-23.73) | 0.42 | 1128.86 (744.28-1548.5) | 1394.31 (914.46-1959.75) | 0.26 (0.1-0.43) |
| Kingdom of Saudi Arabia | 37.89 (23.95-55.61) | 142.57 (89.18-212.81) | 2.76 | 1161.94 (734.43-1705.41) | 1405.2 (878.94-2097.45) | 0.49 (0.33-0.65) |
| Kingdom of Spain | 124.07 (86.57-167.88) | 187.73 (124.72-272.32) | 0.51 | 1288.32 (898.84-1743.16) | 1895.68 (1259.37-2749.84) | 1.39 (1.01-1.77) |
| Kingdom of Sweden | 31.85 (21.89-43.37) | 40.2 (26.92-56.83) | 0.26 | 1551.58 (1066.59-2112.55) | 1829.79 (1225.48-2586.67) | 0.07 (-0.09-0.23) |
| Kingdom of Thailand | 102.4 (68.21-144.2) | 117.59 (74.72-171.93) | 0.15 | 640.41 (426.58-901.78) | 721.38 (458.35-1054.68) | 0.02 (-0.07-0.11) |
| Kingdom of the Netherlands | 47.18 (33.1-63.22) | 51.45 (32.56-76.88) | 0.09 | 1190.82 (835.51-1595.79) | 1402.13 (887.4-2095.16) | -0.11 (-0.36-0.15) |
| Kingdom of Tonga | 0.13 (0.09-0.19) | 0.16 (0.11-0.24) | 0.23 | 590.62 (390.17-835.17) | 636.75 (423.3-931.69) | -0.04 (-0.13-0.05) |
| Kyrgyz Republic | 8.45 (5.66-11.91) | 17.33 (10.67-25.59) | 1.05 | 806.6 (539.8-1135.96) | 1005.24 (618.59-1484.45) | 0.18 (-0.02-0.38) |
| Lao People's Democratic Republic | 5.89 (3.95-8.38) | 12.06 (7.58-17.32) | 1.05 | 608.94 (407.92-866.42) | 607.79 (382.18-873.06) | -0.38 (-0.63--0.14) |
| Lebanese Republic | 9.09 (5.99-13.14) | 28.65 (17.61-42.99) | 2.15 | 1213.3 (799.77-1753.96) | 1931.23 (1186.7-2897.64) | 0.53 (0.29-0.77) |
| Malaysia | 30.05 (20.02-42.47) | 68.43 (43.38-101.3) | 1.28 | 670.61 (446.84-947.98) | 812.11 (514.78-1202.13) | 0.96 (0.73-1.19) |
| Mongolia | 5.13 (3.31-7.29) | 8.93 (5.7-13.07) | 0.74 | 1002.01 (646.53-1424.21) | 1058.03 (675.6-1548.44) | -0.03 (-0.07-0.02) |
| Montenegro | 1.16 (0.76-1.64) | 1.44 (0.92-2.15) | 0.24 | 745.51 (486.21-1050.48) | 999.32 (637.16-1491.41) | 0.2 (-0.03-0.42) |
| New Zealand | 10.58 (7-14.93) | 15.09 (9.97-21.46) | 0.43 | 1170.23 (773.89-1652.08) | 1262.64 (834.75-1795.96) | 0.09 (0.04-0.15) |
| North Macedonia | 3.51 (2.35-4.89) | 4.93 (3.17-7.05) | 0.4 | 690.43 (461.35-960.3) | 929.74 (597.33-1329.2) | 0.09 (-0.21-0.39) |
| Northern Mariana Islands | 0.07 (0.05-0.11) | 0.08 (0.05-0.12) | 0.14 | 532.36 (352.41-765.56) | 685.05 (445.24-1011.93) | 0.39 (0.25-0.53) |
| Palestine | 7.12 (4.61-10.4) | 24.67 (15-37.22) | 2.46 | 1610.83 (1042.41-2352.09) | 1894.27 (1152.18-2858.48) | 0.1 (-0.09-0.28) |
| People's Democratic Republic of Algeria | 69.75 (44.56-101.38) | 154.77 (91.94-245.91) | 1.22 | 1200.3 (766.86-1744.58) | 1378.98 (819.19-2191.04) | 0.22 (0.06-0.38) |
| People's Republic of Bangladesh | 323.99 (211.65-457.94) | 697.25 (423.87-1035.42) | 1.15 | 1315.97 (859.68-1860.06) | 1515.91 (921.56-2251.15) | 0.12 (-0.04-0.27) |
| People's Republic of China | 2428.8 (1646.12-3352.84) | 1934.53 (1325.52-2625.49) | -0.2 | 753.79 (510.88-1040.57) | 607.01 (415.92-823.82) | -0.84 (-1.05--0.63) |
| Plurinational State of Bolivia | 14.97 (9.96-21.48) | 40.94 (25.04-60.92) | 1.73 | 978.43 (651.24-1403.89) | 1313 (803.08-1953.8) | 0.13 (-0.24-0.5) |
| Portuguese Republic | 43.77 (29.01-60.77) | 45.48 (27.74-67.71) | 0.04 | 1730.04 (1146.76-2402.16) | 1953.71 (1191.8-2908.59) | -0.42 (-0.74--0.1) |
| Principality of Andorra | 0.18 (0.12-0.25) | 0.29 (0.17-0.44) | 0.61 | 1186.72 (781.87-1688.25) | 1429.67 (869.26-2191.19) | 0.13 (-0.1-0.36) |
| Principality of Monaco | 0.09 (0.06-0.14) | 0.12 (0.07-0.17) | 0.33 | 1316.05 (849.54-1960.96) | 1637.25 (977.79-2445.53) | 0.18 (0.02-0.35) |
| Puerto Rico | 6.43 (4.3-9.19) | 6.17 (3.71-9.14) | -0.04 | 671.33 (448.8-959.52) | 822.88 (494.68-1219.53) | 0.21 (-0.01-0.42) |
| Republic of Albania | 5.15 (3.43-7.32) | 5.32 (3.44-8.09) | 0.03 | 616.79 (410.7-876.15) | 866.77 (560.23-1318.48) | 0.44 (0.23-0.65) |
| Republic of Angola | 37.71 (24.6-53.89) | 138.35 (85.53-205.04) | 2.67 | 1637.93 (1068.5-2340.89) | 1802.59 (1114.35-2671.56) | 0.01 (-0.11-0.13) |
| Republic of Armenia | 5.6 (3.7-8.11) | 6.63 (4.18-10.11) | 0.18 | 646.1 (427.39-935.87) | 899.94 (567.89-1371.63) | 0.49 (0.24-0.73) |
| Republic of Austria | 22.35 (14.97-30.73) | 22.38 (13.97-32.84) | 0 | 1129.2 (756.53-1552.7) | 1133.25 (707.68-1663.17) | -0.6 (-0.86--0.34) |
| Republic of Azerbaijan | 11.86 (7.74-17.08) | 23.48 (14.32-35.07) | 0.98 | 632.21 (412.81-911.05) | 856.67 (522.47-1279.48) | 0.44 (0.21-0.67) |
| Republic of Belarus | 24.94 (16.77-35.31) | 31.92 (19.78-46.5) | 0.28 | 984.52 (661.98-1393.44) | 1505.13 (932.66-2192.77) | 0.4 (0.15-0.65) |
| Republic of Benin | 10.84 (7.06-15.57) | 35.11 (22.08-53.73) | 2.24 | 989.44 (644.18-1421.07) | 1082.2 (680.58-1656.1) | 0.09 (0.01-0.16) |
| Republic of Botswana | 3.11 (2.07-4.43) | 9.08 (5.65-13.17) | 1.92 | 965.38 (642.73-1375.63) | 1333.88 (830.92-1935.66) | 0.36 (0.2-0.53) |
| Republic of Bulgaria | 15.97 (10.72-22.28) | 14.23 (8.8-20.53) | -0.11 | 770.97 (517.66-1076.01) | 998.68 (617.98-1441.55) | -0.21 (-0.51-0.09) |
| Republic of Burundi | 15.95 (10.47-22.62) | 38.41 (23.39-58.31) | 1.41 | 1266.37 (831.04-1795.79) | 1228.23 (747.86-1864.41) | -0.67 (-0.87--0.48) |
| Republic of Cabo Verde | 0.75 (0.49-1.08) | 1.99 (1.26-3) | 1.65 | 950.52 (623.21-1364.93) | 1323.29 (840.13-1998.42) | 0.57 (0.39-0.74) |
| Republic of Cameroon | 24.53 (16.22-34.9) | 88.67 (55.33-130.53) | 2.61 | 1031.05 (681.7-1466.63) | 1132.04 (706.4-1666.52) | 0.11 (0.04-0.18) |
| Republic of Chad | 15.99 (10.5-23.08) | 48.03 (30.05-72.93) | 2 | 1202.91 (789.67-1735.79) | 1241.72 (776.85-1885.58) | 0.03 (-0.04-0.11) |
| Republic of Chile | 57.35 (40.01-78.12) | 79.17 (49.34-116.56) | 0.38 | 1586.6 (1107.04-2161.29) | 1680.87 (1047.5-2474.57) | -0.42 (-0.67--0.18) |
| Republic of Colombia | 49.36 (32.62-69.85) | 72.15 (44.95-107.86) | 0.46 | 568.35 (375.63-804.25) | 550.72 (343.11-823.36) | -0.86 (-1.24--0.49) |
| Republic of Costa Rica | 6.21 (3.95-9.06) | 14.25 (8.92-21.41) | 1.29 | 798.8 (507.5-1165.35) | 1100.55 (688.68-1653.79) | 0.56 (0.39-0.74) |
| Republic of Côte d'Ivoire | 23.66 (15.92-33.91) | 61.79 (38.66-89.63) | 1.61 | 864.55 (581.89-1239.1) | 927.89 (580.54-1346.01) | 0.12 (0.04-0.2) |
| Republic of Croatia | 10.74 (7.25-14.72) | 8.15 (5.33-11.86) | -0.24 | 894.2 (603.92-1225.73) | 908.62 (594.61-1321.66) | -0.62 (-0.85--0.38) |
| Republic of Cuba | 46.86 (31.94-67.44) | 30.8 (20.33-45.36) | -0.34 | 1529.41 (1042.38-2200.8) | 1237.43 (816.68-1822.37) | -1.57 (-1.89--1.25) |
| Republic of Cyprus | 1.96 (1.26-2.83) | 4.17 (2.54-6.33) | 1.13 | 993.19 (639.08-1430.21) | 1168.89 (712.25-1771.25) | 0.17 (-0.02-0.36) |
| Republic of Djibouti | 0.98 (0.63-1.42) | 3.84 (2.36-5.64) | 2.92 | 1000.39 (639.84-1448.87) | 1196.44 (735.41-1754.44) | 0.32 (0.2-0.45) |
| Republic of Ecuador | 22.43 (14.77-31.95) | 55.49 (33.94-81.37) | 1.47 | 889.4 (585.47-1266.75) | 1176.75 (719.78-1725.56) | 0.29 (0-0.58) |
| Republic of El Salvador | 15.01 (9.82-21.43) | 21.55 (13.04-33.16) | 0.44 | 1144.99 (749.34-1634.74) | 1213.27 (734.23-1866.57) | -0.35 (-0.58--0.12) |
| Republic of Equatorial Guinea | 1.62 (1.04-2.32) | 6.41 (4.09-9.75) | 2.96 | 1639.5 (1054.63-2352.79) | 1759.16 (1121.2-2674.8) | 0.09 (-0.03-0.21) |
| Republic of Estonia | 4.82 (3.22-6.73) | 3.46 (2.22-5.04) | -0.28 | 1265.35 (846.29-1765.41) | 1242.43 (798.15-1811.24) | -1.09 (-1.35--0.82) |
| Republic of Fiji | 1.24 (0.82-1.74) | 1.71 (1.11-2.59) | 0.38 | 632.34 (419.26-887.63) | 750.74 (486.97-1131.91) | 0.08 (-0.05-0.2) |
| Republic of Finland | 20.51 (13.89-28.37) | 19.22 (12.85-28.02) | -0.06 | 1623.25 (1099.63-2245.79) | 1690.72 (1130.43-2465.61) | -0.54 (-0.77--0.31) |
| Republic of Ghana | 35.15 (22.39-50.89) | 101.59 (65.28-158.31) | 1.89 | 996.57 (634.96-1443.01) | 1112.72 (715-1733.9) | 0.06 (-0.05-0.17) |
| Republic of Guatemala | 17.49 (11.15-25.3) | 54.11 (33.7-84.36) | 2.09 | 952.2 (607.03-1377.07) | 1231 (766.77-1919.4) | 0.21 (-0.02-0.43) |
| Republic of Guinea | 14.14 (9.06-19.96) | 38.02 (23.88-57.31) | 1.69 | 1036.87 (664.26-1463.22) | 1145.58 (719.52-1726.89) | 0.06 (-0.06-0.18) |
| Republic of Guinea-Bissau | 2.42 (1.55-3.49) | 6.16 (3.82-9.3) | 1.55 | 1039.08 (664.03-1494.46) | 1172.72 (727.93-1769.53) | 0.2 (0.09-0.31) |
| Republic of Guyana | 2.6 (1.73-3.73) | 3.87 (2.42-5.77) | 0.49 | 1274.5 (847.1-1824.76) | 1904.6 (1190.54-2839.01) | 0.64 (0.45-0.84) |
| Republic of Haiti | 15.64 (10.08-22.46) | 42.18 (25.99-63.43) | 1.7 | 1016.24 (654.92-1459.74) | 1191.63 (734.07-1791.82) | 0.06 (-0.11-0.23) |
| Republic of Honduras | 7.9 (5.12-11.57) | 29.9 (17.75-44.46) | 2.78 | 749.2 (484.83-1096.97) | 1053.46 (625.3-1566.35) | 0.47 (0.22-0.72) |
| Republic of Iceland | 0.68 (0.45-0.95) | 0.81 (0.51-1.17) | 0.19 | 1043.65 (691.72-1465.45) | 1014.31 (645.86-1470.01) | -0.43 (-0.6--0.26) |
| Republic of India | 2041.89 (1375.22-2816.47) | 3959.61 (2690.44-5431.69) | 0.94 | 1011.3 (681.11-1394.93) | 1046.72 (711.21-1435.86) | -0.97 (-1.29--0.64) |
| Republic of Indonesia | 245.9 (167.96-340.74) | 500.18 (338.34-700.43) | 1.03 | 513.09 (350.46-710.99) | 664.3 (449.36-930.26) | 0.37 (0.19-0.55) |
| Republic of Iraq | 46.85 (30.92-65.98) | 134.95 (85.24-201.58) | 1.88 | 1154.57 (761.91-1626.13) | 1280.08 (808.49-1912.06) | 0.27 (-0.17-0.72) |
| Republic of Italy | 170.63 (113.14-240.75) | 182.08 (119.57-259.04) | 0.07 | 1193.83 (791.6-1684.44) | 1500.62 (985.49-2134.96) | -0.22 (-0.53-0.09) |
| Republic of Kazakhstan | 32.68 (21.73-45.22) | 47.12 (31.38-69.56) | 0.44 | 794.09 (527.98-1098.87) | 994.89 (662.67-1468.83) | 0.54 (0.38-0.69) |
| Republic of Kenya | 55.83 (37.92-76.47) | 152.29 (101.26-210.88) | 1.73 | 1077.92 (732.11-1476.36) | 1147.38 (762.93-1588.85) | -0.12 (-0.26-0.02) |
| Republic of Kiribati | 0.12 (0.08-0.17) | 0.21 (0.14-0.31) | 0.75 | 654.93 (437.44-913.39) | 658.18 (438.2-958.35) | -0.24 (-0.34--0.13) |
| Republic of Korea | 76.94 (51.71-104.99) | 81.84 (52.36-117.69) | 0.06 | 608.98 (409.28-830.98) | 705.97 (451.66-1015.22) | 0.25 (0.11-0.38) |
| Republic of Latvia | 7.09 (4.74-9.78) | 5.05 (3.08-7.46) | -0.29 | 1094.59 (730.93-1509.62) | 1296.62 (790.94-1913.18) | -0.27 (-0.51--0.03) |
| Republic of Liberia | 6.87 (4.51-9.85) | 18.44 (11.28-28.13) | 1.68 | 1238.87 (813.39-1776.03) | 1321.5 (808.59-2015.79) | 0.52 (0.26-0.78) |
| Republic of Lithuania | 10.26 (7.04-14.14) | 8.59 (5.38-12.68) | -0.16 | 1113.18 (764.14-1533.65) | 1499.53 (939.86-2213.81) | -0.07 (-0.34-0.19) |
| Republic of Madagascar | 28.84 (18.22-41.18) | 87.43 (52.49-132.21) | 2.03 | 1070.9 (676.55-1529.02) | 1207.08 (724.67-1825.24) | 0.04 (-0.09-0.17) |
| Republic of Malawi | 21.5 (14.09-30.18) | 52.64 (33.97-77.71) | 1.45 | 952.43 (623.88-1336.84) | 1050.75 (678.06-1551.29) | -0.17 (-0.33--0.02) |
| Republic of Maldives | 0.45 (0.3-0.63) | 0.96 (0.63-1.41) | 1.13 | 944.59 (627.3-1323.92) | 834.16 (547.11-1220.74) | -1 (-1.2--0.8) |
| Republic of Mali | 16.24 (10.64-23.18) | 46.5 (29.77-68.58) | 1.86 | 846.45 (554.29-1207.82) | 854.05 (546.73-1259.62) | -0.15 (-0.3--0.01) |
| Republic of Malta | 0.94 (0.61-1.38) | 1.08 (0.67-1.61) | 0.15 | 991.74 (643.01-1454.51) | 1146.94 (714.39-1709.54) | 0.19 (-0.02-0.4) |
| Republic of Mauritius | 3.62 (2.38-5.14) | 4.11 (2.54-6.11) | 0.14 | 1211.86 (795.77-1721.53) | 1297.51 (803.58-1929.6) | -0.44 (-0.65--0.24) |
| Republic of Moldova | 10.36 (7-14.53) | 9.21 (5.81-13.64) | -0.11 | 920.53 (622.07-1290.89) | 1044.49 (658.46-1546.53) | -0.33 (-0.6--0.07) |
| Republic of Mozambique | 33.18 (21.49-46.94) | 93.5 (59.63-141.28) | 1.82 | 1051.58 (681.05-1487.43) | 1231.55 (785.39-1860.84) | 0.1 (-0.03-0.22) |
| Republic of Namibia | 2.78 (1.81-3.92) | 7.54 (4.85-11.28) | 1.71 | 821.08 (533.24-1157.16) | 1139.94 (732.99-1705.49) | 0.14 (-0.17-0.46) |
| Republic of Nauru | 0.02 (0.01-0.02) | 0.02 (0.01-0.03) | 0 | 687.21 (454.85-1002.56) | 779.62 (490.82-1157.8) | 0.12 (0.04-0.19) |
| Republic of Nicaragua | 7.9 (5.11-11.39) | 20.16 (13.29-29.87) | 1.55 | 876 (566.34-1262.08) | 1112.08 (733-1647.72) | 0.19 (-0.01-0.39) |
| Republic of Niue | 0 (0-0.01) | 0 (0-0) | 0 | 699.4 (465.65-1014.84) | 791.7 (501.52-1179.59) | 0.15 (0.08-0.22) |
| Republic of Palau | 0.03 (0.02-0.04) | 0.03 (0.02-0.05) | 0 | 695.5 (455.71-1006.48) | 803.92 (512.75-1221.67) | 0.19 (0.12-0.25) |
| Republic of Panama | 4.62 (2.92-6.65) | 10.59 (6.62-16.09) | 1.29 | 754.57 (477.58-1086.12) | 991.06 (619.27-1505.12) | 0.39 (0.16-0.63) |
| Republic of Paraguay | 9 (5.86-12.76) | 25.51 (15.81-37.84) | 1.83 | 957.06 (623.81-1356.83) | 1344.7 (833.49-1994.46) | 0.49 (0.29-0.68) |
| Republic of Peru | 31.72 (21.11-44.33) | 72.86 (46.35-108.49) | 1.3 | 584.01 (388.65-816.05) | 757.48 (481.88-1127.87) | -0.03 (-0.41-0.35) |
| Republic of Poland | 47.86 (32.62-65.85) | 55.42 (36.88-78.14) | 0.16 | 509.49 (347.27-700.99) | 627.78 (417.74-885.11) | -0.13 (-0.44-0.19) |
| Republic of Rwanda | 22.63 (14.6-32.21) | 50.55 (31.08-77.41) | 1.23 | 1394.66 (900.09-1985.44) | 1435.54 (882.46-2198.22) | -0.64 (-0.84--0.44) |
| Republic of San Marino | 0.08 (0.05-0.12) | 0.12 (0.07-0.19) | 0.5 | 1305.01 (836.69-1948.15) | 1663.3 (995.1-2729.67) | 0.31 (0.11-0.51) |
| Republic of Senegal | 14.38 (9.59-20.26) | 39.3 (24.35-57.38) | 1.73 | 835.74 (557.08-1177.66) | 1007.39 (624.19-1470.79) | 0.25 (0.07-0.42) |
| Republic of Serbia | 18.83 (12.63-25.71) | 18.16 (11.62-27.33) | -0.04 | 806.3 (540.83-1101.19) | 893.53 (571.6-1344.49) | -0.32 (-0.53--0.1) |
| Republic of Seychelles | 0.1 (0.06-0.14) | 0.17 (0.11-0.26) | 0.7 | 531.24 (354-747.12) | 690.36 (434.4-1079.14) | 0.3 (0.12-0.48) |
| Republic of Sierra Leone | 9.91 (6.53-14.06) | 24.35 (15.13-37.74) | 1.46 | 987.34 (650.29-1400.73) | 1070.83 (665.07-1659.48) | 0.28 (0.21-0.35) |
| Republic of Singapore | 9.42 (6.44-12.85) | 9.04 (5.84-13.21) | -0.04 | 1008.27 (689.51-1374.93) | 621.28 (401.19-908.05) | -2.23 (-2.56--1.91) |
| Republic of Slovenia | 5.26 (3.54-7.27) | 4.37 (2.86-6.71) | -0.17 | 1058.02 (711.03-1461.77) | 1039.07 (680-1594.19) | -0.71 (-0.93--0.49) |
| Republic of South Africa | 105.77 (72.77-144.5) | 223.07 (155.13-308.19) | 1.11 | 1095.64 (753.82-1496.93) | 1440.27 (1001.65-1989.88) | 0.5 (0.22-0.77) |
| Republic of South Sudan | 13.79 (8.85-19.8) | 27.33 (16.99-40.21) | 0.98 | 1064.99 (683.26-1529.08) | 1177.08 (731.71-1732.12) | 0.19 (0.1-0.28) |
| Republic of Sudan | 58.23 (38.03-85.84) | 153.71 (94.75-227.16) | 1.64 | 1243.64 (812.2-1833.45) | 1361.6 (839.33-2012.27) | -0.14 (-0.31-0.03) |
| Republic of Suriname | 1.23 (0.82-1.72) | 2.67 (1.66-4) | 1.17 | 1260.69 (848.3-1770.85) | 1842.36 (1141.63-2758.67) | 0.44 (0.22-0.66) |
| Republic of Tajikistan | 8.34 (5.51-11.74) | 21.38 (13.56-31.99) | 1.56 | 691.86 (456.65-973.58) | 841.26 (533.48-1258.67) | 0.04 (-0.18-0.25) |
| Republic of the Congo | 9 (5.85-12.89) | 25.46 (15.98-37.99) | 1.83 | 1597.13 (1037.15-2286.29) | 1776.95 (1115.45-2651.82) | 0.02 (-0.15-0.19) |
| Republic of the Gambia | 3.71 (2.42-5.35) | 10.69 (6.34-15.9) | 1.88 | 1634.03 (1067.4-2357.49) | 1738.38 (1030.27-2585.86) | -0.35 (-0.51--0.18) |
| Republic of the Marshall Islands | 0.06 (0.04-0.09) | 0.1 (0.06-0.14) | 0.67 | 614.14 (406.93-885.08) | 670.12 (420.16-975.9) | -0.03 (-0.11-0.05) |
| Republic of the Niger | 17.29 (11.21-24.64) | 51.47 (34.27-78.51) | 1.98 | 994.06 (644.45-1416.21) | 965.46 (642.85-1472.59) | -0.07 (-0.12--0.03) |
| Republic of the Philippines | 100.99 (68.45-140.63) | 223.73 (150.5-312.06) | 1.22 | 650.71 (441.09-906.14) | 763.26 (513.44-1064.59) | 0.01 (-0.22-0.24) |
| Republic of the Union of Myanmar | 41.35 (27.33-59.19) | 79.43 (52.09-117.46) | 0.92 | 396.79 (262.2-567.91) | 525.38 (344.54-776.98) | 0.37 (0.11-0.63) |
| Republic of Trinidad and Tobago | 3.45 (2.24-4.81) | 4.85 (2.91-7.58) | 0.41 | 1121.49 (728.63-1564) | 1426.67 (855-2229.98) | -0.06 (-0.32-0.21) |
| Republic of Tunisia | 28.84 (18.27-41.82) | 58.53 (35.81-88.73) | 1.03 | 1410.76 (893.7-2045.53) | 1907.65 (1167.28-2891.84) | 0.29 (0.04-0.55) |
| Republic of Turkey | 175.65 (122.75-238.23) | 324.71 (195.2-506.99) | 0.85 | 1233.13 (861.76-1672.49) | 1499.73 (901.55-2341.58) | 0.28 (0.13-0.44) |
| Republic of Uganda | 58.83 (38.2-83.56) | 178.09 (111.13-268.06) | 2.03 | 1528.77 (992.81-2171.4) | 1710.17 (1067.17-2574.11) | -0.49 (-0.82--0.17) |
| Republic of Uzbekistan | 37.15 (24.87-52.56) | 77.48 (49.33-114) | 1.09 | 758.29 (507.72-1073.02) | 870.69 (554.33-1281.09) | 0.05 (-0.09-0.19) |
| Republic of Vanuatu | 0.24 (0.16-0.34) | 0.56 (0.36-0.81) | 1.33 | 681.16 (447.48-969.58) | 713.44 (454.86-1033.58) | -0.17 (-0.26--0.07) |
| Republic of Yemen | 40.64 (26.28-58.8) | 123.89 (76.12-181.41) | 2.05 | 1483.52 (959.54-2146.77) | 1482.68 (911.05-2171.15) | -0.09 (-0.15--0.02) |
| Republic of Zambia | 16.27 (10.57-23.11) | 51.08 (33.04-76.13) | 2.14 | 884.87 (574.69-1256.81) | 1038.45 (671.69-1547.71) | 0.01 (-0.13-0.15) |
| Republic of Zimbabwe | 16.12 (10.72-22.82) | 33.63 (21.77-48.71) | 1.09 | 670.35 (445.78-949.11) | 828.22 (536.12-1199.44) | 0.29 (0.1-0.47) |
| Romania | 37.74 (24.55-53.07) | 35.93 (23.13-52.1) | -0.05 | 671.23 (436.51-943.72) | 884.84 (569.54-1282.96) | 0.24 (0-0.48) |
| Russian Federation | 291.52 (192.4-409.47) | 350.94 (232.68-491.55) | 0.2 | 787.35 (519.64-1105.9) | 1035.41 (686.49-1450.26) | 0.22 (-0.08-0.51) |
| Saint Kitts and Nevis | 0.1 (0.06-0.15) | 0.21 (0.13-0.31) | 1.1 | 1004.19 (638.13-1504.61) | 1317.11 (811.49-1962.39) | 0.43 (0.29-0.58) |
| Saint Lucia | 0.27 (0.18-0.39) | 0.52 (0.32-0.78) | 0.93 | 797.13 (515.84-1154.43) | 1141.59 (699.75-1704.57) | 0.51 (0.27-0.75) |
| Saint Vincent and the Grenadines | 0.21 (0.13-0.3) | 0.3 (0.19-0.45) | 0.43 | 789.32 (490.28-1136.93) | 1074.85 (667.16-1626.33) | 0.48 (0.28-0.68) |
| Slovak Republic | 9.89 (6.69-14.1) | 12.1 (7.65-18.44) | 0.22 | 745.8 (504.41-1063.25) | 958.43 (606.22-1460.89) | 0 (-0.31-0.31) |
| Socialist Republic of Viet Nam | 101.44 (67.85-143.36) | 169.47 (109.56-245.58) | 0.67 | 594.48 (397.6-840.12) | 663.52 (428.95-961.51) | -0.14 (-0.27--0.01) |
| Solomon Islands | 0.51 (0.33-0.72) | 1.24 (0.8-1.77) | 1.43 | 680.6 (445.98-970.99) | 722.91 (466.15-1031.01) | -0.11 (-0.19--0.02) |
| State of Eritrea | 8.76 (5.6-12.46) | 20.02 (12.07-29.41) | 1.29 | 1110.85 (709.87-1580.39) | 1209.59 (729.2-1777.05) | 0.03 (-0.08-0.13) |
| State of Israel | 16.54 (10.92-23.3) | 34.04 (21.15-51.85) | 1.06 | 1356.11 (895.48-1909.91) | 1530.14 (950.73-2330.93) | -0.32 (-0.53--0.11) |
| State of Kuwait | 5.11 (3.26-7.54) | 18.73 (11.42-29.01) | 2.67 | 1234.06 (785.89-1819.92) | 1281.21 (781.42-1984.43) | -0.01 (-0.11-0.09) |
| State of Libya | 11.16 (7.17-16.25) | 28.99 (18.07-44.71) | 1.6 | 1225.68 (787.62-1784.51) | 1457.18 (908.39-2247.45) | 0.33 (0.2-0.46) |
| State of Qatar | 1.1 (0.69-1.59) | 8 (4.97-12.3) | 6.27 | 1386.97 (878.27-2015.85) | 1441.97 (895.78-2216.9) | -0.24 (-0.39--0.09) |
| Sultanate of Oman | 3.93 (2.52-5.77) | 14.56 (8.79-21.56) | 2.7 | 1159.39 (743.81-1703.5) | 1433.76 (865.96-2123.05) | 0.34 (0.13-0.55) |
| Swiss Confederation | 25.48 (17.35-35.47) | 28.23 (18.12-40.83) | 0.11 | 1447.59 (985.56-2014.78) | 1443.01 (926.32-2086.69) | -0.72 (-1.16--0.27) |
| Syrian Arab Republic | 31.3 (19.89-46.42) | 53.51 (33.32-82.5) | 0.71 | 1135.28 (721.17-1683.48) | 1375.33 (856.39-2120.63) | 0.27 (0.09-0.45) |
| Taiwan (Province of China) | 26.62 (17.93-37.62) | 34.69 (21.52-49.58) | 0.3 | 483.74 (325.86-683.72) | 617.48 (383.12-882.58) | 0.71 (0.66-0.77) |
| Togolese Republic | 8.56 (5.63-12.1) | 23.62 (14.84-35.42) | 1.76 | 1002.87 (659.59-1418.53) | 1095.44 (688.42-1642.67) | 0.16 (0.08-0.23) |
| Tokelau | 0 (0-0) | 0 (0-0) | 0 | 690.37 (451.37-1006.34) | 789.07 (488.72-1196.97) | 0.15 (0.07-0.23) |
| Turkmenistan | 6.55 (4.31-9.22) | 10.9 (6.86-15.97) | 0.66 | 740.57 (487.2-1043.23) | 867.29 (546.01-1270.68) | 0.04 (-0.13-0.2) |
| Tuvalu | 0.02 (0.01-0.02) | 0.02 (0.01-0.03) | 0 | 700.19 (453.1-1022.61) | 777.25 (487.82-1183) | 0.08 (0.01-0.15) |
| Ukraine | 137.38 (91.38-192.27) | 137.27 (88.24-197.92) | 0 | 1085.32 (721.88-1518.93) | 1358.43 (873.21-1958.54) | -0.26 (-0.49--0.03) |
| Union of the Comoros | 1 (0.66-1.42) | 2.17 (1.37-3.27) | 1.17 | 953.24 (626.09-1350.55) | 1112.3 (700.19-1674.42) | 0.11 (-0.02-0.25) |
| United Arab Emirates | 4.06 (2.63-5.81) | 23.85 (14.4-36.14) | 4.87 | 1186.12 (768.88-1696.62) | 1383.29 (835.03-2096.35) | 0.01 (-0.22-0.24) |
| United Kingdom of Great Britain and Northern Ireland | 203.07 (134-282.97) | 240.55 (159.99-338.87) | 0.18 | 1430.93 (944.22-1993.94) | 1568.2 (1042.97-2209.15) | -0.12 (-0.44-0.21) |
| United Mexican States | 169.31 (115.1-235.64) | 513.08 (342.24-708.72) | 2.03 | 773.35 (525.73-1076.32) | 1464.09 (976.61-2022.38) | 1.91 (1.64-2.17) |
| United Republic of Tanzania | 62.98 (41.25-89.58) | 174.81 (107.99-255.17) | 1.78 | 1049.57 (687.54-1492.95) | 1176.45 (726.71-1717.19) | 0.05 (-0.06-0.15) |
| United States of America | 807.8 (560.84-1107.36) | 1480.34 (1040.13-2007.74) | 0.83 | 1205.76 (837.14-1652.9) | 1955.18 (1373.76-2651.74) | 0.66 (0.38-0.94) |
| United States Virgin Islands | 0.24 (0.16-0.35) | 0.18 (0.11-0.27) | -0.25 | 849.41 (544.63-1225.42) | 1065.85 (643.9-1543.52) | 0.33 (0.15-0.5) |

**Abbreviations: WCBA, Women of Childbearing Age; EAPC, Estimated Annual Percentage Change; CI, Confidence Intervals; UI, Uncertainty Intervals; DALYs, Disability-Adjusted Life Years.**

# **Table S6. The DALYs of depression cases and rates among WCBA in 1990 and 2021, and the trends in age patterns from 1990 to 2021.**

| location | Age(years) | DALY cases | | | DALY rates | | |
| --- | --- | --- | --- | --- | --- | --- | --- |
|  |  | 1990_thousands(95% UI) | 2021_thousands(95% UI) | percentage change(100%) | 1990_per 100 000(95% UI) | 2021_per 100 000(95% UI) | EAPC(95% CI) |
| Global | 15-19 | 1593.58 (1017.79-2374.13) | 2421.17 (1522.83-3649.93) | 0.52 | 623.61 (398.29-929.07) | 797.36 (501.51-1202.02) | 0.25(0.06-0.45) |
|  | 20-24 | 2198.16 (1403.68-3266.21) | 3040.49 (1921.34-4552.66) | 0.38 | 900.39 (574.96-1337.87) | 1035.05 (654.07-1549.83) | -0.2(-0.43-0.03) |
|  | 25-29 | 2081.76 (1375.57-3019.81) | 3068.1 (1977.87-4465.91) | 0.47 | 945.83 (624.98-1372.02) | 1054.38 (679.71-1534.74) | -0.25 (-0.46--0.04) |
|  | 30-34 | 1881.03 (1218.28-2771.01) | 3205.88 (2042.4-4741.6) | 0.70 | 989.45 (640.83-1457.59) | 1072.45 (683.23-1586.19) | -0.26(-0.43--0.08) |
|  | 35-39 | 1829.2 (1198.31-2565.76) | 3271.98 (2112.19-4667.62) | 0.79 | 1054.58 (690.86-1479.22) | 1177.81 (760.32-1680.19) | -0.16(-0.33-0.01) |
|  | 40-44 | 1564.36 (1005.03-2239.26) | 3097.08 (1951.31-4452.88) | 0.98 | 1115.6 (716.72-1596.9) | 1248.37 (786.54-1794.87) | -0.15(-0.32-0.02) |
|  | 45-49 | 1297.25 (865.54-1787.26) | 2937.73 (1940.26-4062.88) | 1.26 | 1139.93 (760.58-1570.52) | 1246.69 (823.39-1724.17) | -0.16(-0.29--0.03) |
|  | 15-49 | 12445.34 (8431.57-17071.21) | 21042.42 (14072.21-29061.45) | 0.69 | 930.59 (630.46-1276.48) | 1079.73 (722.08-1491.21) | -0.06(-0.24-0.12) |
| Low SDI | 15-19 | 167.91 (100.83-256.6) | 473.52 (279.81-730.62) | 1.82 | 668.2 (401.27-1021.15) | 767.97 (453.81-1184.95) | -0.04(-0.19-0.12) |
|  | 20-24 | 214.54 (134.98-322.44) | 561.1 (350.27-857.61) | 1.62 | 987.74 (621.42-1484.47) | 1064.05 (664.23-1626.35) | -0.24(-0.39--0.09) |
|  | 25-29 | 202 (129.21-300.06) | 508.4 (321.25-760.13) | 1.52 | 1090.52 (697.54-1619.9) | 1154.17 (729.29-1725.64) | -0.27(-0.42--0.13) |
|  | 30-34 | 179.31 (113.61-271.3) | 456.71 (286.32-688.7) | 1.55 | 1178.81 (746.87-1783.61) | 1230.41 (771.36-1855.42) | -0.32(-0.47--0.17) |
|  | 35-39 | 164.34 (105.77-235.54) | 420.64 (267.55-599.45) | 1.56 | 1275.44 (820.9-1828.1) | 1320.02 (839.61-1881.16) | -0.33(-0.47--0.18) |
|  | 40-44 | 135.22 (83.89-197.59) | 368.73 (229.14-532.61) | 1.73 | 1363.8 (846.12-1992.83) | 1411.7 (877.25-2039.08) | -0.31(-0.45--0.17) |
|  | 45-49 | 120.52 (78.75-168.39) | 310.84 (203.7-433.93) | 1.58 | 1451.79 (948.64-2028.44) | 1496.79 (980.86-2089.48) | -0.31(-0.45--0.17) |
|  | 15-49 | 1183.84 (791.52-1656.89) | 3099.94 (2054.47-4324.6) | 1.62 | 1059.98 (708.71-1483.53) | 1130.08 (748.96-1576.53) | -0.24(-0.38--0.1) |
| Low-middle SDI | 15-19 | 358.93 (225.13-542.83) | 669.58 (416.86-1012.65) | 0.87 | 610.93 (383.18-923.94) | 740.68 (461.12-1120.17) | -0.04(-0.26-0.19) |
|  | 20-24 | 502.88 (320.74-755.54) | 901.74 (564.82-1355.09) | 0.79 | 964.21 (614.98-1448.66) | 1036.38 (649.15-1557.42) | -0.5(-0.73--0.27) |
|  | 25-29 | 473.15 (301.57-708.94) | 900.15 (568.15-1326.78) | 0.90 | 1054.18 (671.91-1579.53) | 1107.91 (699.29-1633.02) | -0.57(-0.8--0.34) |
|  | 30-34 | 428.48 (271.48-648.59) | 886.58 (558.85-1320.91) | 1.07 | 1144.33 (725.03-1732.19) | 1199.57 (756.14-1787.23) | -0.59(-0.83--0.35) |
|  | 35-39 | 406.8 (260.62-583.12) | 894.48 (571.1-1277.65) | 1.20 | 1269.91 (813.58-1820.31) | 1343.33 (857.68-1918.77) | -0.53(-0.76--0.3) |
|  | 40-44 | 354.81 (220-510.78) | 832.24 (520.2-1211.58) | 1.35 | 1367.56 (847.96-1968.73) | 1442.81 (901.84-2100.46) | -0.51(-0.72--0.3) |
|  | 45-49 | 307.42 (199.88-425.87) | 732.4 (474.58-1024.91) | 1.38 | 1416.34 (920.91-1962.08) | 1481.48 (959.97-2073.16) | -0.51(-0.71--0.31) |
|  | 15-49 | 2832.46 (1877.38-3934.43) | 5817.17 (3878.17-8060.39) | 1.05 | 1037.85 (687.9-1441.62) | 1149.02 (766.03-1592.11) | -0.37(-0.59--0.15) |
| Middle SDI | 15-19 | 495.78 (318.25-739.96) | 564.22 (354.78-845.16) | 0.14 | 537.7 (345.15-802.52) | 642.34 (403.9-962.18) | -0.01(-0.23-0.2) |
|  | 20-24 | 706.99 (451.47-1046.18) | 757.18 (475.4-1137.1) | 0.07 | 800.38 (511.11-1184.37) | 873.66 (548.53-1312.03) | -0.32(-0.58--0.07) |
|  | 25-29 | 628.04 (414.4-912.32) | 810.39 (523.45-1186.37) | 0.29 | 838.42 (553.21-1217.92) | 894.49 (577.77-1309.48) | -0.3(-0.52--0.08) |
|  | 30-34 | 529.39 (343.37-786.46) | 910.09 (583.15-1343.93) | 0.72 | 881.75 (571.91-1309.92) | 921.58 (590.51-1360.9) | -0.26(-0.45--0.06) |
|  | 35-39 | 528.23 (345.41-750.06) | 953.21 (611.18-1344.75) | 0.80 | 952.95 (623.14-1353.13) | 1040.08 (666.88-1467.3) | -0.19(-0.39-0.01) |
|  | 40-44 | 432.32 (274.58-616.99) | 911.79 (569.81-1310.19) | 1.11 | 1023.08 (649.79-1460.12) | 1113.96 (696.16-1600.7) | -0.23(-0.41--0.05) |
|  | 45-49 | 356.48 (237.87-493.07) | 913.08 (605.91-1262.13) | 1.56 | 1051.77 (701.83-1454.79) | 1125.72 (747.02-1556.05) | -0.21(-0.34--0.08) |
|  | 15-49 | 3677.23 (2477.85-5054.2) | 5819.96 (3876.66-7945.73) | 0.58 | 822.53 (554.25-1130.53) | 941.03 (626.82-1284.75) | -0.05(-0.24-0.14) |
| High-middle SDI | 15-19 | 292.27 (190.33-431.68) | 262.65 (157.44-399.93) | -0.10 | 617.11 (401.88-911.49) | 762.76 (457.21-1161.44) | 0.3(0.05-0.54) |
|  | 20-24 | 406.24 (260.72-601.61) | 327.51 (202.37-493.18) | -0.19 | 843.86 (541.57-1249.68) | 920.08 (568.53-1385.5) | -0.46(-0.79--0.13) |
|  | 25-29 | 398.6 (264.34-572.64) | 363.62 (231.48-537.06) | -0.09 | 871.33 (577.85-1251.78) | 902.07 (574.26-1332.34) | -0.47(-0.71--0.23) |
|  | 30-34 | 379.27 (244.96-551.22) | 457.91 (295.15-671.79) | 0.21 | 908.47 (586.75-1320.34) | 889.31 (573.22-1304.68) | -0.4(-0.57--0.23) |
|  | 35-39 | 384.35 (255.83-536.1) | 490.8 (317.01-699.85) | 0.28 | 973.11 (647.73-1357.33) | 990.5 (639.76-1412.38) | -0.29(-0.47--0.1) |
|  | 40-44 | 321.75 (206.49-457.61) | 494.5 (313.4-718.39) | 0.54 | 1046.52 (671.63-1488.46) | 1086.36 (688.49-1578.2) | -0.28(-0.46--0.11) |
|  | 45-49 | 266.85 (178.79-367.18) | 542.22 (361.72-756.8) | 1.03 | 1088.41 (729.24-1497.61) | 1124.28 (750.02-1569.22) | -0.26(-0.38--0.14) |
|  | 15-49 | 2449.32 (1660.7-3364.44) | 2939.22 (1971.66-4075.96) | 0.20 | 881.83 (597.9-1211.3) | 963.27 (646.17-1335.82) | -0.15(-0.34-0.05) |
| High SDI | 15-19 | 277.33 (180.47-409.8) | 449.42 (296.93-665.81) | 0.62 | 870.21 (566.28-1285.9) | 1544.84 (1020.65-2288.64) | 1.35(1.1-1.59) |
|  | 20-24 | 365.66 (240.68-534.58) | 490.67 (315.76-732.46) | 0.34 | 1088.98 (716.78-1592.06) | 1556.51 (1001.68-2323.52) | 0.43(0.2-0.65) |
|  | 25-29 | 378.26 (251.75-530.96) | 483.28 (314.98-692.74) | 0.28 | 1055.22 (702.31-1481.22) | 1398.4 (911.42-2004.51) | 0.13(-0.1-0.36) |
|  | 30-34 | 363 (236-520.49) | 492.28 (315.45-724.79) | 0.36 | 1022.97 (665.09-1466.82) | 1315.09 (842.71-1936.23) | 0.06(-0.15-0.28) |
|  | 35-39 | 343.95 (234.66-474.08) | 510.5 (343.29-714.96) | 0.48 | 1028.59 (701.75-1417.74) | 1345.83 (905.01-1884.82) | 0.28(0.09-0.46) |
|  | 40-44 | 318.87 (214.52-446.4) | 487.52 (327.36-680.76) | 0.53 | 1021.26 (687.07-1429.71) | 1327.97 (891.7-1854.35) | 0.4(0.23-0.58) |
|  | 45-49 | 244.74 (167.8-335.37) | 436.93 (297.44-607.5) | 0.79 | 968.65 (664.11-1327.34) | 1216.87 (828.4-1691.92) | 0.39(0.25-0.53) |
|  | 15-49 | 2291.81 (1593.59-3118.43) | 3350.6 (2325.8-4609.99) | 0.46 | 1010.91 (702.93-1375.53) | 1377.95 (956.5-1895.89) | 0.4(0.21-0.59) |

**Abbreviations: WCBA, Women of Childbearing Age; EAPC, Estimated Annual Percentage Change; CI, Confidence Intervals; UI, Uncertainty Intervals; SDI, Socio-Demographic Index; DALYs, Disability-Adjusted Life Years.**

# **Table S7. Projection of depression prevalence rates among seven age groups of WCBA(15-49 Years) from 2022 to 2040.**

| Year | Age group | Predicted ASR per 105 population (95% UI) | Sd |
| --- | --- | --- | --- |
| 2022 | 15-19 years | 4274.20(4004.62-4543.78) | 137.53843 |
| 2023 | 15-19 years | 4348.47(3976.41-4720.52) | 189.82639 |
| 2024 | 15-19 years | 4424.33(3956.15-4892.50) | 238.8664 |
| 2025 | 15-19 years | 4501.87(3937.90-5065.83) | 287.73755 |
| 2026 | 15-19 years | 4581.18(3918.98-5243.38) | 337.85689 |
| 2027 | 15-19 years | 4662.37(3897.87-5426.86) | 390.04774 |
| 2028 | 15-19 years | 4745.52(3873.58-5617.45) | 444.8642 |
| 2029 | 15-19 years | 4830.74(3845.40-5816.07) | 502.7218 |
| 2030 | 15-19 years | 4918.15(3812.78-6023.51) | 563.96012 |
| 2031 | 15-19 years | 5007.85(3775.26-6240.45) | 628.87668 |
| 2032 | 15-19 years | 5099.99(3732.40-6467.57) | 697.74704 |
| 2033 | 15-19 years | 5194.67(3683.83-6705.51) | 770.83774 |
| 2034 | 15-19 years | 5292.04(3629.15-6954.94) | 848.41515 |
| 2035 | 15-19 years | 5392.25(3567.98-7216.52) | 930.75201 |
| 2036 | 15-19 years | 5495.44(3499.90-7490.98) | 1018.1325 |
| 2037 | 15-19 years | 5601.77(3424.49-7779.05) | 1110.8566 |
| 2038 | 15-19 years | 5711.41(3341.30-8081.53) | 1209.2434 |
| 2039 | 15-19 years | 5824.55(3249.82-8399.27) | 1313.6353 |
| 2040 | 15-19 years | 5941.35(3149.53-8733.18) | 1424.4006 |
| 2022 | 20-24 years | 5728.16(5391.84-6064.49) | 171.59386 |
| 2023 | 20-24 years | 5799.44(5344.08-6254.79) | 232.32411 |
| 2024 | 20-24 years | 5884.97(5325.41-6444.53) | 285.48986 |
| 2025 | 20-24 years | 5984.87(5326.28-6643.45) | 336.01386 |
| 2026 | 20-24 years | 6088.84(5332.14-6845.54) | 386.07138 |
| 2027 | 20-24 years | 6194.91(5337.63-7052.20) | 437.391 |
| 2028 | 20-24 years | 6303.20(5340.85-7265.56) | 490.99844 |
| 2029 | 20-24 years | 6413.83(5340.42-7487.23) | 547.6547 |
| 2030 | 20-24 years | 6526.91(5335.32-7718.51) | 607.95724 |
| 2031 | 20-24 years | 6642.59(5324.69-7960.49) | 672.39662 |
| 2032 | 20-24 years | 6760.99(5307.87-8214.13) | 741.39187 |
| 2033 | 20-24 years | 6882.29(5284.27-8480.30) | 815.31462 |
| 2034 | 20-24 years | 7006.61(5253.37-8759.84) | 894.50657 |
| 2035 | 20-24 years | 7134.12(5214.71-9053.53) | 979.29266 |
| 2036 | 20-24 years | 7264.99(5167.82-9362.18) | 1069.9914 |
| 2037 | 20-24 years | 7399.42(5112.25-9686.59) | 1166.923 |
| 2038 | 20-24 years | 7537.58(5047.56-10027.59) | 1270.4165 |
| 2039 | 20-24 years | 7679.66(4973.26-10386.06) | 1380.8154 |
| 2040 | 20-24 years | 7825.89(4888.86-10762.91) | 1498.4825 |
| 2022 | 25-29 years | 6020.91(5668.89-6372.92) | 179.59819 |
| 2023 | 25-29 years | 6070.90(5595.51-6546.29) | 242.54408 |
| 2024 | 25-29 years | 6125.87(5544.64-6707.11) | 296.54836 |
| 2025 | 25-29 years | 6187.63(5508.91-6866.35) | 346.28664 |
| 2026 | 25-29 years | 6251.26(5479.86-7022.65) | 393.56757 |
| 2027 | 25-29 years | 6319.70(5457.95-7181.44) | 439.66725 |
| 2028 | 25-29 years | 6398.99(5447.30-7350.68) | 485.55695 |
| 2029 | 25-29 years | 6494.03(5450.85-7537.20) | 532.22973 |
| 2030 | 25-29 years | 6604.94(5467.02-7742.86) | 580.57235 |
| 2031 | 25-29 years | 6720.37(5484.32-7956.42) | 630.6384 |
| 2032 | 25-29 years | 6838.15(5499.02-8177.28) | 683.22994 |
| 2033 | 25-29 years | 6958.39(5509.94-8406.85) | 739.00764 |
| 2034 | 25-29 years | 7081.24(5516.08-8646.40) | 798.55212 |
| 2035 | 25-29 years | 7206.82(5516.56-8897.09) | 862.38111 |
| 2036 | 25-29 years | 7335.30(5510.61-9159.99) | 930.96269 |
| 2037 | 25-29 years | 7466.82(5497.55-9436.08) | 1004.7263 |
| 2038 | 25-29 years | 7601.54(5476.76-9726.32) | 1084.0725 |
| 2039 | 25-29 years | 7739.64(5447.65-10031.63) | 1169.3819 |
| 2040 | 25-29 years | 7881.30(5409.70-10352.91) | 1261.0233 |
| 2022 | 30-34 years | 6275.12(5908.89-6641.35) | 186.84994 |
| 2023 | 30-34 years | 6333.84(5838.50-6829.17) | 252.72066 |
| 2024 | 30-34 years | 6397.60(5791.22-7003.97) | 309.37478 |
| 2025 | 30-34 years | 6461.97(5753.79-7170.15) | 361.31659 |
| 2026 | 30-34 years | 6520.90(5716.93-7324.88) | 410.19037 |
| 2027 | 30-34 years | 6575.44(5679.59-7471.29) | 457.0659 |
| 2028 | 30-34 years | 6630.72(5645.30-7616.14) | 502.76367 |
| 2029 | 30-34 years | 6691.45(5617.27-7765.63) | 548.05125 |
| 2030 | 30-34 years | 6759.61(5596.35-7922.86) | 593.49847 |
| 2031 | 30-34 years | 6829.82(5577.36-8082.29) | 639.01341 |
| 2032 | 30-34 years | 6905.31(5562.43-8248.18) | 685.14119 |
| 2033 | 30-34 years | 6992.67(5556.71-8428.63) | 732.63329 |
| 2034 | 30-34 years | 7097.26(5563.84-8630.67) | 782.356 |
| 2035 | 30-34 years | 7219.22(5582.72-8855.71) | 834.94857 |
| 2036 | 30-34 years | 7346.14(5602.07-9090.21) | 889.83175 |
| 2037 | 30-34 years | 7475.65(5618.40-9332.90) | 947.57601 |
| 2038 | 30-34 years | 7607.89(5630.66-9585.11) | 1008.789 |
| 2039 | 30-34 years | 7742.99(5637.88-9848.11) | 1074.0373 |
| 2040 | 30-34 years | 7881.12(5639.17-10123.08) | 1143.8527 |
| 2022 | 35-39 years | 6887.15(6485.55-7288.75) | 204.89695 |
| 2023 | 35-39 years | 6893.99(6355.1-7432.86) | 274.93523 |
| 2024 | 35-39 years | 6911.82(6256.95-7566.68) | 334.11568 |
| 2025 | 35-39 years | 6943.19(6182.54-7703.84) | 388.08621 |
| 2026 | 35-39 years | 6987.69(6126.46-7848.92) | 439.40296 |
| 2027 | 35-39 years | 7044.30(6084.91-8003.69) | 489.48439 |
| 2028 | 35-39 years | 7110.95(6054.56-8167.34) | 538.97596 |
| 2029 | 35-39 years | 7183.27(6030.56-8335.98) | 588.11711 |
| 2030 | 35-39 years | 7256.30(6008.01-8504.60) | 636.88619 |
| 2031 | 35-39 years | 7323.23(5980.77-8665.69) | 684.92894 |
| 2032 | 35-39 years | 7385.25(5949.58-8820.91) | 732.48045 |
| 2033 | 35-39 years | 7448.11(5919.13-8977.08) | 780.09075 |
| 2034 | 35-39 years | 7517.10(5893.41-9140.78) | 828.40967 |
| 2035 | 35-39 years | 7594.45(5873.80-9315.10) | 877.88083 |
| 2036 | 35-39 years | 7674.13(5855.14-9493.11) | 928.05358 |
| 2037 | 35-39 years | 7759.75(5839.90-9679.60) | 979.51449 |
| 2038 | 35-39 years | 7858.73(5833.59-9883.88) | 1033.239 |
| 2039 | 35-39 years | 7977.09(5840.15-10114.04) | 1090.2764 |
| 2040 | 35-39 years | 8115.01(5858.60-10371.42) | 1151.2294 |
| 2022 | 40-44 years | 7495.95(7059.09-7932.81) | 222.88705 |
| 2023 | 40-44 years | 7521.54(6933.84-8109.23) | 299.8444 |
| 2024 | 40-44 years | 7536.70(6822.85-8250.56) | 364.21006 |
| 2025 | 40-44 years | 7542.14(6716.08-8368.20) | 421.46005 |
| 2026 | 40-44 years | 7542.24(6612.85-8471.63) | 474.17937 |
| 2027 | 40-44 years | 7543.02(6515.88-8570.17) | 524.05444 |
| 2028 | 40-44 years | 7551.30(6429.63-8672.96) | 572.27857 |
| 2029 | 40-44 years | 7571.60(6356.72-8786.49) | 619.83904 |
| 2030 | 40-44 years | 7606.75(6298.33-8915.18) | 667.5627 |
| 2031 | 40-44 years | 7656.30(6252.97-9059.63) | 715.98378 |
| 2032 | 40-44 years | 7719.13(6218.79-9219.46) | 765.47686 |
| 2033 | 40-44 years | 7792.96(6193.48-9392.45) | 816.06507 |
| 2034 | 40-44 years | 7873.04(6172.79-9573.29) | 867.47438 |
| 2035 | 40-44 years | 7953.91(6152.15-9755.66) | 919.26376 |
| 2036 | 40-44 years | 8028.10(6125.59-9930.61) | 970.66901 |
| 2037 | 40-44 years | 8096.92(6094.07-10099.77) | 1021.8607 |
| 2038 | 40-44 years | 8166.68(6062.54-10270.82) | 1073.5403 |
| 2039 | 40-44 years | 8243.18(6035.23-10451.14) | 1126.507 |
| 2040 | 40-44 years | 8328.87(6013.62-10644.12) | 1181.249 |
| 2022 | 45-49 years | 7753.28(7301.45-8205.11) | 230.52699 |
| 2023 | 45-49 years | 7788.69(7180.18-8397.20) | 310.46464 |
| 2024 | 45-49 years | 7824.25(7083.22-8565.28) | 378.07675 |
| 2025 | 45-49 years | 7859.51(6998.73-8720.29) | 439.17363 |
| 2026 | 45-49 years | 7896.35(6923.38-8869.33) | 496.4176 |
| 2027 | 45-49 years | 7931.60(6851.61-9011.58) | 551.01231 |
| 2028 | 45-49 years | 7959.49(6777.29-9141.70) | 603.16573 |
| 2029 | 45-49 years | 7976.37(6696.64-9256.09) | 652.92172 |
| 2030 | 45-49 years | 7982.94(6609.92-9355.97) | 700.52361 |
| 2031 | 45-49 years | 7983.88(6520.61-9447.15) | 746.56535 |
| 2032 | 45-49 years | 7985.53(6433.51-9537.55) | 791.84636 |
| 2033 | 45-49 years | 7995.12(6354.23-9636.01) | 837.18881 |
| 2034 | 45-49 years | 8017.44(6286.10-9748.79) | 883.33729 |
| 2035 | 45-49 years | 8055.50(6230.85-9880.15) | 930.94509 |
| 2036 | 45-49 years | 8108.81(6187.32-10030.30) | 980.35262 |
| 2037 | 45-49 years | 8176.19(6153.92-10198.46) | 1031.7698 |
| 2038 | 45-49 years | 8255.26(6128.53-10381.98) | 1085.0635 |
| 2039 | 45-49 years | 8340.95(6107.07-10574.82) | 1139.7313 |
| 2040 | 45-49 years | 8427.49(6085.11-10769.87) | 1195.0932 |

**Abbreviations: WCBA, Women of Childbearing Age; UI, Uncertainty Intervals.**

# **Table S8. Projection of depression DALYs rates among seven age groups of WCBA (15-49 Years) from 2022 to 2040.**

| Year | Age group | Predicted ASR per 10^5^ population (95% UI) | Sd |
| --- | --- | --- | --- |
| 2022 | 15-19 years | 805.35(746.50-864.21) | 30.028945 |
| 2023 | 15-19 years | 819.64(738.21-901.07) | 41.546234 |
| 2024 | 15-19 years | 834.24(731.92-936.56) | 52.202458 |
| 2025 | 15-19 years | 849.19(726.28-972.10) | 62.709778 |
| 2026 | 15-19 years | 864.49(720.64-1008.34) | 73.391557 |
| 2027 | 15-19 years | 880.17(714.68-1045.66) | 84.434703 |
| 2028 | 15-19 years | 896.25(708.16-1084.35) | 95.964874 |
| 2029 | 15-19 years | 912.76(700.93-1124.59) | 108.07639 |
| 2030 | 15-19 years | 929.71(692.85-1166.57) | 120.84641 |
| 2031 | 15-19 years | 947.13(683.82-1210.44) | 134.34255 |
| 2032 | 15-19 years | 965.04(673.74-1256.35) | 148.62742 |
| 2033 | 15-19 years | 983.48(662.51-1304.46) | 163.76146 |
| 2034 | 15-19 years | 1002.47(650.06-1354.89) | 179.80494 |
| 2035 | 15-19 years | 1022.05(636.28-1407.81) | 196.8195 |
| 2036 | 15-19 years | 1042.23(621.09-1463.38) | 214.86923 |
| 2037 | 15-19 years | 1063.07(604.39-1521.75) | 234.02167 |
| 2038 | 15-19 years | 1084.59(586.06-1583.11) | 254.34871 |
| 2039 | 15-19 years | 1106.83(566.01-1647.65) | 275.92739 |
| 2040 | 15-19 years | 1129.83(544.10-1715.56) | 298.84066 |
| 2022 | 20-24 years | 1044.82(973.10-1116.55) | 36.594251 |
| 2023 | 20-24 years | 1058.77(960.98-1156.56) | 49.893561 |
| 2024 | 20-24 years | 1075.05(954.48-1195.62) | 61.5151 |
| 2025 | 20-24 years | 1093.68(951.53-1235.84) | 72.52809 |
| 2026 | 20-24 years | 1113.10(949.65-1276.54) | 83.39038 |
| 2027 | 20-24 years | 1132.92(947.82-1318.02) | 94.438603 |
| 2028 | 20-24 years | 1153.18(945.66-1360.70) | 105.87724 |
| 2029 | 20-24 years | 1173.90(942.90-1404.90) | 117.85817 |
| 2030 | 20-24 years | 1195.10(939.32-1450.89) | 130.50182 |
| 2031 | 20-24 years | 1216.82(934.76-1498.88) | 143.90859 |
| 2032 | 20-24 years | 1239.07(929.07-1549.08) | 158.16585 |
| 2033 | 20-24 years | 1261.89(922.12-1601.67) | 173.35257 |
| 2034 | 20-24 years | 1285.32(913.82-1656.82) | 189.54252 |
| 2035 | 20-24 years | 1309.38(904.04-1714.72) | 206.80677 |
| 2036 | 20-24 years | 1334.11(892.68-1775.53) | 225.21556 |
| 2037 | 20-24 years | 1359.54(879.65-1839.43) | 244.83989 |
| 2038 | 20-24 years | 1385.72(864.84-1906.59) | 265.75282 |
| 2039 | 20-24 years | 1412.68(848.14-1977.22) | 288.03063 |
| 2040 | 20-24 years | 1440.47(829.43-2051.51) | 311.75386 |
| 2022 | 25-29 years | 1054.69(982.54-1126.84) | 36.811795 |
| 2023 | 25-29 years | 1064.36(966.26-1162.45) | 50.048028 |
| 2024 | 25-29 years | 1074.92(954.58-1195.25) | 61.394775 |
| 2025 | 25-29 years | 1086.76(945.92-1227.60) | 71.858193 |
| 2026 | 25-29 years | 1099.12(938.73-1259.50) | 81.828739 |
| 2027 | 25-29 years | 1112.39(932.91-1291.86) | 91.566872 |
| 2028 | 25-29 years | 1127.40(928.91-1325.88) | 101.26799 |
| 2029 | 25-29 years | 1144.89(927.08-1362.70) | 111.12737 |
| 2030 | 25-29 years | 1164.91(927.14-1402.68) | 121.31282 |
| 2031 | 25-29 years | 1185.76(927.36-1444.15) | 131.83473 |
| 2032 | 25-29 years | 1207.05(927.11-1486.99) | 142.82445 |
| 2033 | 25-29 years | 1228.81(926.19-1531.43) | 154.39827 |
| 2034 | 25-29 years | 1251.07(924.42-1577.72) | 166.65988 |
| 2035 | 25-29 years | 1273.85(921.63-1626.07) | 179.7035 |
| 2036 | 25-29 years | 1297.18(917.69-1676.67) | 193.6163 |
| 2037 | 25-29 years | 1321.09(912.47-1729.72) | 208.48023 |
| 2038 | 25-29 years | 1345.62(905.85-1785.40) | 224.37369 |
| 2039 | 25-29 years | 1370.80(897.71-1843.89) | 241.37294 |
| 2040 | 25-29 years | 1396.66(887.93-1905.38) | 259.55341 |
| 2022 | 30-34 years | 1072.48(999.23-1145.73) | 37.373593 |
| 2023 | 30-34 years | 1083.38(983.65-1183.12) | 50.884859 |
| 2024 | 30-34 years | 1095.24(972.75-1217.74) | 62.498071 |
| 2025 | 30-34 years | 1107.29(963.90-1250.69) | 73.159709 |
| 2026 | 30-34 years | 1118.44(955.36-1281.53) | 83.207847 |
| 2027 | 30-34 years | 1128.83(946.83-1310.83) | 92.856672 |
| 2028 | 30-34 years | 1139.34(938.87-1339.81) | 102.28042 |
| 2029 | 30-34 years | 1150.81(931.99-1369.62) | 111.63988 |
| 2030 | 30-34 years | 1163.65(926.38-1400.93) | 121.05812 |
| 2031 | 30-34 years | 1177.06(921.23-1432.89) | 130.52727 |
| 2032 | 30-34 years | 1191.44(916.76-1466.12) | 140.14446 |
| 2033 | 30-34 years | 1207.70(913.61-1501.78) | 150.04323 |
| 2034 | 30-34 years | 1226.61(912.26-1540.96) | 160.38325 |
| 2035 | 30-34 years | 1248.24(912.53-1583.95) | 171.2822 |
| 2036 | 30-34 years | 1270.76(912.79-1628.73) | 182.63787 |
| 2037 | 30-34 years | 1293.77(912.48-1675.06) | 194.53462 |
| 2038 | 30-34 years | 1317.29(911.42-1723.15) | 207.07579 |
| 2039 | 30-34 years | 1341.34(909.44-1773.24) | 220.35912 |
| 2040 | 30-34 years | 1365.96(906.38-1825.54) | 234.47791 |
| 2022 | 35-39 years | 1166.45(1086.84-1246.07) | 40.618356 |
| 2023 | 35-39 years | 1167.95(1060.47-1275.42) | 54.834201 |
| 2024 | 35-39 years | 1171.55(1040.56-1302.53) | 66.830774 |
| 2025 | 35-39 years | 1177.66(1025.20-1330.12) | 77.784664 |
| 2026 | 35-39 years | 1186.10(1013.20-1359.00) | 88.21444 |
| 2027 | 35-39 years | 1196.66(1003.78-1389.53) | 98.405665 |
| 2028 | 35-39 years | 1208.99(996.34-1421.66) | 108.49865 |
| 2029 | 35-39 years | 1222.41(990.06-1454.77) | 118.54804 |
| 2030 | 35-39 years | 1236.04(984.08-1487.99) | 128.55012 |
| 2031 | 35-39 years | 1248.67(977.35-1519.98) | 138.42669 |
| 2032 | 35-39 years | 1260.44(969.94-1550.95) | 148.21698 |
| 2033 | 35-39 years | 1272.37(962.61-1582.12) | 158.03816 |
| 2034 | 35-39 years | 1285.36(956.03-1614.70) | 168.02726 |
| 2035 | 35-39 years | 1299.90(950.46-1649.34) | 178.28498 |
| 2036 | 35-39 years | 1315.06(945.14-1684.99) | 188.73631 |
| 2037 | 35-39 years | 1331.32(940.34-1722.30) | 199.47978 |
| 2038 | 35-39 years | 1349.69(936.75-1762.62) | 210.68148 |
| 2039 | 35-39 years | 1371.03(934.86-1807.19) | 222.53089 |
| 2040 | 35-39 years | 1395.40(934.53-1856.28) | 235.13992 |
| 2022 | 40-44 years | 1259.31(1173.40-1345.21) | 43.830774 |
| 2023 | 40-44 years | 1263.85(1147.59-1380.11) | 59.31636 |
| 2024 | 40-44 years | 1266.61(1125.03-1408.19) | 72.234402 |
| 2025 | 40-44 years | 1267.72(1103.64-1431.80) | 83.715319 |
| 2026 | 40-44 years | 1267.89(1083.10-1452.68) | 94.281199 |
| 2027 | 40-44 years | 1268.24(1063.85-1472.62) | 104.278 |
| 2028 | 40-44 years | 1270.05(1046.67-1493.42) | 113.96518 |
| 2029 | 40-44 years | 1274.14(1031.98-1516.31) | 123.55291 |
| 2030 | 40-44 years | 1280.98(1019.89-1542.07) | 133.20947 |
| 2031 | 40-44 years | 1290.35(1010.01-1570.69) | 143.03038 |
| 2032 | 40-44 years | 1302.02(1001.98-1602.07) | 153.08537 |
| 2033 | 40-44 years | 1315.64(995.40-1635.88) | 163.38811 |
| 2034 | 40-44 years | 1330.43(989.60-1671.26) | 173.89086 |
| 2035 | 40-44 years | 1345.46(983.83-1707.09) | 184.50535 |
| 2036 | 40-44 years | 1359.40(977.07-1741.74) | 195.06907 |
| 2037 | 40-44 years | 1372.42(969.44-1775.41) | 205.60568 |
| 2038 | 40-44 years | 1385.61(961.73-1809.48) | 216.26245 |
| 2039 | 40-44 years | 1399.96(954.64-1845.29) | 227.20719 |
| 2040 | 40-44 years | 1415.99(948.44-1883.56) | 238.55327 |
| 2022 | 45-49 years | 1293.58(1205.33-1381.83) | 45.023082 |
| 2023 | 45-49 years | 1300.26(1180.66-1419.86) | 61.020448 |
| 2024 | 45-49 years | 1306.98(1160.90-1453.06) | 74.531729 |
| 2025 | 45-49 years | 1313.57(1143.56-1483.58) | 86.739526 |
| 2026 | 45-49 years | 1320.24(1127.83-1512.66) | 98.170145 |
| 2027 | 45-49 years | 1326.51(1112.74-1540.27) | 109.06264 |
| 2028 | 45-49 years | 1331.49(1097.32-1565.65) | 119.47014 |
| 2029 | 45-49 years | 1334.59(1080.96-1588.22) | 129.40532 |
| 2030 | 45-49 years | 1335.95(1063.67-1608.23) | 138.9169 |
| 2031 | 45-49 years | 1336.33(1046.01-1626.64) | 148.11776 |
| 2032 | 45-49 years | 1336.88(1028.82-1644.95) | 157.17638 |
| 2033 | 45-49 years | 1338.98(1013.08-1664.89) | 166.27994 |
| 2034 | 45-49 years | 1343.50(999.34-1687.66) | 175.59113 |
| 2035 | 45-49 years | 1350.91(987.83-1713.98) | 185.24169 |
| 2036 | 45-49 years | 1360.99(978.23-1743.74) | 195.28281 |
| 2037 | 45-49 years | 1373.50(970.23-1776.77) | 205.74956 |
| 2038 | 45-49 years | 1388.06(963.48-1812.65) | 216.62531 |
| 2039 | 45-49 years | 1403.87(957.35-1850.40) | 227.8183 |
| 2040 | 45-49 years | 1419.94(951.12-1888.75) | 239.19261 |

**Abbreviations: WCBA, Women of Childbearing Age; UI, Uncertainty Intervals; DALYs, Disability-Adjusted Life Years.**

#

# **FigureS1-S3.**

**S1.**DALYs rates for WCBA depression from 1990 to 2021 globally and in 5 SDI regions. **S2.**Joinpoint analysis of time trends in DALYs rates for depression in WCBA from 1990 to 2021. **S3.**Rates of DALYs per 100,000 population for 21 regions in 1990 and 2021.WCBA = Women of Childbearing Age, SDI = Socio-Demographic Index.


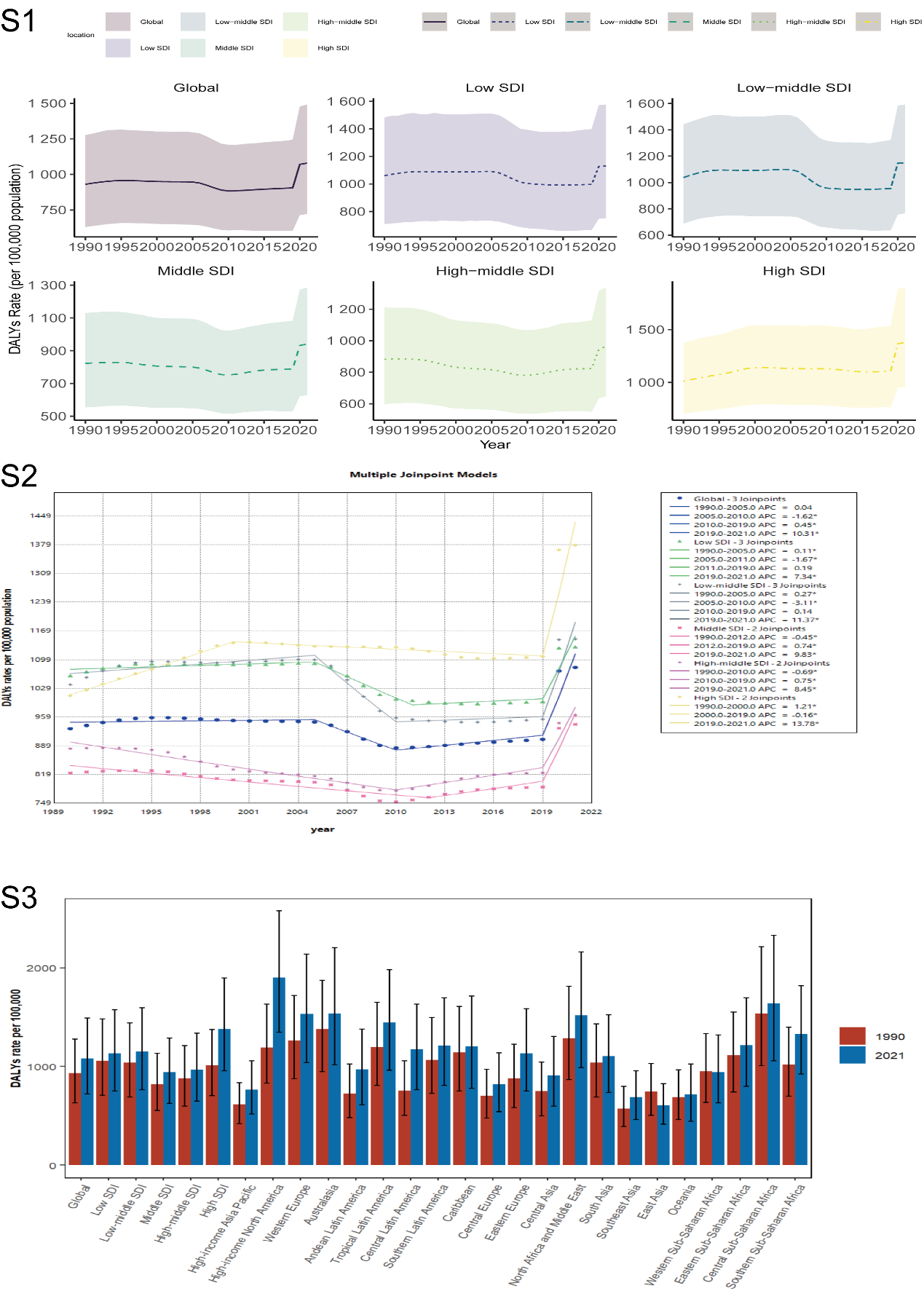


# **FigureS4.**

Temporal trends in the global WCBA burden of depression. A Percentage change in DALYs cases across 204 countries in 1990 and 2021.B EAPC in DALYs rates across 204 countries from 1990 to 2021. WCBA = women of childbearing age, EAPC = estimated annual percentage change.


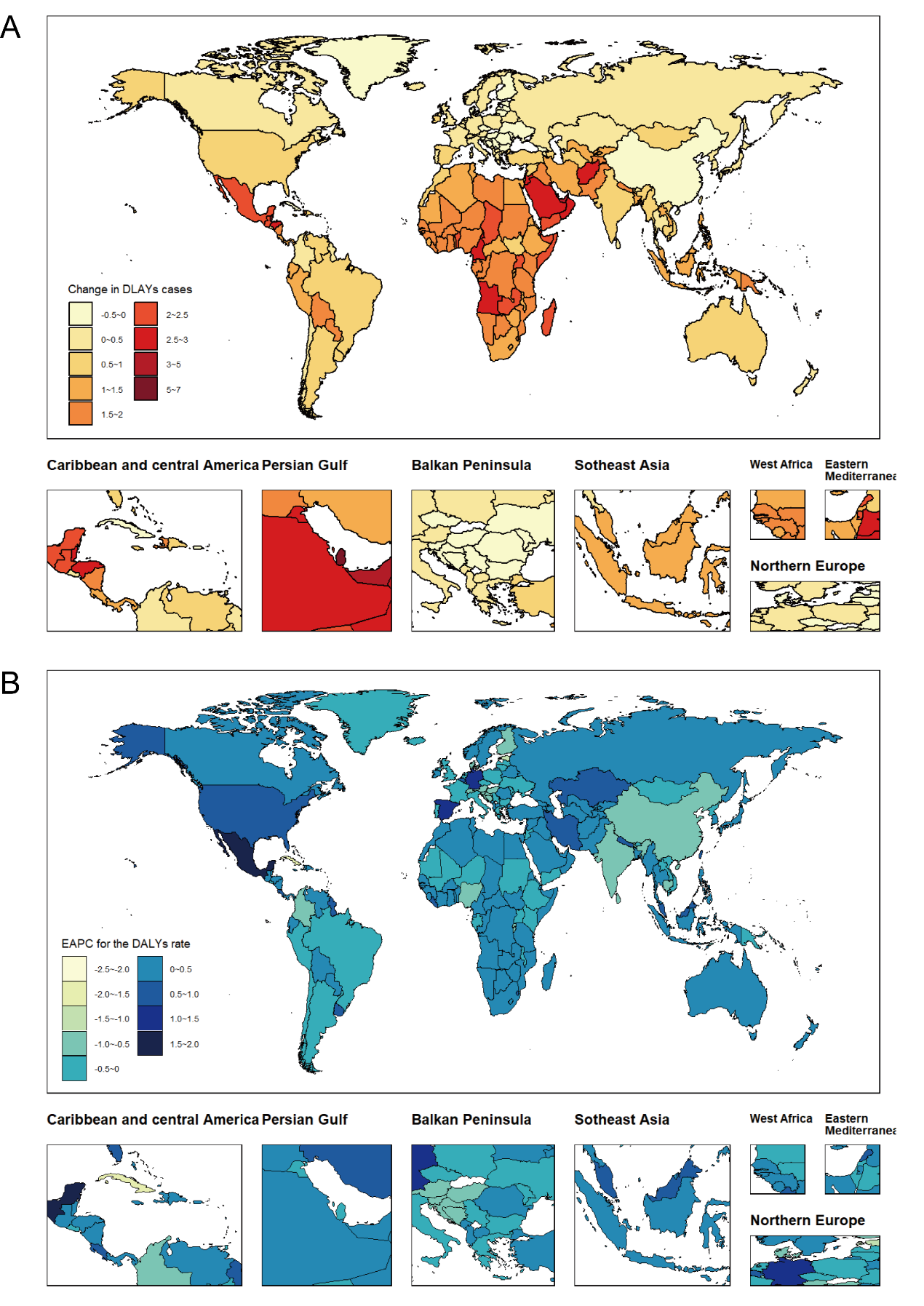


#

# **FigureS5-S9.**

**S5.**Number of cases and rates of WCBA depression DALYs in different age groups global, 1990 and 2021. **S6.** Percentage distribution of DALYs cases in 7 age groups in 5 regions and 21 GBD regions globally in 1990 and 2021. **S7.** DALYs cases of 7 age group (15-49 years,5-year intervals) from 1990 to 2021 globally and in 5 regions (low to high SDI). **S8.** Percentage change in DALYs cases of 7 age groups globally and in 5 regions in 1990 and 2021. **S9.** EAPC of DALYs rates of 7 age groups globally and in 5 regions from 1990 to 2021. WCBA = Women of Childbearing Age. GBD = Global Burden of Disease.


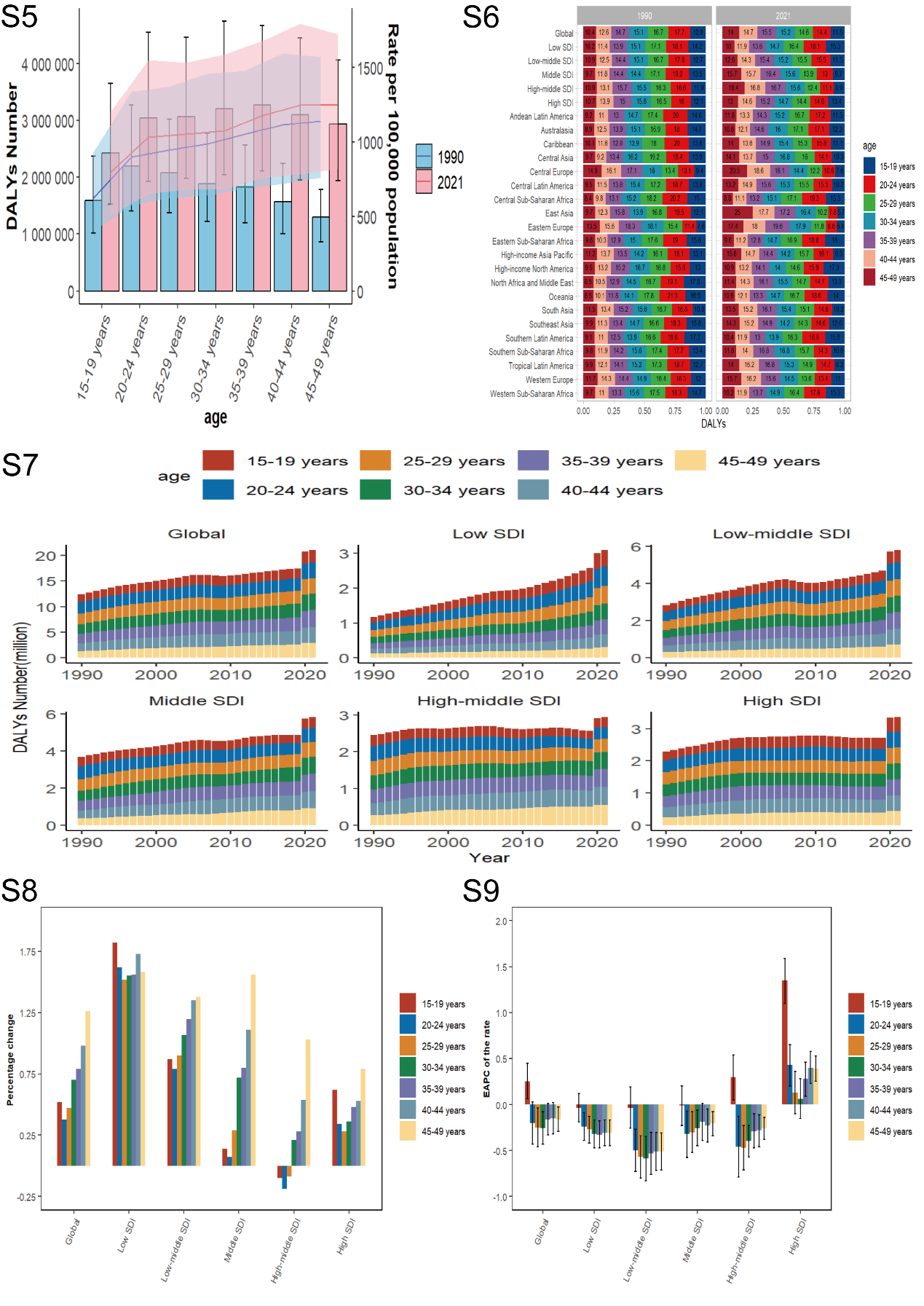


#

# **FigureS10.**

Age-Period-Cohort (APC) analysis on the DALYs of depression among different age groups (15–19,20–24,25–29,30-34,35-39,40-44,45-49 years old) across global quintiles. The age distribution of DALYs illustrates the temporal changes in the relative proportions of DALYs among different age groups from 1990 to 2021. **A.** Age-specific rates by period. **B.** Cohort-specific rates by age group. **C.** Local drift indicates the annual percentage change (percentage per year) in specific DALYs for each age group from 1990 to 2021. **D.** The age effect is represented by the fitted longitudinal age-specific DALYs after adjusting for period bias for a specific number of birth cohorts. **E.** The period effect is illustrated by the period relative risk of DALYs (DALYs ratio), calculated as the ratio of age-specific DALYs from the period 1992–1996 to that of 2017–2021. **F.** The birth cohort effect is indicated by the cohort relative risk of DALYs (rate ratio), calculated as the ratio of age-specific DALYs from the 1947 cohort to that of the 2002 cohort. Points and shaded areas represent the DALYs or ratios and their corresponding 95% confidence intervals.


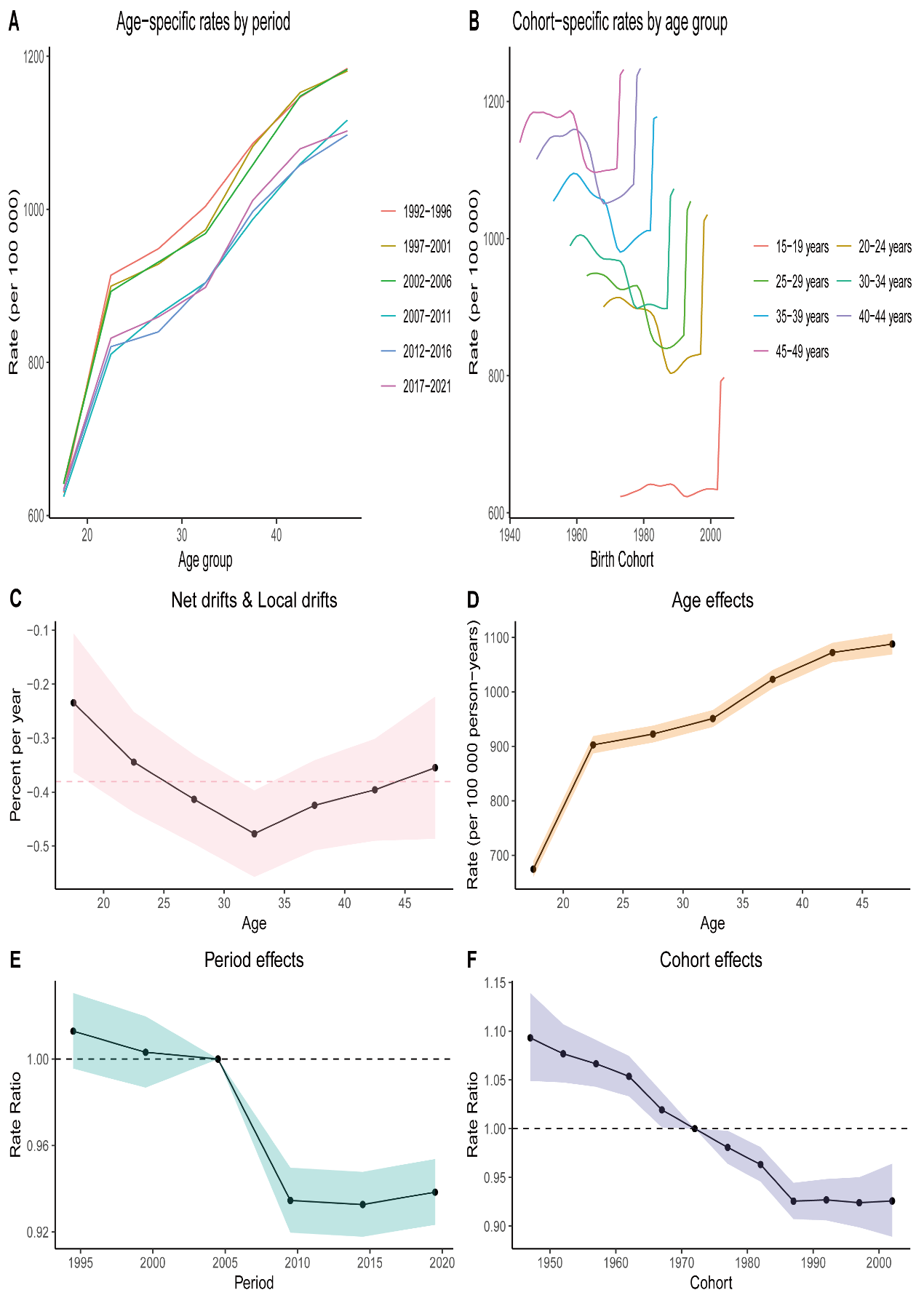


# **FigureS11.**

The projected trend of DALYs rate for depression among women of childbearing age from 2021 to 2040. The blue line represents the actual trend of DALYs rate, while the red line indicates the projected trend.

**
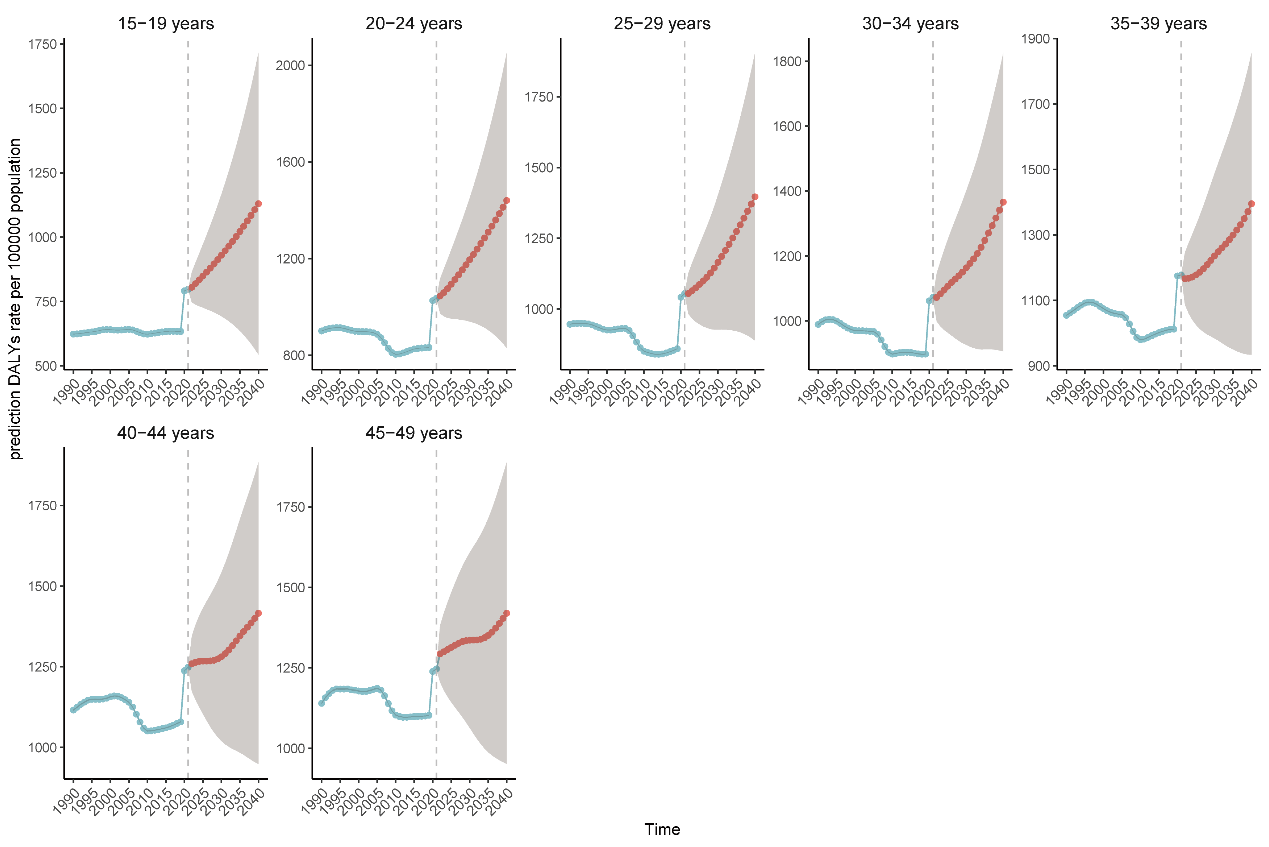
**
